# Transposon DNA sequences facilitate the tissue-specific gene transfer of circulating tumor DNA between human cells

**DOI:** 10.1093/nar/gkae427

**Published:** 2024-05-23

**Authors:** Munevver Cinar, Lourdes Martinez-Medina, Pavan K Puvvula, Arsen Arakelyan, Badri N Vardarajan, Neil Anthony, Ganji P Nagaraju, Dongkyoo Park, Lei Feng, Faith Sheff, Marina Mosunjac, Debra Saxe, Steven Flygare, Olatunji B Alese, Jonathan L Kaufman, Sagar Lonial, Juan M Sarmiento, Izidore S Lossos, Paula M Vertino, Jose A Lopez, Bassel El-Rayes, Leon Bernal-Mizrachi

**Affiliations:** Department of Hematology and Medical Oncology, Winship Cancer Institute of Emory University, Atlanta, GA, USA; Kodikaz Therapeutic Solutions, Inc, New York, NY, USA; Kodikaz Therapeutic Solutions, Inc, New York, NY, USA; Bioinformatics group, Institute of Molecular Biology NAS RA, Yerevan, Armenia; Kodikaz Therapeutic Solutions, Inc, New York, NY, USA; Integrated Cellular Imaging Core, Winship Cancer Institute of Emory University, Atlanta, GA, USA; Division of hematology and oncology, O’Neal Comprehensive Cancer Center, University of Alabama at Birmingham, Birmingham, AL, USA; Department of Hematology and Medical Oncology, Winship Cancer Institute of Emory University, Atlanta, GA, USA; Kodikaz Therapeutic Solutions, Inc, New York, NY, USA; Pathology and Laboratory Medicine, Winship Cancer Institute of Emory University, Atlanta, GA, USA; Pathology and Laboratory Medicine, Winship Cancer Institute of Emory University, Atlanta, GA, USA; Pathology and Laboratory Medicine, Winship Cancer Institute of Emory University, Atlanta, GA, USA; Department of Computational Biology/ Genetics, The University of Utah, Salt Lake City, UT, USA; Department of Hematology and Medical Oncology, Winship Cancer Institute of Emory University, Atlanta, GA, USA; Department of Hematology and Medical Oncology, Winship Cancer Institute of Emory University, Atlanta, GA, USA; Department of Hematology and Medical Oncology, Winship Cancer Institute of Emory University, Atlanta, GA, USA; Department of Surgery, Winship Cancer Institute of Emory University, Atlanta, GA, USA; Department of Medicine, Division of Hematology-Oncology and Molecular and Cellular Pharmacology, Sylvester Comprehensive Cancer Center, University of Miami, Miami, FL, USA; Department of Biomedical Genetics and the Wilmot Cancer Institute, University of Rochester Medical Center, Rochester, NY, USA; Bloodworks Northwest Research Institute, Division of Hematology, University of Washington School of Medicine, Seattle, WA, USA; Division of hematology and oncology, O’Neal Comprehensive Cancer Center, University of Alabama at Birmingham, Birmingham, AL, USA; Department of Hematology and Medical Oncology, Winship Cancer Institute of Emory University, Atlanta, GA, USA

## Abstract

The exchange of genes between cells is known to play an important physiological and pathological role in many organisms. We show that circulating tumor DNA (ctDNA) facilitates cell-specific gene transfer between human cancer cells and explain part of the mechanisms behind this phenomenon. As ctDNA migrates into the nucleus, genetic information is transferred. Cell targeting and ctDNA integration require ERVL, SINE or LINE DNA sequences. Chemically manufactured AluSp and MER11C sequences replicated multiple myeloma (MM) ctDNA cell targeting and integration. Additionally, we found that ctDNA may alter the treatment response of MM and pancreatic cancer models. This study shows that retrotransposon DNA sequences promote cancer gene transfer. However, because cell-free DNA has been detected in physiological and other pathological conditions, our findings have a broader impact than just cancer. Furthermore, the discovery that transposon DNA sequences mediate tissue-specific targeting will open up a new avenue for the delivery of genes and therapies.

## Graphical Abstract

**Graphical Abstract:**
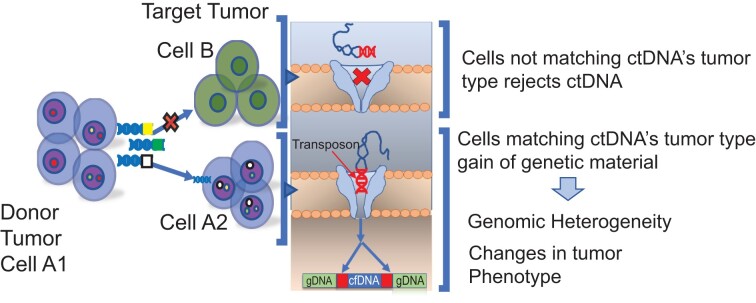

## Introduction

The transfer of genes between cells is known to play an important physiological and pathological role in many organisms. In prokaryotes, gene transfer (GT) produces changes that are more impactful in genome evolution than in branched trajectory (1). Acquisition of novel traits through GT can provide prokaryotes with survival and evolutionary advantages against environmental stressors (2,3). GT in bacteria occurs by multiple molecular mechanisms. Among them, conjugation and transformation take place through mobile genetic elements such as transposons (2,4–7).

The exchange of genetic material via transposons is a ubiquitous method for GT in some eukaryotes such as insects and plants. From the initial discovery of transposon-mediated genetic exchange between rice and millet plants (8), much evidence of transposon-mediated GT has been detected in plants, particularly parasitic plants. Acquisition of the host plant's genetic material allows parasitic plants to evolve more rapidly to adapt to new and changing environments. In fact, on many occasions, the exact moment of transposon-mediated GT defines the branch point in the evolution of different versions of the invasive plants (9). Studies have documented similar instances of transposon-mediated GT in various eukaryotic genomes, including Drosophila, by specific transfer vectors (10–12).

In contrast to the examples above, the evidence in humans for GT and any potential mechanism involved are less well established. Under physiological conditions, a few studies have shown the relocation of non-gene-coding regions between human cells (13,14). In immunology, early data suggest that the exchange of cell-free DNA from a T cell can elicit the synthesis of antibodies by B cells (14). In pathological conditions such as cancer, the evidence is even more rare. The discovery that tumor-derived cell-free DNA can contain genetic alterations relevant to tumorigenesis (15) led researchers over the last decade to evaluate the possibility that circulating tumor DNA (ctDNA) serves as a vehicle for genetic exchange between tumor cells (16). Their findings imply that genetic gene transfer can occur and influence tumor phenotype by transferring oncogenic genes or changing the tumor microenvironment (17–19). However, definitive evidence of ctDNA-mediated GT and the mechanism involved has been missing until now.

In this study, we found that specific transposon DNA sequences mediate the process whereby ctDNA targets and transmits genetic material to cells that resemble their cell of origin. These discoveries lay the framework for a new field of study on the role of ctDNA in cell-cell communication and its ramifications in fields such as embryogenesis, cell evolution, cancer, immunology, and therapeutic gene transfer.

## Materials and methods

### Clinical specimens and sample preparation

We obtained retrospective plasma samples from ten multiple myeloma (MM), ten pancreatic cancer (PC), three colon cancer (CC) and two lung cancer (LC) patients from samples stored in the Tissue and Acquisition Bank of the Winship Cancer Institute of Emory University. All patients provided written informed consent approving the use of their samples under Institutional Review Board approval. The 10 patients with newly diagnosed MM were treated with bortezomib-containing regimens (among them were five responders and five non-responders). Of the 10 PC patients, seven with advanced staged cancer were treated with gemcitabine (at the time of obtaining the plasma sample, two patients were in partial response and five had progressive disease), and three early-stage patients had undergone surgical resection. There was no data associated with the staging of the colon and lung cancer patient samples. Response in MM was determined using the International Uniform Response Criteria for MM, and PC patients were evaluated using the Response evaluation criteria in solid tumors (RECIST) criteria. Plasma was isolated after centrifuging blood samples at 1500 rpm for 10 min. The plasma supernatant was collected for storage. Plasma from non-cancer patients was purchased from Innovative Research, MI.

Bone marrow samples for the coculture of transposons were obtained from fifteen MM patients at various statuses of the disease (four newly diagnosed, three patients post-bone marrow transplant, and eight patients at relapse) and five patients without myeloma (two patients newly diagnosed with myelodysplastic syndrome, two patients post-treatment of acute myeloid leukemia and one patient with diffuse large b cell lymphoma without marrow involvement)

### Cell lines and reagents

The following cell lines were grown in Roswell Park Memorial Institute (RPMI) 1640 medium: Four MMs (OPM1, RPMI, JK6L and MM1S), one PC (ASPC-1), and one lung cancer (A549). CC cell lines (HCT-116 and HT-29) were cultured in McCoy media. Pancreatic cancer cell lines (PANC1 and MIA Paca-2 [MIA]) were cultured in DMEM media, and CC cell line RKO was cultured in EMEM. All culture media were supplemented with 10% fetal bovine serum (FBS), 1% l-glutamine, one mM sodium pyruvate, and 50 μg/ml penicillin–streptomycin. In MIA cells, 2.5% horse serum was also added to the culture medium.

Bortezomib, gemcitabine, ganciclovir, and DNaseI were purchased from Sigma-Aldrich. RNase A, 4,6-diamidino-2-phenylindole (DAPI), CellLight Plasma Membrane-GFP, Bacman 2.0, and Platinum Polymerase High Fidelity PCR Kit were purchased from Thermofisher-Scientific. DNA-PK inhibitor II and Raltegravir were purchased from Santa Cruz Biotechnology, KU-55933 (ATM kinase inhibitor) from Selleckchem, and NU1025 (PARP inhibitor VI) from Calbiochem. Quick Ligation Kit was purchased from New England Biolabs. Proteinase K, QIAamp MinElute ccfDNA Kits protocol, and QIAmp DNA Blood Mini Preb Kit were purchased from Qiagen. Cell TiterBlue Cell Viability Assay Kit was purchased from Promega. Label IT Cx-Rhodamine and Cy5 were purchased from Mirus Bio. pLenti-III HSVtk Lentivirus vector was purchased from Applied Biological Materials (abm).

For the experiments assessing inhibition of cell captured caused by antibodies produced *in vitro* and under pathogenic settings, we use anti-double-stranded DNA (dsDNA) antibodies kindly provided by Dr. Ignacio Sanz's laboratory. There are no commercially available human-derived anti-dsDNA antibodies, and assessing inhibition with a mouse-derived antibody would not answer our purpose of targeting human ctDNA. We chose antibodies from SLE patients, an illness in which anti-dsDNA antibodies are common. The chosen IgG antibodies were cloned from memory B cells isolated from two SLE patients, as previously described (20). The heavy chain of 88F7 is VH4-34, the light chain is IGKV3-20, and it possesses significant anti-dsDNA reactivity. IGHV4-59 and IGL4-69 are present in 627E6.

### Cell viability

Cell viability assays were performed in 96 well, black, clear-bottom microplates. For MM cell line studies, 3 × 10^4^ cells were cultured for 24 h with media containing 10% human plasma of a bortezomib resistant or -sensitive patient or a control non-cancer patient. Cells were then cultured for 24 h with titrating concentrations of bortezomib (doses: 0, 5, 10, 15, 50 nM,, Sigma). For PC cell line viability studies, 1 × 10^4^ cells were incubated for 24 h with media containing plasma of a gemcitabine-resistant patient or non-cancer patient. Subsequently, titrating concentrations of gemcitabine (doses: 0, 10, 25, 50, 100, 200 μM, Sigma-Aldrich, MO) were added to the culture media, and cells were then incubated for 96 h. CellTiter-Blue Cell Viability Assay was used to evaluate cell viability according to the manufacturer's instructions (Promega). Cell viability was measured by a fluorescent protocol in a microplate reader. Experiments were performed in triplicate of three independent studies.

### ctDNA extraction and immunofluorescence labeling

Isolation of ctDNA was performed following the QIAamp MinElute cfDNA Kits protocol (Qiagen, Cambridge, MA). The ctDNA was fluorescently labeled with either Cx-rhodamine or CY5 using the Label IT^®^ Nucleic Acid Labeling kit (Mirus Bio LLC, WI).

### Image acquisition

1 × 10^6^ cells cultured in 1 ml of culture media were incubated with rhodamine, CY5, and rhodamine or Cy5-labeled DNA at different time points. ctDNA, controls and transposons DNA sequences were added directly to the media without any transfection reagent. The methodology used to perform immunofluorescence in suspension cell culture was conducted as previously described (21,22). Adherent cells (2.5 × 10^5^ cells) were grown on a coverslip prior to processing. After cells were attached to slides, slides were washed with PBS twice, and the cells were then counterstained with 4,6-diamidino-2-phenylindole (DAPI) for nuclear detection (ThermoFisher MA). For live-cell imaging, the plasma membrane was labeled following the cellLight Plasma Membrane-green fluorescent protein (GFP), Bacman 2.0 protocol (ThermoFisher MA). The presented images are from triplicate experiments. Images were acquired using a Leica SP8 LIGHTING confocal microscope housed in the Cell Imaging and Microscopy Shared Resource, Winship Cancer Institute of Emory University.

Lattice light-sheet microscopy was used to obtain live images and movie acquisition presented in Supplementary Videos S1–S3. Images were acquired using a 3i v1 Lattice Light Sheet microscope in sample scanning mode, with Δs of 0.8 μm and 71 steps, and a 20 μm x-dither scan of the lattice pattern created with a 0.550 outer NA/0.500 inner NA annuli. Volume data were collected using a Hamamatsu ORCA-Flash 4.0 v2 via a Semrock FF01-446/523/600/677 blocking filter for both 488 and 560 nm laser channels (5% and 10% power, respectively) every 3 min for 1–2 h. Raw data was deskewed using 3i SlideBook 6 software to create correctly orientation volumetric data. The 3D visualization, surfaces, and movies were created in Bitplane Imaris 9. Isosurface settings were selected by the user for each dataset to represent signal boundaries efficiently.

### Nuclear localization quantification and image analysis

For quantification of the nuclear localization of rhodamine- or Cy5-ctDNA, we obtained 10 images per sample in fields with a minimum of 10 cells. Volumetric data sets were acquired using a Leica SP8 confocal microscope. All data were acquired using the same *x*, *y* and *z* sampling and with the same *xy* zoom. Z-stack total heights were varied to encapsulate the thickness of the randomly selected field of view. Data were analyzed using Fiji (24), ilastik (25) and Matlab computational software (MathWorks Inc, MA). A Fiji macro was used to convert raw.lif files as required; ilastik machine learning models were trained and then applied to classify specific nuclear morphologies; and Matlab was used to process resulting probability maps and quantify rhodamine- or Cy5-DNA signal within the nuclei. Further information about image processing and quantification of the ctDNA nuclear localization is available at GitHub repository (https://github.com/nranthony/nuc_ctDNA_process). The nuclear intensity fold change value was calculated by measuring the nuclear intensity produced by the rhodamine-ctDNA over the background rhodamine intensity of the nuclear signal in control cells.

### Chromosome spreads and ctDNA banding identification

Rhodamine-labeled ctDNA from patients with MM (*n* = 3), PC (*n* = 3) and CC (*n* = 3) was added to culture media. Briefly, 1 × 10^6^ cells cultured in 1.5 ml of medium were incubated with 1 μg/ml of rhodamine-ctDNA. At 24 h, the cells were transferred to a 15 ml tube and incubated in 10 ml of medium containing 15 μl Colcemid (10μg/ml) at 37°C for 20 min before harvesting. After centrifugation and media removal, cells were resuspended with 10 ml of pre-warmed 0.075 M KCl and incubated at 37°C for 20 min. Two ml of fixative (3:1 methanol:acetic acid) were added and incubated for 10 min before centrifugation and aspiration. Samples were then resuspended in 10 ml of fixative and incubated at room temperature for 10 min, followed by two additional washes with a fixative. Slides were prepared in a Thermotron chamber where temperature and humidity were controlled for optimum metaphase spreading. Serial micro-pipetting was performed, 3 μl at a time, until at least 25 cells were visible per field at 20× magnification. After drying slides at room temperature for 1 h, nuclei were stained with DAPI plus antifade reagent, and coverslips were applied to slides. Cytogenetic technicians performed the readout. Ten to twenty metaphase nuclei were counted per experiment, with touching and overlapping cells excluded. The number of chromosomes with integrated rhodamine bands was counted.

### Assessment of ctDNA integration with non-homologous end-joining repair, the alternative pathway, and transposase inhibitors

To investigate the mechanisms involved in ctDNA integration into the chromosomes of MM1s, ASPC-1 and HTC116 cells, 1 × 10^6^ cells were treated with inhibitors of the non-homologous end-joining (NHEJ) repair system. The inhibitors were ataxia-telangiectasia mutated (ATM) inhibitor KU-55933 (10 μM, Santa Cruz Biotechnology, TX) and the DNA-dependent protein kinase, catalytic subunit (DNA-PKCS) inhibitor I (30 μM, Sigma-Aldrich, MO). In addition, we used inhibitors for alternative NHEJ repair pathways, also known as microhomology-end joining, such as the poly ADP ribose polymerase (PARP) inhibitor NU1025 (200 μM, Sigma-Aldrich, MO). After 2 h post-drug treatment with inhibitors, rhodamine-labeled ctDNA was added to the culture media and incubated for an additional 24 h. Cell growth was then arrested, and chromosome spreads were performed as noted above. The number of rhodamine-ctDNA integration sites for each cell was determined by counting a minimum of 10 cells in metaphase. The ctDNA band integration identification and integration counts was performed by personnel from the cytogenetic laboratory of Emory University.

### Xenograft experiments

Mice were housed in a clean facility with an ambient temperature of 18–24°C, 40–60% humidity, and 12 light/12 dark cycles. All experiments included male and female animals. The protocols followed were approved by the Emory University Institutional Animal Care and Use Committee and compliant with ethical regulations for studies involving laboratory animals.

Pilot and validation xenograft studies were performed to evaluate the accumulation of ctDNA in tumors in mice. For the pilot time-course study, three mice bearing PC-MIA cell xenografts were generated by injecting 1 × 10^6^ cells in the dorsum of J:NU (007850) outbred nude mice. After tumors reached a volume of 0.5 cm, mice were assigned to a specific experimental arm: two mice underwent tail vein injection with rhodamine-labeled ctDNA, and a third mouse received a tail-vein injection with rhodamine-PBS as a control. Tumors from tail vein ctDNA-injected mice were harvested 24- and 48-h post-injection. For mice in the control group, the tumor was harvested 48 h post-injection. For the validation study, xenograft models were developed using human-derived PC (MIA), MM (MM1s) and CC (HCT-116) cell lines. Following the pilot study protocol, three mice per tumor xenograft were then dosed to assess tumor localization of rhodamine-labeled ctDNA and control (rhodamine-PBS alone). At harvest time, tumors and selected organs (liver, lung, large bowel, pancreas, and spleen) underwent frozen section dissection. Each slide was fixed with paraformaldehyde 4% and stained with DAPI before mounting the coverslip.

### Whole-genome sequencing and whole exon sequencing

ctDNA was extracted from ten MM and ten PC patients using the methods described above. DNA from CD138(+) cells and from cell lines used in *in vitro* experiments (MM1s and MIA) was extracted using the Blood & Cell Culture DNA Mini Kit (Qiagen, MD). In coculture conditions, cells were washed twice with PBS and cultured with DNase 1 for 1 min prior to a third wash. Any potential RNA contamination was removed using RNAse treatment step included in the DNA extraction kit protocol. DNA from fresh frozen paraffin-embedded pancreatic tumors was obtained using QIAamp DNA FFPE Tissue Kit Print (Qiagen, MD).

After extraction, ctDNA was ligated to the PACBIO adaptor (GCGCTCTGTGTGCT) using the ABM DNA Library Prep Kit for Illumina Sequencing (Applied Biological Materials Inc. Canada). PACBio-labeled ctDNA and regular ctDNA were subjected to standard methods for library preparation and sequencing using Illumina and Agilent protocols, respectively. Applied Biological Materials Inc. prepared the libraries and performed whole-exon and -genome sequencing. The average target coverage was 50X.

### Nucleotide variance concordance between tumor and ctDNA

#### Quality control and alignment to reference genome

The raw sequence data was subjected to quality control checks using FastQC (https://www.bioinformatics.babraham.ac.uk/projects/fastqc/). Samples that failed the QC checks were trimmed using BBDuk (https://jgi.doe.gov/data-and-tools/bbtools/bb-tools-user-guide/bbduk-guide/) in the adapter trimming mode for paired reads. The sequence reads were then aligned to the human genome GRCh38 assembly (https://www.ncbi.nlm.nih.gov/assembly/GCF_000001405.39) using the Falcon Accelerated Genomics Pipeline (https://github.com/falconcomputing/falcon-genome). This is an accelerated version of the GATK Best Practices Pipeline. Beginning with paired-end FASTQ sequence files, the first step mapped the sequences to the reference. The resulting mapped BAM file was sorted and duplicates marked. We ran the GATK 4.1.3 best practice somatic mutation pipeline with base recalibration, with read orientation filtering to account for damage seen when using FFPE samples and (https://www.intel.com/content/dam/www/programmable/us/en/pdf/literature/wp/wp-accelerating-genomics-open1-fpgas.pdf, https://www.intel.com/content /www/us/en/healthcare-it/solutions/genomicscode-gatk.html and https://pdfs.semanticscholar.org/e85d/4f927d91e9f25b7c20de71f91c78250771bb.pdf).

#### Variant calling and annotation

Variant calling was done using VarDict (https://academic.oup.com/nar/article/44/11/e108/2468301), a novel and versatile variant caller for next-generation sequencing in cancer research. VarDict was chosen based on the recommendations from Sandmann *et al.* (23). We used an allele frequency threshold of 0.01. Variants for 6 of the samples (for which a control samples were not available) were called in single sample mode (https://github.com/AstraZeneca-NGS/VarDict). For the samples where controls were available, paired mode was run in order to distinguish between somatic and germline variants. The called variants were annotated using SnpEff (http://snpeff.sourceforge.net/SnpEff.html), which is a variant annotation and effect prediction tool. We used SnpEff's pre-built GRCh38.86 database for the annotations.

#### Analysis for concordance of ctDNA and primary tumor sample

Single nucleotide variant concordance between ctDNA and corresponding tumor samples was analyzed using bcftools isec (http://samtools.github.io/bcftools/bcftools.html) to obtain concordant variants.

### Bioinformatic approach for de Novo Assembly, identification tissue-specific insertions of ctDNA and identification of transposable elements

To identify insertion in the coculture conditions, we used two different approaches for assembling the genome. Two methods for de novo assembly and analysis were employed to avoid variances that can occur in each algorithm. Please refer to supplemental methods for further details about methods of de novo assembly, contig analysis and identification of transposon sequences, and transposon expression. Whole genome and RNA sequencing data is available in SRA under accession number: PRJNA1043708 and PRJNA1070019.

### Transposon linearized vector and cell incorporation

A polynucleotide comprising sequences corresponding to the transposons sequences identified and shared by all the MM samples were generated by Integrated DNA Technologies, Inc (IDT). The sequence of these oligos is described in the Supplementary Methods Table S5. Similar methods were used to generate deletion mutants from AluSp.

Cell capture of the transposons DNA sequences was performed by flow cytometry and microscopy methods after dropping directly into the media the linear double-stranded DNA. One million MM1s or U266B1 cells were cultured at various time points, as indicated in each figure, with 1 μg of the corresponding CY5 labeled DNA retrotransposon oligos. In experiments using plasma cells from MM patients, CY5-DNA transposon oligos were cultured for 14 h prior read out.

### Statistical analysis

Two-side Student's *t*-test was used as a statistical analysis method for evaluating the difference between ctDNA nuclear localization among cells, the number of base gain in match and mismatch coculture sequencing experiments, and the number of ctDNA integrations in chromatids under the different experimental conditions. Statistical analysis for transposon enrichment was performed using Chi-square test as noted above.

## Results

### ctDNA incorporation into tumor cells resembling its cell of origin

**Figure 1.**
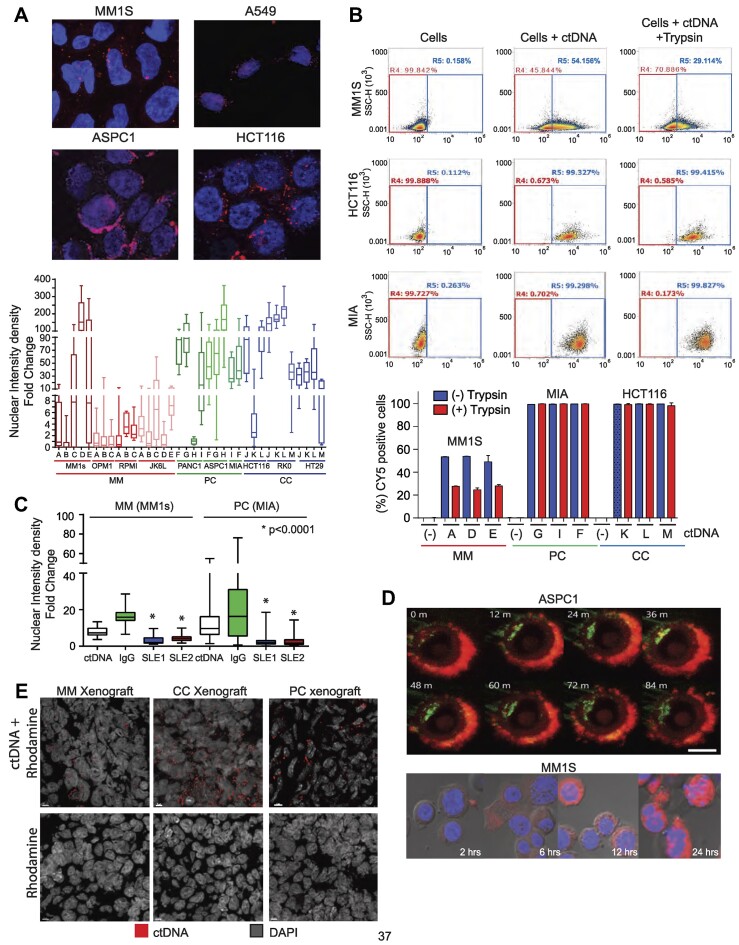
ctDNA incorporation into tumor cells. (**A**) Index images showing the nuclear localization of rhodamine-labeled ctDNA (red) in multiple myeloma (MM1s) and lung (A549), pancreatic (ASPC1), and colon (HCT116) cancer cell lines. The lower panel shows whiskers plot summarizing fold change nuclear density derived from the comparison of cell lines cultured with ctDNA derived from patients with MM (*n* = 5), pancreatic (*n* = 3) or colon (*n* = 3) cancer and the background nuclear intensity of untreated control cells. Letters on the x-axis refer to individual patient samples. (**B**) Flow cytometry assay demonstrated the percentage of cells with ctDNA incorporation. One million myeloma (MM1s), pancreatic (MIA), and colon (HCT116) cancer cells were cultured with CY5-labelled ctDNA (1 μg/ml) for 24 h. To determine ctDNA internalization, cells were then treated for 30 min with trypsin (100 μl of 0.25% Trypsin) to remove any ctDNA bound to membrane proteins. (Lower panel) Box plot summarizes triplicate experiments’ data of each cell line cultured with multiple ctDNA samples derived from patient samples. Letters on the x-axis refer to individual patient samples. (**C**) Anti-double stranded DNA antibodies (Anti-dsDNA) reduce ctDNA nuclear localization in MM (MM1s) and PC (MIA) cells. Nuclear intensity density fold change of rhodamine-labeled ctDNA in cells after cultured with PBS (control), IgG (control), and anti-dsDNA antibodies from SLE patients 1 (SLE1) and 2 (SLE2). Images were taken 24 h after coculturing antibody-ctDNA with cells. The nuclear signal was measured using similar methods as described in (A). (**D**) Time course of cytoplasmic and nuclear localization of rhodamine-ctDNA from patients with PC and MM in ASPC-1 (upper) MM1s (bottom) cells, respectively. In ASPC-1 cells, the membrane was labeled with GFP (green), and ctDNA was labeled with rhodamine (red). In MM1s cells time course, the nucleus was labeled with DAPI (blue) and ctDNA with rhodamine (red). (**E**) Tumor localization of rhodamine-ctDNA and rhodamine alone (control) 48 h after tail injection (representative images from triplicate experiments). Images in all samples were taken with an open red channel for rhodamine detection. MM: multiple myeloma, PC: pancreatic cancer; CC: colon cancer.

To determine whether cell free DNA (cfDNA) is capable of targeting cancer cell lines, we first evaluated the proportion of tumor derived-DNA detected in the blood of patients with multiple myeloma (MM), metastatic pancreatic cancer (PC), and colon cancer (CC). Exon and whole-genome sequencing from primary tumors and matching cfDNA from PC and MM patients found that 80% of the cfDNA was of tumor origin (circulating tumor DNA, ctDNA) based on the presence of tumor single nucleotide variants (SNV) in the cfDNA sequences (Supplementary Figure S1A). Based on these findings, we set out to investigate whether ctDNA can target cancer cells and translocate into the nucleus. To do so, we introduced rhodamine-labeled ctDNA from patients with MM [*n* = 4], pancreatic [PC, *n* = 3], colon [CC, *n* = 3] and lung cancer [LC, *n* = 1] or rhodamine (control) into the culture media of cell lines that matched the ctDNA’s tumor tissue of origin. The ctDNA localized in the nucleus of all of the corresponding tumor cell types (Figure 1A, Supplementary Figure S1B and Supplementary Video S1A and B) at different levels compared to the control rhodamine signal. These quantitative results were further confirmed using flow cytometry. As shown in Figure 1B, more than half of MM cells (MM1s: 52–54%) and almost all PC (MIA: 99%) and CC (HCT116: 99%) cells were in contact with ctDNA (Figure 1B). Alternatively, cells were treated briefly with trypsin in order to remove the loosely or non-specifically membrane-bound ctDNA and assess the percentage of cells that internalized ctDNA. Cellular intake of Cy5 labeled ctDNA was observed in 20–30% of MM1 cells and nearly all MIA (99%) and HCT116 (99%) cells. The capacity of cells to capture ctDNA remained unaffected by the lack of serum, treatment with proteases or by ctDNA size reduction following sonication (data not shown). These startling results demonstrate the efficiency of ctDNA in targeting tumor cells.

To begin understanding the mechanism by which cancer cells capture ctDNA, we employed a method to block the ctDNA cell recognition signal using monoclonal antibodies against double-stranded DNA (dsDNA) extracted from patients with systemic lupus erythematosus (SLE (24)). In vitro cultures of ctDNA extracted from multiple myeloma (MM) and pancreatic cancer (PC) were incubated overnight with two distinct anti-dsDNA antibodies (20). Subsequently, the ctDNA-antibody was added to the cultured cells for 24 h prior evaluating the nuclear localization of ctDNA. Anti-dsDNA antibodies significantly decreased rhodamine-labeled ctDNA nuclear signals compared to the IgG isotype control (ctDNA nuclear intensity: 16.5 to 3.23–4.2 in MM and 25.6 to 2.06–2.8 in PC, *P*< 0.0001, Figure 1C) or PBS (7.9 to 25.6 to 2.06–2.8 in MM and 14 to 2.06–2.8 in PC, *P*< 0.0001). To corroborate the capacity of ctDNA to transport and integrate non-orthologous genetic material into target cells, a linearized CMV-Green Fluorescent Protein (GFP) vector was ligated into the middle of a ctDNA fragment of a myeloma patient and added this linearized product into MM1s cells in culture. A GFP signal was detected in cells cultured with the ctDNA-containing CMV-GFP construct, whereas cells cultured with CMV-GFP alone did not show a GFP signal (3.3% versus 0.75% positive cells, *P*< 0.03, Supplementary Figure S1C). These data provide further evidence that ctDNA can mediate GT between cancer cells.

Next, we investigated the effect of time course on ctDNA uptake in MM and PC cell lines. ctDNA from a PC patient interacted with the cell membrane within seconds, was internalized into the cytoplasm within minutes, culminating in localization within the nucleus in under 12 min, as observed in ASPC-1 plasma cell line (Figure 1D and Supplementary Video S2A-C). Interestingly, ctDNA from a MM patient was captured and internalized at a much slower pace. MM ctDNA reached the cell membrane of MM1s cells in 2 h, internalized into the cytoplasm in 6 h, and reached the nucleus in 8 h, with a maximal nuclear localization at 24 h (Figure 1D).

### ctDNA preferentially migrates to tumor cells *in vivo*

To test *in vivo* the hypothesis that ctDNA targets tumor cells, we created xenograft models utilizing PC (MIA), MM (MM1s) and CC (HCT-116) cell lines. In a pilot study using a PC xenograft, rhodamine-labeled PC ctDNA or PBS-Rhodamine (control) was injected into the tail of the mice. Results determined that the maximum tumor localization of rhodamine-labeled ctDNA was detected at 48 h post-tail vein injection (Supplementary Figure S1D). Based on this information, we investigated the tumor location of ctDNA in these tumor models. Rhodamine-labeled circulating tumor DNA (ctDNA) obtained from patients with MM, PC, and CC or rhodamine alone (control) was administered intravenously into mice with tumor xenografts (*n* = 3 per tumor type for ctDNA and *n* = 2 for rhodamine). Forty-eight hours after injection, tumors and organs (including the liver, spleen, lung, kidney, colon, and pancreas) were extracted and subjected to examination using confocal microscopy on frozen sections. A strong rhodamine signal was detected in the tumors of mice injected with rhodamine-labeled ctDNA when compared to control mice (Figure 1E). Importantly, no immunofluorescence signal was identified in any of the organs studied, implying that ctDNA accumulates primarily in tumor cells (Supplementary Figure S1E).

### ctDNA is predominantly incorporated into cells from the same cell of origin

**Figure 2.**
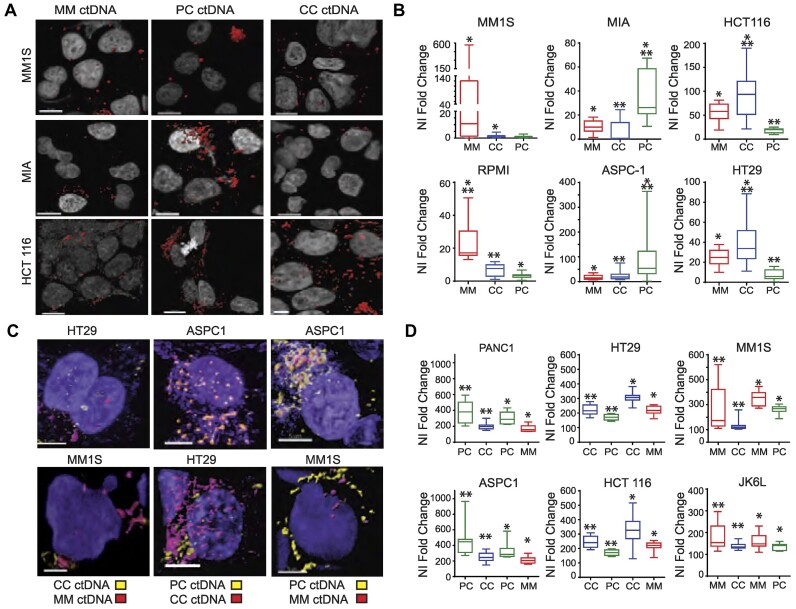
ctDNA cell-specific targeting. (**A**) Index images of 10 experiments and B. fold change of ctDNA nuclear density measurements in cell lines cultured with ctDNA matching or not matching the patient's cancer type. (**B**) whiskers plot summarizing fold change of nuclear density measurements of cells in culture with ctDNA derived from cancer patients matching or not the tumor type of the cell line (*n* = 10 experiments. *,** *P*< 0.05) C. Index images and (**D**) fold change of nuclear density measurements of simultaneous cocultured of tumor matched and unmatched ctDNA and cell lines (*n* = 10 experiments). MM: multiple myeloma, CC: colon cancer; PC: pancreatic cancer. MM cells: MM1S, RPMI, JK6L, PC cells: ASPC-1, PANC1, MIA and CC cells: HT29 and HCT 116. Error bars in the box and whiskers plot identified the standard deviation of triplicate experiments.

The discovery that ctDNA preferentially targets tumor cells suggests that ctDNA has a selective tropism towards cells identical to those from which it originated. We examined this possibility by coculturing ctDNA with tumor cell lines from tissues other than the patient's ctDNA origin tissue. When ctDNAs from MM, PC or CC patients were placed in culture with tumor cells of a different type, the ctDNA formed clusters at the outer edge of the cell membrane and did not enter the cells. In contrast, when ctDNA was cocultured with cells of the same tumor type as ctDNA, nuclear internalization was dramatically increased (Figure 2A and B). To corroborate this unexpected finding, we performed a competitive assay by simultaneously adding ctDNA that matched or did not match the tumor type of the cell line and assessed the ctDNA’s nuclear localization. ctDNA uptake was higher in cells that matched the tumor type of ctDNA (Figure 2C and D). On the other hand, under settings where ctDNA:cells did not match, the internalization of ctDNA was reduced. 3D reconstruction of the images allowed us to identify that in the rare situations where mismatched ctDNA entered the nucleus, and it colocalized with matching ctDNA (Supplementary Video S1A and B).

We subsequently probed the cell-type-specific tropism of ctDNA within an animal model hosting dual xenografts representative of MM and PC. Rhodamine-MM and CY5-PC-labeled ctDNA were mixed and injected simultaneously into each animal's tail veins (*n* = 2). Microscopic examination of the tumors revealed a preferential localization of rhodamine-labeled MM ctDNA within MM xenografts as opposed to PC xenografts. Conversely, CY5-labeled PC ctDNA accumulated in the PC xenograft with no significant accumulation in the MM xenografts (Supplementary Figure S1F). Overall, these results indicate that ctDNA has a special affinity for cancer cell types that correspond to their cell of origin.

### Chromosomal integration of ctDNA

**Figure 3.**
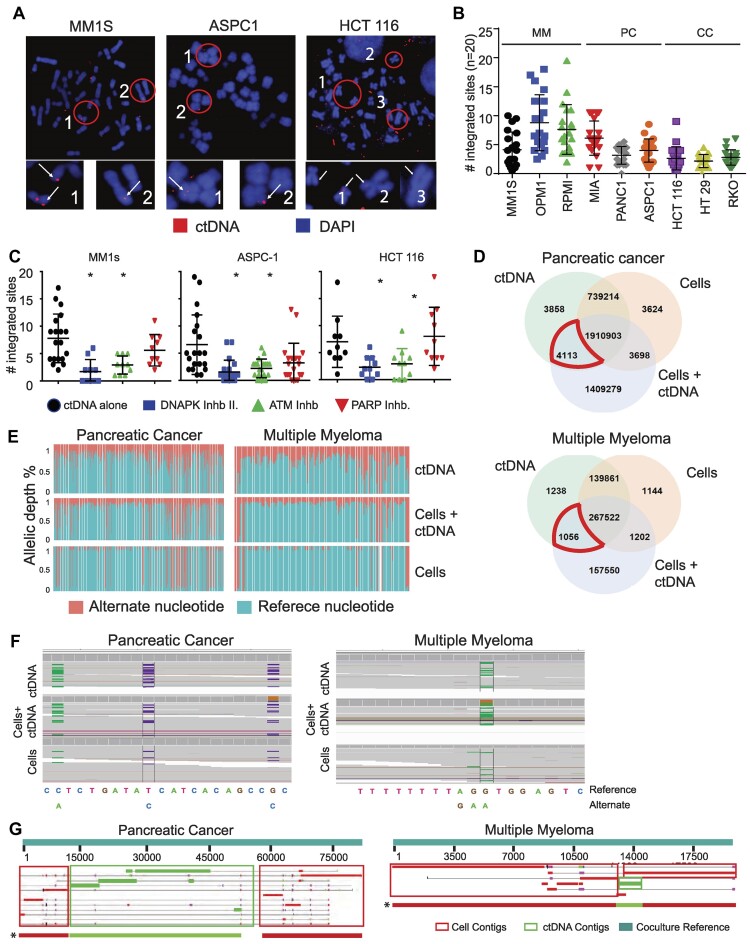
Integration of ctDNA is mediated by non-homologous end joining (NHEJ) repair. (**A**) Immunofluorescence index images of ctDNA (red) integration into chromatids (blue) in MM (MM1S), PC (ASPC-1) and CC (HCT116) cell lines. Circles define zoomed regions of interest. White arrows identify an area of ctDNA integration. (**B**) Scatter plot displaying the number of chromatids with ctDNA integrations in different MM (MM1s, RPMI, OPM1), PC (MIA, PANC1, ASPC-1) and CC (HCT 116, HT29, RKO) cell lines. Error bars indicate standard deviations of triplicate experiments (*n* = 20 metaphases per experiment). Color and symbol shapes signify different cell types. (**C**) Incorporation of rhodamine-ctDNA fragments into chromosomes of MM (MM1s), PC (ASPC-1) and CC (HCT 116) cell lines after treatment with NHEJ (DNAPKcs and ATM) and PARP (*n* = 10) inhibitors. Cells were pretreated for 2 h with inhibitors of DNA-PK (inhibitor II, 30 nM), ATM (KU-55933, 100 nM) and PARP (NU1025, 200 nM) before the addition of rhodamine-ctDNA to the cell culture. The number of integrated sites was measured in 10–20 metaphases per condition. (**D**) Gain of nucleotide variants in cells cocultured with ctDNA. Comparative SNV analysis between cell genome, ctDNA, and coculture. The Venn diagram displays exclusive and shared SNVs for each experimental condition. The area marked in red highlights SNVs commonly shared between ctDNA and coculture condition ctDNA/cell. (**E**) Stacked bar diagram demonstrating the changes in allele depth of the variant (red) and reference (Blue) allele in MM ctDNA, cell line genome, and coculture condition. Cells under coculture conditions have more depth in the variant allele in several locations compared to the control cell genome. (**F**) Index IVG variant calls images and their allele frequency in MM and PC experimental conditions. Green horizontal bars (Adenine) and purple (Cytosine) are, in these cases, the alternate nucleotide, and gray bars are the reference nucleotide. (**G**) Blast images demonstrate the transition point of insertion between cell genome contigs (red boxes) and ctDNA contigs (green boxes). (*) The cumulative of all reads, including the insertion transition site between cell genome contigs (red boxes) and ctDNA contigs (green boxes), is displayed at the bottom. Results were obtained after comparing the contigs carrying insertions in the coculture condition with the reference Coculture (ctDNA + cell) genome. MM: multiple myeloma; PC: pancreatic cancer; CC: colon Cancer.

To see if ctDNA fragments may integrate into the cell genome after entering the cell, we conducted metaphase chromosomal spreads on various cell lines that had been cocultured with ctDNA for 24 h (*N* = 3 per tumor type). The results demonstrated an elevated number of chromatids exhibiting rhodamine-ctDNA bands (Figure 3A and Supplementary Figure S2A). The presence of ctDNA-integrated chromatids appears to vary across different cell lines, as seen in Figure 3B.

The capacity of ctDNA to integrate into the genome was further determined utilizing DNA repair inhibitors. Myeloma (MM1s), pancreas (ASPC-1) and colon (HCT 116) cancer cells were treated for 2 h with DNA damage response inhibitors (i.e. ATM inhibitor (KU-55933), DNA-dependent protein kinase catalytic subunit inhibitor (DNA-PK inhibitor II), and an alternative join repair inhibitor (PARP, NU1025)]. Following treatment with these inhibitors, cells were incubated with rhodamine-ctDNA for 24 h and assessed by chromosome spreads the impact of each inhibitor on ctDNA chromatid integration. When compared to untreated cells, inhibition of ATM and DNA-PKcs dramatically reduced ctDNA integration into the genome. PARP inhibition, on the other hand, had minimal effect on ctDNA incorporation in most cells (Figure 3C and Supplementary Figure S2B). Given the potential influence of these inhibitors on cell viability and their potential side effect on the integration of ctDNA, we demonstrated that the concentrations used of these inhibitors did not affect cell viability. Hence, the observed results indicate that the decrease in ctDNA integration on the chromatids is a realistic consequence of these inhibitors (Supplementary Figure S2C).

These functional assays prompted us to verify the integration of ctDNA sequences into the cellular genome by whole genome sequencing of three distinct groups of samples: (i) the MM1s (MM) and MIA (PC) cell lines, (ii) ctDNA collected from the patients as delineated in Figure 3B and (iii) cell lines that were incubated for 24 h with aliquots of the aforementioned ctDNA. Cells were treated with DNase I for 10 min before DNA extraction to avoid false positive results produced from ctDNA contamination in the media. First, we tested whether cells gained single nucleotide variations (SNVs) exclusively donated by the ctDNA in the coculture condition ctDNA. The sequences from each experimental condition were first compared to the reference human genome to identify nucleotide variations (Hg38). Then, a three-way comparison of the three experimental conditions revealed multiple SNVs shared by coculture conditions and ctDNA that were not found in control cells' genomes (here called ‘SNVs of interest’, Figure 3D). The gain of SNVs of interest revealed a shift in the variant ctDNA allele frequency in cells cocultured with ctDNA compared to cells alone (Figure 3E). Figure 3F and Supplementary Figure S3A show other examples of SNVs with ctDNA ‘skewing’ in the variable allele frequency (VAF) following coculture with ctDNA.

Subsequently, we utilized the sequence data obtained from both tumor models to reconstruct genomic contigs for each experimental condition by de novo assembly. To map ctDNA genomic integration points more precisely and prevent Illumina library preparation insertion artifacts, MM and PC ctDNA samples were ligated with a PACBIO probe to identify their 5′ and 3′ ends prior to library preparation. The sequencing output of ctDNA, cell lines, and coculture conditions was then used to do de novo assembly. Comparison analysis of the contig sequences from these three experimental conditions identified contigs carrying ctDNA insertions. We confirmed the ctDNA origin of several of these insertions in coculture conditions by looking for SNVs present only in ctDNA samples. Next, we selected contigs containing clear transition points between the cellular genome. These selected contigs were then aligned with the ctDNA contigs containing the PACBIO adaptor. The quantification of structural variants (SV) in cells exposed to ctDNA compared to reference control cells revealed a gain of 1478 and 4440 events in MM and PC, respectively (Supplementary Figure S3B). Several of the SV, as well as the transition sites caused by the insertion of ctDNA fragments under coculture conditions, were validated by blast analysis (Figure 3G and Supplementary Figure S4A). Thus, these orthogonal analytical approaches validated the introduction of ctDNA fragments into the cell genome under coculture conditions. Analysis of ctDNA integrants and genome-insertion sites identified in both tumor models indicated that most (∼67%) of the inserted ctDNA fragments originated from chromosomes 3 and 7. Moreover, ∼80% of the ctDNA fragments targeted cellular chromosomal regions near the genomic location from which ctDNA originated (Supplementary Spreadsheet S1A-B and Supplementary Figure S4B–D). The remaining 20% were inserted into a chromosomal location distinct from their site of origin.

Given the observation that ctDNA tends to integrate close to homologous genomic regions, we look for alterations in genomic duplications. Specifically, we identified duplication events of ≥10 bp in length that occurred uniquely under coculture conditions but were absent in control samples (cells cultured alone, Supplementary Figure S4B–D). Subsequent selection focused on duplications within contigs containing SNV in coculture conditions that were analogous to those identified in the ctDNA, thus validating the ctDNA as the source. Comparative analysis revealed a significant increase in duplication sites, totaling 5018 and 6435, when MM or PC cells were cocultured with ctDNA corresponding to their tumor type. This substantial increase confirms the integration and genomic amplification of ctDNA within the cellular genome under coculture conditions.

Lastly, we assessed whether the integration of ctDNA fragments led to the enrichment of specific biological pathways. Gene ontology analysis of the tissue-specific inserted segments failed to achieve statistical significance due to the minimal number of insertions (Supplementary Figure S5). Nonetheless, distinct trends were observed in the pathways augmented by MM and PC ctDNA inserted fragments. Among the enriched pathways in MM were the phosphatidylinositol metabolic process, microtubule and calmodulin binding, double-stranded RNA binding, and mitochondrial protein-containing complex. Cell junction assembly, cell morphogenesis regulation, cell cortex, histone deacetylase complex, and protein tyrosine phosphatase activity, among other pathways, were shown to be enriched in PC-inserted pieces (Supplementary Figure S5). Furthermore, an evaluation of enrichment of oncogenes revealed that in the MM cocultures, there was a predominance of non-annotated sequences, followed by MAST2, MMCC1, and PLCD4. Similarly, in pancreatic cancer coculture, most of the genetic material was non-annotated followed by AIG1, MCF2L2 and PRIM2 among others (Supplementary Table S1A and B).

### Transposon sequences mediate cell targeting of ctDNA into specific tumor cells

To identify elements involved in mediating ctDNA cell targeting and integration, we designed an analytical approach to identify the presence of transposons sequences in ctDNA fragments inserted in the cell while reducing false positive events produced by Illumina library preparation. The contigs with ctDNA insertions identified above were chosen for this analysis. This analysis used the RepeatMaster (http://www.repeatmasker.org,(25)) program to identify and categorize the transposable elements present in ctDNA sequences. Only transposon sequences located within a 100-nucleotide range from the point where the cell transitions to ctDNA, and next to the PACBIO region, were selected for analysis. Our approach detected many transposable element sequences in close proximity to transition points in ctDNA fragments that had been integrated into the cell genome. Importantly, ctDNA integrants contained more transposon sequences than non-integrated ctDNA fragments (Supplementary Table S2).

**Figure 4.**
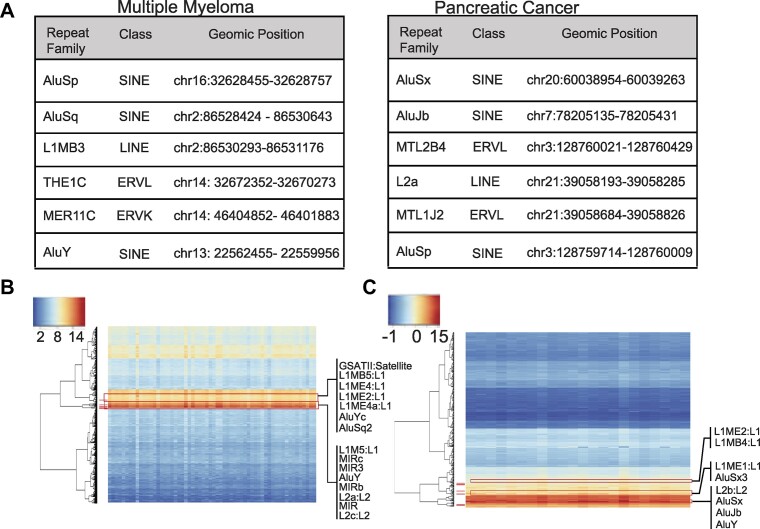
Transposon-mediated gene transfer of ctDNA in cancer cells. (**A**) Tables summarizing the list of tissue-specific transposons identified at the ctDNA insertion sites. (**B**, **C**) Expression levels of selected TE in PC and MM tumor samples. MM: multiple myeloma; PC: pancreatic cancer; CC: colon cancer.

In the next phase of the analysis, we looked for transposon sequences that preferentially target cells from the same tumor type. By examining the previously identified transposons, we identified specific transposable elements in cocultures with matched tumor types (e.g. MM ctDNA with MM cells, and PC ctDNA with PC cells) compared to mismatched conditions. We then selected transposons that were exclusively inserted in matching coculture conditions for each tumor type. Interestingly, ERV-Ls, LTRs, SINEs, and LINEs, accounted for the majority of transposable elements sequences at the transition locations in MM or PC. AluSx, MIRc, and MTL1J were among the most common transposon sequences found at insertion locations in matching PC ctDNA. AluSp, MER11, MER11C, AluJb and L2a, among other transposon DNA sequences, were found at MM ctDNA insertion sites (Figure 4A and Supplementary Table S3).

Next, we began assessing the factors that may contribute to the involvement of transposon DNA sequences in ctDNA insertion. It has been demonstrated that cancer cells activate short and long interspersed nuclear elements (SINEs and LINEs), and that the expression of LINE-1 (L1) aids in the transposition of additional repetitive elements. Accordingly, we examined the expression of transposable elements in cancer cells from 60 MM patients and 23 PC patients. To that purpose, raw data from batch normalized RNA sequencing was employed to assess expression variability (methods section). The study revealed the presence of many retrotransposons, including L1, in MM and PC samples (Figure 4B and C and Supplementary Table S4), prompting us to investigate the role of reverse transcriptase inhibitors in ctDNA chromatid integration. Multiple myeloma (MM1s), pancreatic cancer (MIA), and colon cancer (HCT116) cell lines were treated for 24 h with the reverse-transcriptase inhibitors zidovudine (AZT) or didanosine (DDI), before adding the ctDNA to the culture media. Reverse transcriptase markedly reduced ctDNA chromatid integration compared to the vehicle-treatment control (Supplementary Figure S6A). These findings could indicate that the protein machinery encoded by an endogenous retrovirus or retrotransposon is involved in the incorporation of certain ctDNA fragments into the host cell genome.

**Figure 5.**
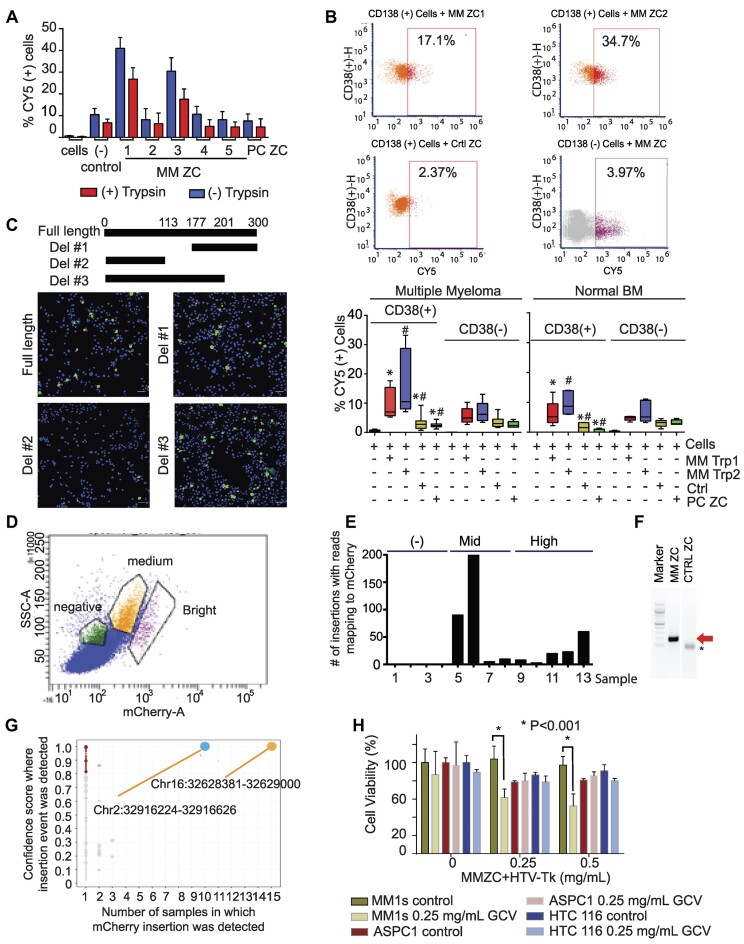
MM transposons mediated gene transfer to cancer cells. (**A**) MM1s cell capture an internalization of different MM retrotransposons and controls after 4 h of culture. (**B**) Index flow cytometry images displaying the cell capture of AluSp, MER11, PC transposon (AluSx) and control sequence in plasma cells and non-plasma cells derived from bone marrows of patients with newly diagnosed or treated MM patients (*n* = 12) and patients with various cancers other than MM (*n* = 5). The lower part of this figure displays box plots summarizing the number of CY5 positive cells measured after 14 h of culture of twelve MM and five non-myeloma bone marrows. (**C**) Effect in MM1s cell internalization of Cy5 labeled AluSp and its deletion mutants. Images were captured after culturing cells with the retrotransposons for 8 h. (**D**) flow cytometry image displaying different degrees of mCherry expression in cells cultured for 24 h with AluSp-CMV-mCherry cassette. (**E**) Bar chart displaying the summary of the number of insertions identified in mCherry expressing (+) and non-expressing (-) cells. (**F**) Validation of the mCherry integration by PCR from chromatin extracted from cells treated with AluSp-CMV-mCherry cassette or control-CMV-mCherry cassette. *nonspecific band. (**G**) Graph displays the confidence of detection of CMV-mCherry insertions identified versus the frequency of each specific site of insertion. (**H**) Cell survival of 3 different cell lines cocultured with TE-HSV-Tk-GFP for 24 h prior to adding ganciclovir (GCV). Apoptosis was measured at 96 h after GVC addition. MM: multiple myeloma; CC: colon cancer; PC: pancreatic cancer; TE-CMV-GFP: transposon element joining to CMV-GFP; HSV-TK: herpes simplex virus thymidine kinase. Error bars in the box and whiskers plot identified the standard deviation of triplicate experiments.

To validate if the transposon sequences identified above can transfer genetic material between cancer cells, we chemically synthesized several MM transposon DNA sequences and tested their GT capacity. From among the list of transposon sequences, we selected those that can be synthetized via the gBlock gene fragment method (AluSp, AluSg2, MER11C, THE1A and AluSx, Supplementary Table S5). Prior to the cell line experiments, the sequences were evaluated for their resistance to degradation by DNAses present in complete culture media. A synthetic DNA sequence with similar length and GC content that does not encode a transposon and a PC transposon sequence (AluSx1) were used as controls. Agarose gel electrophoresis demonstrated that all fragments remain intact under culture conditions (Supplementary Figure S6B). The transposon DNA sequences were then labeled with CY5 and evaluated for their capacity to target and internalize into MM cell lines. First, we evaluated the kinetics of transposon capture by flow cytometry. Our results showed that cell capture of CY5-AluSp sequence increases over time more efficiently than the capture of the control sequence (Supplementary Figure S6C). Subsequently, we performed dose titration experiments to evaluate cell capture and internalization. Internalization of the DNA was considered if Cy5-DNA was not removed from the cells after trypsin treatment. Cell capture and internalization CY5-AluSp were dose-dependent and more efficient than the capture of the control sequence (Supplementary Figure S6D). Based on these findings, we compared the efficiency of cell capture of all transposon DNA sequences in two MM cell lines at 4 h of culture. CY5-AluSp and CY5-MER11C were captured more efficiently than the other MM transposons, the control sequence, or PC transposons (Figure 5A and Supplementary Figure S6E).

The cell targeting of AluSp and MER11C DNA sequences was then measured in bone marrow samples from patients with MM or without MM but with other bone marrow disorders. After 14 h of culture with marrows derived from myeloma patients, we observed more CY5-positive plasma cells (CD 38 [+]) in marrows cultured with CY5-AluSp or -MER11C than with control or PC sequences (Figure 5B). In contrast, AluSp or MER11C did not exhibit a difference in CD38 (–) cell capture compared to control or PC sequences. As further evidence of their ability to specifically target malignant plasma cells, we found that AluSp and MER11C-CY5 capture was more efficient in CD38 (+) cells than in CD38 (–) cells (AluSp CD38 [+]: 9.9% 1.6% versus CD38 [-]: 5.2% 0.8% *P*= 0.03 and MER11C: CD38 [+]: 16.50% 3.8 versus CD38 [–]: 7% 1.2% *P*= 0. 032). However, there was no distinction between CD38 (+) and CD38 (–) cells in control or PC sequence cell capture. Experiments with the bone marrow of non-myeloma patients demonstrated a tendency of greater CD38 (+) cell capture by AluSp and MER11C compared to controls or PC transposon sequences, with the trend disappearing in CD38 (–) cells. Lastly, when we compare the uptake of malignant CD38 (+) cells to non-malignant CD38 (+) cells, we see a non-significant increase in the AluSp and MER11C uptake of malignant CD38(+) cells.

To test whether the transposon sequence contains a cell recognition signal, we generated several deletion mutants of the AluSp sequence and evaluated their impact on cell capture. As shown in Figure 5C and Supplementary Figure S6F, deleting the central region of the AluSp reduced MM cell capture, suggesting that these ∼80 bp region contain a MM cell recognition sequence. In fact, MM1s and JK6L cells showed similar cellular uptake for the entire length of AluSp and the 80 bp. This 80-nucleotide sequence has two evenly enriched GC and AT regions (Supplementary Figures S6G). Surprisingly, replacing GC with AT in the two GC enrich regions increased 80-nucleotide cell capture compared to wild type-AluSP or –80 bp recognition sequence. In contrast, when we substitute GC for CG in this GC enrich region, we reduce MM1 and JK6L cell capture, implying that there are sequence-specific factors that influence cell interaction (Supplementary Figure S6H and I).

### Chemically synthesized MM transposon sequences imitate ctDNA’s horizontal gene transfer capabilities

Having determined that AluSp was able to target MM cells specifically, we evaluated whether it could deliver other genetic material into cells. For this, we ligated AluSp sequences to both ends of a linearized CMV-mCherry cassette. These AluSp-mCherry ligates were then added to the culture media of MM1s cells for 24 h, after which mCherry expression was evaluated microscopically to test for successful transgene expression. Microscopy images demonstrate that mCherry expression was higher in AluSp-mCherry-treated cells than in cells incubated with the linearized vector or the transfected circular CMV-mCherry vector (Supplementary Figure S7A). Flow cytometry also detected different levels of mCherry expression in MM1s cells (Figure 5D). To determine whether the mCherry cassette integrated into the MM1s genome, we sequenced the genomes of single cells expressing different levels of mCherry. mCherry insertions were identified by recognizing sequences with one read aligned to the cell genome while the mate aligned to the CMV-mCherry sequence. This analysis detected various mCherry insertions in cells with mid or high expression levels, while no insertions were identified in cells without mCherry expression (Figure 5E). In contrast, when cells were cultured with the control-mCherry sequence, only a few were found with high levels of mCherry insertion and expression. mCherry integration was confirmed by mCherry PCR of cells cultured with AluSp-mCherry vector but not control-mCherry cultured cells (Figure 5F). The AluSp-CMV-mCherry vector integrated high confidence into two specific regions in the genome (Chr2:32916224–32916626 and Ch16:32628381–32629000), which are enriched for simple repeats and AluSp (Figure 5G). We then determine if an intermediate RNA transcript is required prior to insertion. An ALuSp-CMV-mCherry linearized DNA sequence was synthesized and incubated with MM cell lines, after which the presence of mCherry transcript, AlusSp, and mCherry genomic DNA integration was evaluated at different time intervals. These results show that the AluSp-mCherry sequence was integrated before to RNA synthesis, implying that DNA integration occurs independently of an RNA intermediary (Supplementary Figure S7B).

Following the demonstration that the AluSp DNA sequence can deliver a gene, we investigated whether this property can be exploited therapeutically. We ligated a herpes simplex virus thymidine kinase (HSV-Tk) killer gene between AluSp sequences (AluSp-HSV-Tk-GFP) and tested this vector's ability to kill MM, PC, and CC cell lines. Cells were cultured for 24 h with the AluSp-HSV-Tk-GFP and then cultured for 96 h in ganciclovir (GCV). At that point, we measured the effect on cell numbers of the biologically active byproduct of GCV produced by the action of HSV-Tk. As shown in Figure 5H, GCV markedly reduced the viability of MM cells previously treated with MM-AluSp-HSV-Tk. In contrast, AluSp-HSV-Tk/GCV treatment did not affect the viability of PC or CC cell lines. These results demonstrated that the synthetic AluSp could transfer and integrate genetic cargo into specific cells (Figure 5H).

### ctDNA alters the drug response of MM or PC cell lines

**Figure 6.**
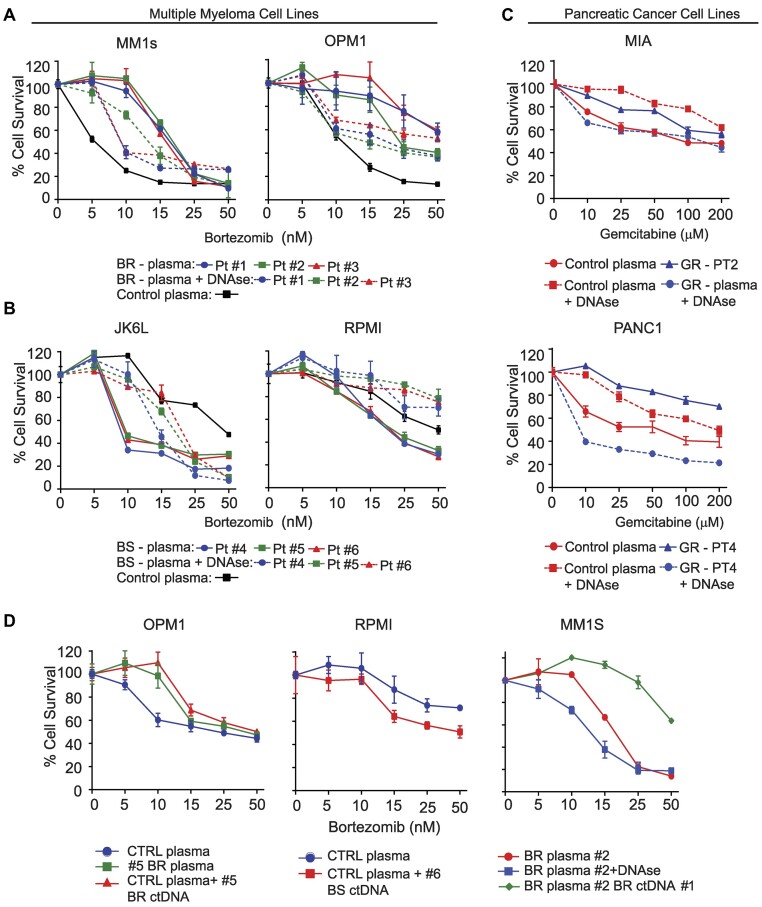
ctDNA-mediated transmission alters the sensitivity of the cells to bortezomib or gemcitabine. (**A**) Cell viability assay measuring sensitivity to bortezomib in MM cell lines OPM1 and MM1s cells cultured with FBS or DNAse I treated or non-treated plasma from MM patients that failed to respond to bortezomib. (**B**) Cell viability assay measuring sensitivity to bortezomib in RPMI and JK6L cells cultured with FBS or DNase I-treated or non-treated plasma from a MM patient that responded to bortezomib-based treatment. (**C**) Cell viability assay measuring sensitivity to gemcitabine in PC cell lines (MIA and ASPC-1) cultured with plasma from patients resistant to gemcitabine, similar plasma pretreated with DNAse I or a control non-cancer patient. For the corresponding DNAse I-treated samples, plasma was treated with DNase I for 10 min. (**D**) (Left), Comparison of cell viability response to bortezomib in OPM1 cells cultured with plasma from a patient that failed to respond to bortezomib treatment, the combination of plasma derived from a patient without cancer with ctDNA derived from the same patient resistant to bortezomib and control plasma alone (non-cancer patient). (Middle). Viability response of RPMI to control plasma or control plasma with added ctDNA obtained from a patient that achieved a complete response to bortezomib. (Right) Cell viability assessment after bortezomib treatment of MM1s cells cultured with plasma from a bortezomib-resistant patient (BR#2) alone or after treatment with DNAse I or coculture with ctDNA from a different bortezomib-resistant (BR#1) patient. MM: multiple myeloma; PC: pancreatic cancer; BR: bortezomib resistant; BS: bortezomib sensitive; GR: gemcitabine resistant. Error bars indicate the standard deviation of triplicate experiments.

Having demonstrated GT between cancer cells, we explored the potential consequences of GT on the phenotype of target cells, particularly on the target cell's response to drugs. We, therefore, cultured Bortezomib-sensitive (BS) MM cell lines (MM1s and OPM1) for 24 h with DNase-treated or non-treated serum from bortezomib-resistant (BR) patients or control serum from non-cancer patients. Subsequently, increasing doses of bortezomib were added to culture media, and cell survival was measured 24 h later. Plasma from patients resistant to bortezomib increased the bortezomib resistance of MM1s and OPM1 compared to FBS (Figure 6A-C). This effect of BR plasma was eliminated by pretreating with DNase (Figure 6A). When BR MM cell lines (JK6L and RPMI) were cultured with a plasma of patients sensitive to bortezomib, the cells exhibited a significant restoration of bortezomib sensitivity compared to when the cell lines were cultured with FBS. DNAse treatment of BS plasma abolished this effect to levels similar to control plasma (Figure 6B). Similar experiments were performed with PC cell lines (MIA Paca-2 [MIA] and PANC1) using DNase-treated gemcitabine (GEM)-resistant (GR) and control plasma. Both cell lines became resistant to GEM when cultured with GR plasma but not with control plasma. DNase treatment of the GR plasma abrogated the effect (Figure 6C). In contrast, DNase treatment of control plasma resulted in increased resistance of PC cells to gemcitabine (Figure 6C, lower graphs). Furthermore, the effect of the patient's plasma on drug response persisted beyond 24 and 48 h after condition media was replaced with regular media (Supplementary Figure S8A, B).

To further characterize the contribution of ctDNA transferring drug response, we added cell-free ctDNA from bortezomib-resistant or -sensitive patients to media containing plasma of a patient without cancer or BR. The plasma was then incubated with MM1 or OPM1 cells. Both cell types lost sensitivity to bortezomib (Figure 6D). Resistance to bortezomib was increased even further when the cells were incubated with the plasma of a BR patient to which BR patient ctDNA had been added (Figure 6D). In contrast, adding a BS patient's ctDNA to RPMI cells cultivated with control plasma from a non-cancer patient boosted sensitivity to bortezomib (Figure 6D). Treatment of ctDNA with proteinases had no apparent effect on its effects on drug response (Supplementary Figure S8C). These findings suggest that ctDNA transmits genes that confer drug sensitivity or resistance from one cell to another.

## Discussion

Transmission of TEs plays a significant role in the evolution of prokaryotes and some eukaryotes. Yet, the implications of GT in human cancer are not well characterized. The current study demonstrates that naked ctDNA can serve as a vehicle for transferring genes between cancer cells. These findings define fundamental properties of ctDNA-mediated GT and identify a previously unrecognized characteristic of ctDNA: its inherent affinity for recipient cells that are phenotypically and genotypically similar to the donor cells. The identification of TE DNA sequences at ctDNA insertion sites and the results showing that synthetically-generated transposon DNA sequences can deliver payloads to target cells support the notion that these sequences have an essential role in mediating GT in cancer. Moreover, experiments utilizing deletion mutants and point mutations delineated regions within the transposon sequences that specify the delivery of ctDNA to a particular target cell. Importantly, these results pave the way for future research into the use of a synthetic Transposon DNA sequence-mediated Gene transfer (TGet) to precisely transfer ‘cargo’ to specific cells.

Although there is evidence in humans of nucleic acid transfer through exosomes and apoptotic bodies (26), there is limited data available to suggest that some cells transfer information through the release of naked double-stranded DNA (14,27). Two groups have explored the role of ctDNA as a messenger of genetic material in cancer over the last decade (14,17,18). Garcia-Olmo and colleagues suggested that plasma from colon cancer patients is capable of transforming murine embryo fibroblast cell line (NIH-3T3). More importantly, this team showed that plasma from CC patients transmitted oncogenes such as K-Ras to NIH-3T3 cells (18,28). These findings were later supported by Trejo-Becerril and colleagues, who observed the transfer of the human K-Ras oncogene from SW480 xenograft to chemically-induced colon cancer tissue in rats (17). These results align with our studies demonstrating the transfer of genetic material from a patient's ctDNA to cancer cell lines. We showed in our sequencing analysis that ctDNA integrates into host cells, a ctDNA-CMV-GFP cassette enables GFP expression in MM cells, and a synthetic transposon derived from ctDNA can cause mCherry integration. This finding provides evidence that ctDNA functions as a vehicle for gene transfer and is consistent with the hypothesis that ctDNA can play a role in altering the genetic architecture of the tumor mass. More importantly, these observations demonstrating the tissue-specific tropism of ctDNA provide a potential mechanistic explanation for the studies of Trejo-Becerril (17), which showed the transfer of mutated human K-RAS from xenografts to chemically induced colonic tumors. However, the current data falls short of identifying the mechanisms by which ctDNA is recognized and internalized by the cells. Hence, further work elucidating the cellular mechanism for ctDNA recognition will be essential for developing inhibitors to prevent the transmission of genetic material between cancer cells as a means of spreading drug resistance.

The implications of gene transfer in oncology are not well defined. This investigation has revealed the somewhat surprising finding that ctDNA derived from patients can alter the drug response phenotype of cell lines. The possibility of transmitting cancer through blood transfusion is a controversial subject of investigation. A large study conducted in Scandinavia with 888 843 cancer-free transfusion recipients reported a higher than expected rate of cancer in the years following the transfusion (29,30). This finding suggests that certain blood units harbor factors that could precipitate the development of occult cancers. Similarly, other studies have demonstrated an increase in cancer incidence after transfusion; however, the specificity of particular cancer remains under debate (31). While some studies indicate that transfusions may exert immunosuppressive effects and introduce cytokines that can cause carcinogenesis (32), the potential role of ctDNA in transfused blood modulating cancer risk warrants further investigation.

The evidence that mobile elements mediate GT in bacteria, flies, plants and eukaryotes (26) suggests the possibility that a similar mechanism could occur between cancer cells (33). The present investigation reveals several lines of evidence substantiating the role of transposon DNA sequences in the tissue-specific GT of ctDNA: First, comparative genomic analyses identified a marked enrichment of transposon sequences at tissue-specific insertion sites. Second, we showed that synthetic MM transposon sequences were captured more by MM cell lines and patient-derived plasma cells than by the non-plasma cell compartment. Third, the data show that MM transposon sequences can recapitulate the GT capacity of ctDNA, since they were able to deliver and integrate mCherry and HSV-TK genes into MM cell lines. Despite these advances, further work is required to elucidate the steps required for ctDNA uptake by host cells.

To address the challenge posed by potential retrotransposon-mediated integrants induced by Illumina sequencing, similar to those described in Drosophila (34), we designed an approach to minimize the impact of these artifacts on the findings. First, we ligated a single PACBIO sequence to ctDNA prior to the preparation of the Illumina library. This PACBIO sequence was essential for identifying the 5′ and 3′ end of the ctDNA fragments, thereby providing a reference for discriminating genuine insertions and transposons within these fragments during coculture and preventing us from selecting any false positive event. Second, we applied similar library preparation processes in all experimental conditions used in our analysis for insertion, enabling a three-way comparison of cell, ctDNA, and ctDNA coculture to filter out any artifact introduced during library preparation and shared between all samples. Third, to further reinforce the validity of our approach, we imposed a stringent criterion for detecting ctDNA insertions within the cellular genome by selecting only insertions encompassing SNV shared between coculture and ctDNA. Finally, transposons were chosen on the premise that they were tumor specific, meaning that were present when the tumor and ctDNA phenotypes corresponded and absent in non-corresponding instances.

This research has started to uncover that specific sequences within transposons function as recognition signals for cancer cells. This is comparable to the finding that in SLE, cell-free DNA is integrated into immune complexes and acts as an antigen recognition signal (35–37). Notably, treating ctDNA with double-stranded DNA antibodies derived from SLE patients reduced ctDNA uptake by MM and PC cells. Furthermore, this study documents that a synthetic AluSp DNA sequence can specifically target and convey genetic information into tumor cells. Alterations in the AluSp sequence, either through deletion of the 3′ end or point mutations in the middle 80 bp region, impaired the targeting ability. These findings indicate the presence of ‘zip code sequences’ within certain transposon DNA sequences that facilitate targeted delivery to cancer cells.

This research raises new questions relating to the mechanisms by which ctDNA is recognized, captured, and internalized. For example, several lines of evidence suggest the existence of a plasma membrane protein receptor for ctDNA, such as the specificity of ctDNA cell targeting and the high efficiency with which two of the MM transposon sequences target plasma cells while largely sparing other bone marrow cells. Also, treating the target cells with trypsin prior to culturing them with ctDNA reduces the number of ctDNA-positive cells. The putative receptor and the consensus ‘antigenic’ sequences in transposons and the mechanism by which ctDNA and the putative receptor interact need to be elucidated. Regarding the intracellular trafficking of ctDNA, our data indicate that the internalization of a ctDNA transposon sequence utilizes clathrin and endosomal maturation pathways similar to those used by aptamers (38,39). However, whether these pathways are universally applicable across various tumor types and for all transposon-associated ctDNA sequences remains to be determined.

The present study elucidates the mechanisms underlying the integration of ctDNA into the host genome, offering insights into the ectopic role of ctDNA as a template for DNA repair. Our findings revealed some of the steps implicated in ctDNA integration, highlighting (i) the involvement of ATM and DNAPK in the repair processes associated with ctDNA integration into the cellular genome; (ii) the proximity of transposable element sequences to the cellular integration sites, and the supporting evidence from experiments with a synthetic analog of one such transposon, underscore the importance of these elements in the ctDNA integration process and (iii) the observation that AluSp element-mediated integration can proceed independently of an intermediary RNA transcript (40–47). Despite these advances, further work is needed to elucidate the precise repair mechanisms by which ctDNA integrates into the cellular genome and how exactly components of L1 (40–45,48) contribute to the process of ctDNA integrations.

In conclusion, this study demonstrates the transfer of ctDNA, a process with the capacity to alter the phenotype of recipient cells. The underlying mechanism involves the secretion of the transposon and accompanying genomic DNA from donor cells into the extracellular space, culminating in reaching the bloodstream. Within the bloodstream, ctDNA can home to and enter target cells that typically share a tissue origin with the donor cell. The target cell displays a membrane receptor (likely a protein) that recognizes a specific address sequence in the transposon region of the ctDNA and mediates internalization of the ctDNA. Once internalized, the ctDNA travels to the nucleus and integrates into the host genome in a process facilitated by ATM and DNAPKc. This research represents a confirmed instance of ctDNA transfer between human cells but raises numerous questions, including the mechanism of ctDNA secretion, the nature of the specific receptor on the target cell, the mechanism of integration, the cGAS-cGAMP-STING host response (44), and other mechanistic features of the process. Furthermore, these discoveries introduce the possibility that genetic material may be exchanged between individuals, potentially through modalities such as blood transfusion. Finally, the elucidation of a method to selectively target certain tumor cells provides a potentially powerful and transformative tool for delivering therapeutic agents such as drugs, radioisotopes, nanoparticles, or cytotoxic genes into malignancies and other pathological conditions.

## Supplementary Material

gkae427_Supplemental_Files

## Data Availability

Sequencing data used for producing Figure 3C-D, 4B-C and Supplemental Figures 1A and 3A–C is available under Sequence Read Archive accession number: PRJNA1043708 and PRJNA1070019. For image processing and quantification of the ctDNA nuclear localization, the algorithms are available in Zenodo at https://doi.org/10.5281/zenodo.11111156.
