# Supplementary material for: Transposon DNA sequences facilitate the tissue-specific gene transfer of circulating tumor DNA between human cells
Source: Nucleic Acids Res. 2024 May 23;52(13):7539–55. doi: 10.1093/nar/gkae427 (PMC11260451; doi:10.1093/nar/gkae427)
Supplement: gkae427_Supplemental_Files [file gkae427_supplemental_files.zip › COMBINE SUPPLEMENTAL DATA 5-15-24 Final.pdf]

Supplemental Figure Legends

Supplemental Figure 1. A. Pie chart showing the single nucleotide variants (SNV) shared between tumors from patients and their corresponding ctDNA (orange) and SNV present only in the tumor sample (blue). B. Index pictures of rhodamine (control) or rhodamine-ctDNA nuclear capture utilized to quantitate ctDNA-nuclear quantification in Figure 1B. C. GFP expression in MM1s cells cocultured with linearized Cytomegalovirus-green fluorescent protein: CMV-GFP, ctDNA bound to CMV-GFP, and ctDNA-CMV-GFP. D-E. ctDNA localization in tumor xenograft and multiple organs in all ctDNA tail-injected mice. D. Confocal microscopy of the xenograft PC tumors harvested from mice after tail injection with rhodamine-PC ctDNA. Tumors were harvested at 24 and 48 hours post-injection. E. Images of different organs harvested from xenograft-mice tail injected with rhodamine-ctDNA (MM, CC, and PC) 48 hours after tail injection (n=3 per tumor xenograft). F. Images demonstrate the location of Rhodamine-MM and CY5-PC-labeled ctDNA in MM and PC tumors collected from xenograft mice. Each mouse harbors an MM and PC xenograft concurrently. A mixture of MM and PC ctDNA was simultaneously injected into the tail vein. Images were taken under the red channel to identify rhodamine fluorescence. MM: multiple myeloma, CC: colon cancer, PC: pancreatic cancer, and GFP: Green fluorescent protein.

Supplemental Figure 2. ctDNA integration into the cell genome. A-B. Visualization images display other examples of the gain of ctDNA integration into chromatids (red) of CC (HT29 and RKO) and PC (MIA and PANC1) cell lines. Circles define zoomed regions of interest. White arrows identify an area of ctDNA integration. B. Examples of the metaphase images of various cancer cell lines (MM1s, ASPC-1, and HT116) treated with ATM, DNAPKcs, PARP, and transposase inhibitors used to generate Figure 3C. Circles define zoomed regions of interest. White arrows identify an area of ctDNA integration. C. Cell viability assays of MM1, ASPC1, and HTC116 cells after 24 hours of treatment with 10 μM of ATM inhibitor (KU-55933), 30 μM of DNAPKcs Inhibitor II, 200 μM of PARP inhibitor (NU1025) or 100nM of raltegratvir. PC: Pancreatic cancer and CC: Colon cancer. MM1s: Myeloma cell line.

Supplemental Figure 3. A. Visualization images displaying other examples of the gain of a ctDNA SNV in the coculture experiment. B. Table summarizing the number of insertions/translocations in coculture cells when compared to the reference cell genome.

Supplemental Figure 4. A. Two examples of blast images that illustrate the transition point of a ctDNA insertion event in MM or PC coculture in comparison to the ctDNA contigs and the cell line. B-D. Blast images illustrate the alignment of ctDNA and cell contigs with the coculture reference contigs, as illustrated in Figure 3G. The transition point of insertion between cell genome contigs (red boxes) and ctDNA contigs (green boxes) are shown. C. Images exhibiting areas of homology at or near integration sites identified through blast analysis of cell contigs against ctDNA. D. Sequence of the areas of homology at or nearby integration sites identified by blast analysis of the cell contigs against the ctDNA. Coculture Contigs carrying and insertion were identified using NucDiff analysis. (*) The cumulative of all reads, including the insertion transition site between cell genome contigs (red boxes) and ctDNA contigs (green boxes), is displayed at the bottom.

Supplemental Figure 5. Gene ontology analysis demonstrates the processes and pathways enriched in the matching coculture conditions. Gene ontology processes include biological, molecular function, and cellular functions.

Supplemental Figure 6. A. Scatter plot displaying the number of chromatids with rhodamine-ctDNA integration of MM (MM1s), PC (ASPC-1), and CC (HCT 116) cell lines (n=30) after treatment with two reverse transcriptase inhibitors (0.4 μM AZT and 6 μMDDI) and an integrase inhibitor (100 nM raltegravir). B. PCR of the Control- or all transposons performed from DNA extracted after culturing these constructs with complete media for 4 hours. C. Time course of Cy5-AluSp and -control sequence treated MM1s cells (1 μg/mL). CY5(+) cells were detected by flow cytometry. D. Dose titration experiments of Cy5-AluSp and -control sequence treated MM1s cells. Before flow cytometry, half of the samples were treated with trypsin to identify how much DNA was internalized. E. flow cytometric screening of all transposons and controls in U266 cells after 4 hours in culture. F. PCR demonstrating the band of the different deletion mutants generated. G. Graphical display of the adenine (A)-thymine (T) and guanine(G)- cytosine (C) enrich regions or

both. H-I. Mutants of the 80 base pair recognition sequence were generated by substituting GC for AT or CG. Images and quantification of MM1s and JK6L cell capture of FMA-labeled double-stranded oligos were obtained after 4 hours of culture.

Supplemental Figure 7. A. Microscopy images of mCherry expression in MM1s cells after lipofectamine-mediated transfection with a circular CMV-mCherry vector compared to cells cultured with linearized CMV-mCherry vector or AluSp-CMV-mCherry without lipofectamine. B. Genomic DNA and RNA were isolated from MM1s cells grown with AluSp-CMV-mCherry DNA at various time points. DNA integration of the mCherry and AluSp, and mCherry RNA transcripts was determined by reverse transcription PCR (rtPCR) for mCherry and polymerase chain reaction (PCR) for AluSp and mCherry.

Supplemental Figure 8. A. Cell viability was measured in 2 myeloma cell lines (MM1s and OPM1) after culturing with BR plasma for 24 hours, followed by a washout period of 24 or 48 hours prior to bortezomib treatment. B. PANC-1 cell line was cultured with either GS or GR for 24 hours, followed by a washout period followed by treatment with Gemcitabine. C-D. Effect of proteinase treatment of the ctDNA on MM cells (MM1s and RPMI) response to bortezomib or PC cells (PANC-1 and MIA) on the response to Gemcitabine. PC: Pancreatic cancer and MM: Mulitple Myeloma.

Supplemental Figure 1

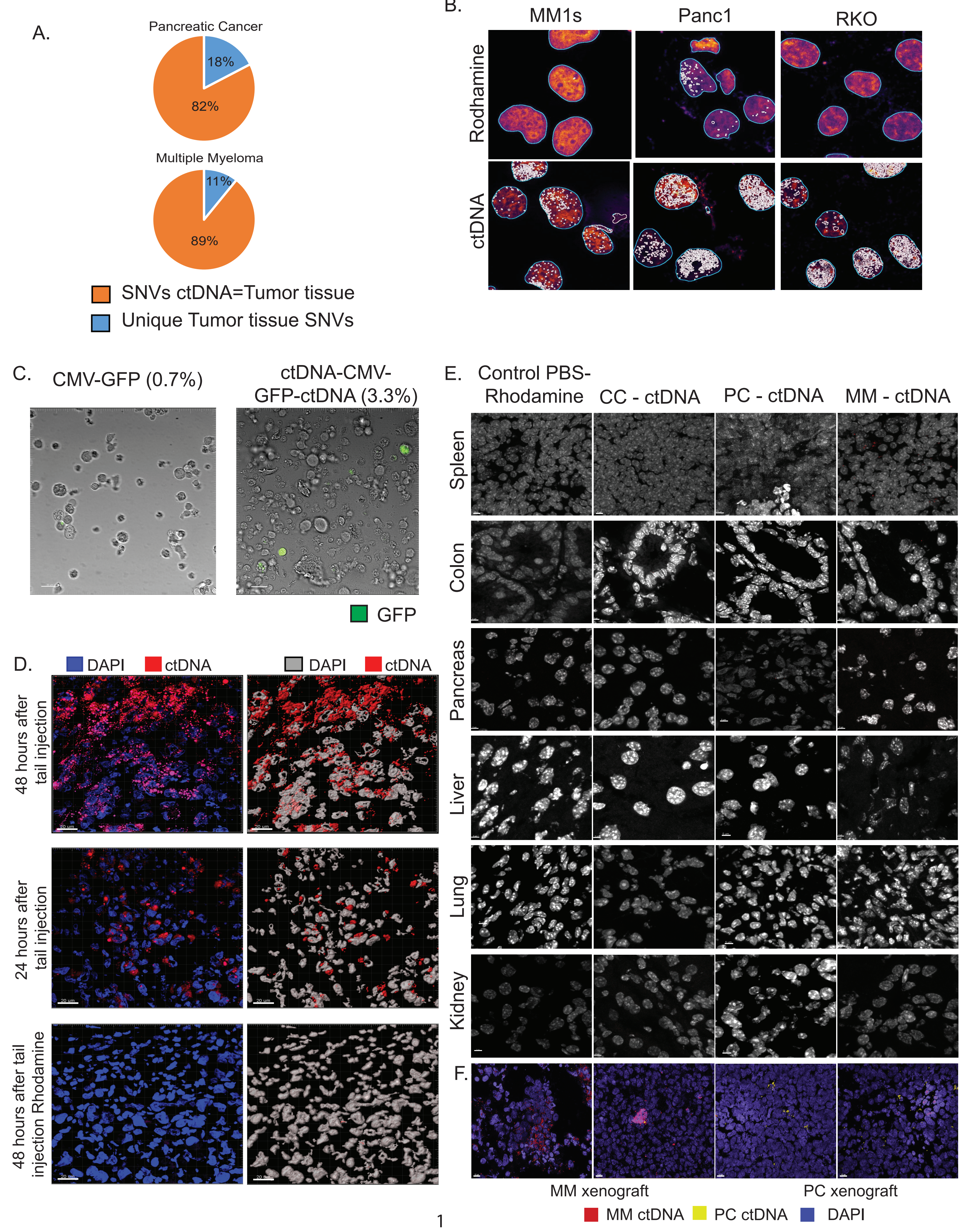

Supplemental Figure 2

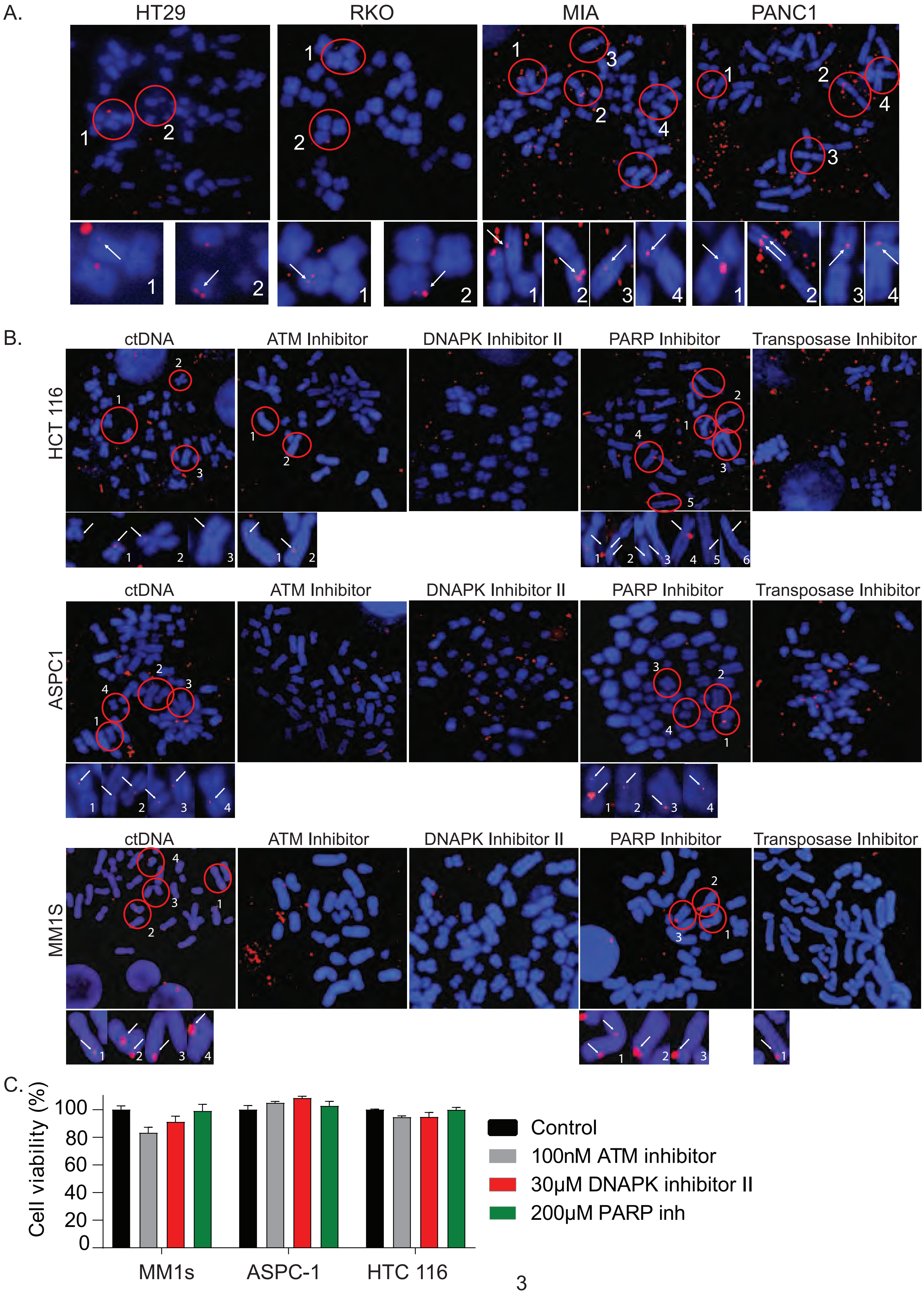

Supplemental Figure 3

A.

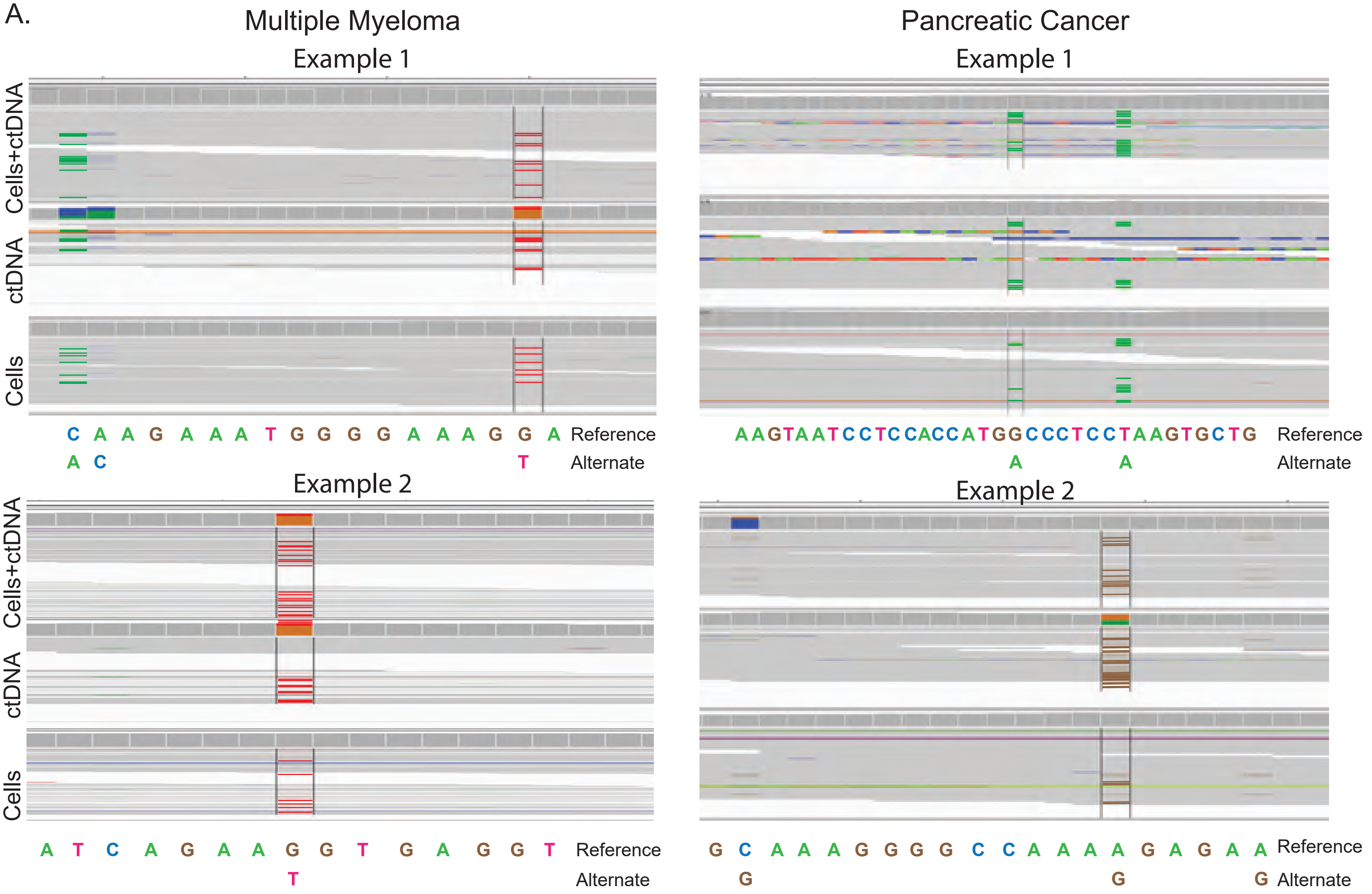

B.

	MM	PC
**Total insertion/translocations events >100bp**	15521	54380
**Insertion events within contigs that have a pacbio adaptor in ctdna referepce at beginning and end**	1478	4440

Supplemental Figure 4

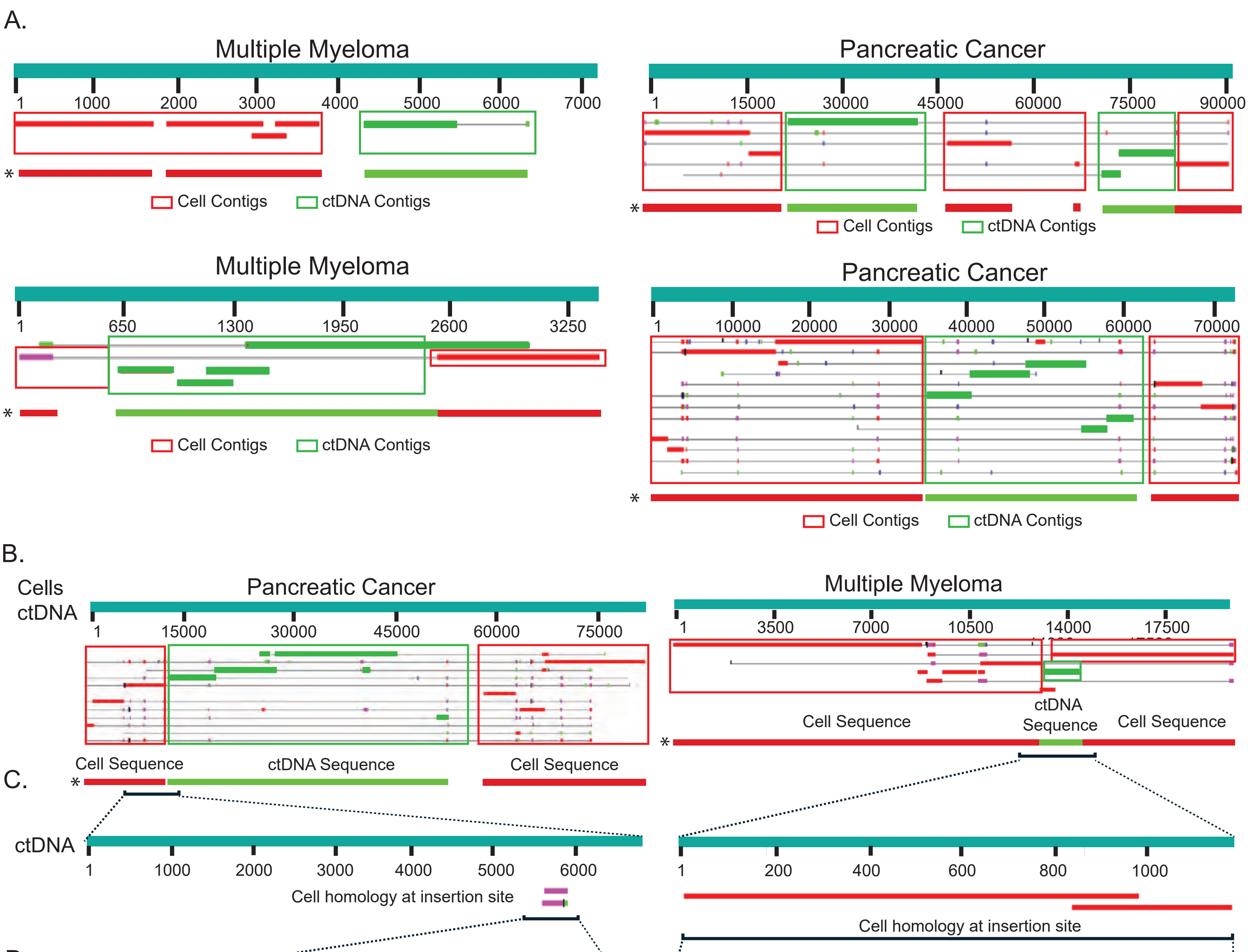

D.

GATGCAGTGGGTCATGTCTATAATCCAAGCTCTTTGGGAGACCCAGGCAGGTGGATAGGC
TGAGCCTGGGAGTTCGAGACCAGCCTGGCTGACATAGCAAAAGCCAGTCTCTAGCAAAAA
CAAACAAACAAACAAATTAGACATTTTTTGGTAGTCATCGCTTGTAGTTCCAGTTATTTGGG
AGGCTGAGGTGGGAGGATCACTTGAGCAGGGAAGTGGAGGTCGCAGGGAGAAGAGATT
GTGCCACTGCACTCCAACCTCTAGGGTGACAGAGTGAGACCTTGTCTCAAAAACAAACAA
ACAGAGCAAAAATTGGCTTGCAAGAGTGGATTCATTGATCTTTCACTTCTTCCATGTGAGG
AACAGTATTCCTGTATCCTGGAGAAGGCAGCACTCGAGCCACCATCTTGGAAGTGAAGAT
CAGGCCCTCAGCAGACACAAAACCTGCCAGTGCCATGATCTTGGATTTTTCAACCTGCAG
GACTGTAAGACATACATTTTTATTATTTATACATTACTCATGCTAAGGTATTCTGTTATAGCTG
CATAAAAAAGAATAAGACAGAATAAGACAGAAGCTGTGTGGGCCTGCATGAGAACTTCAAT
TAACTGTCCATTCTCTAAACAAAACAAAGAAATTGGAGATATGCAGACAGACAGACAGACA
CACACACACACACACACACACACACACACAAAACCCTGGTCATATTTCTAATTCAATTTAA
TACACAATTATTAAA

AGATCACGAAGGTAACAAAGAGTTAGCACTTCACCCCTAGGATAGTGAGAAACCCTTAA
AGGGCTTAAGCAGGGGATTAACCTGAGAAAGATCACTTTTGGGTGCTATGCAGAAAATG
GATTGCAAAGAAACCAGAAGTACAGCGACTAGTTAGGAAGCTGTTGTAATAATTTAAGC
AATGATGATAGTGTCCACAGTAGGAAGAGGCAGCTTATAGACAGGTCTTCTCATCAGAG
AATATGTGTAAGGTCAAGAGGGAATAGACATTGAAATTGTTAGAAAAATAATCTTTAAGAT
GAGTTGAGGAGAAATAACCCACAAAGAATACTAGAAAGGGTGGCCAGAGAAATGGGCG
TAAAACCAGAAGATTGTTTTGGGGGTTCTAAAACAAAATGTCATACTGCATTACTTACATA
CCTGAGAAGAAAAATGCTTTCATATTTGTGTTAGGTGAAAATAAAGCAGTGGCTCACTTT
TAAAAACAAGGCAAAGCATCCAATAAATTATCACTATTATTTGGTTGATTGTCAATAAATTA
ATAGTTTTTTTTGGGGAAAGGAATTGGAGCAAACCTCCTTTATTGGAGGCTCTGTTAGA
GATTTCTTGGGGTTTGGTATCACTTTGCATAGGTAATGAGGCTAATCTATCAGCATCATC
ATGTTATTCCAGTGATGCAGTGATGATAAATGCAGAGTATGCGATAGTGCCAGAGCCTCT
CTATAATTAATTACCATCTTACAGTTACCTTCAGACATTTATTAGAAACATTGAGACTTGGT
TTTAGCAGTCTATCATAGTCGATTTGAGTAAGAAGCTTCTAAATCCCAGCAGTTGTTTTC
ATTAGTTGACAGATTATGAGAAATATGACCATGTGCTCTATGCAAGTCAGAAGAGCCAGA
TACACTTTGTTATCCCCAGGGTTCACAGATCTGCAGGGAAAAGCAGCAGCCACAAAATT
AATAAAGGACTACTTTTCACTCAGACCTATATTTCAAATAACATTTTATGTATTATCTTTGTA
GTTTTTATTAATAGTATCTAATATATATGAACAAAATGTGATTTTTACAGTTTCTGTCTTAGT
TTAGGTTGCTATAACAGGACACCCTTGACTACTTGACTACACAACAATTTATTTATTGAAG
TTCTGGAGGCTGAAAGTCTGTAATCAGGATGTCATCAGGGTTGGGTTCTTGGTGAAGG
CCCTCTTCCTGGTTTGCAGATGACCTCTTCTAGCAGTGTCCTTCACATGTCTTTTCATAG
AGAAAGGCAAGAGCTCTTTCTCTTCTTATAAGGCACTAAGCCCATCATGGGGGCCCCA
ACCTCATAACTTTATCTAAACCTACTGACCTCCCAAAGGACTCACCTCCTAATACCATCC
CATTGGGGGTTAGAATTTCAACGTATCAATTTGGGAAGGATGCAAACATGCAGTCCATA
AGAGTCACTATTCAGGAAATTCTTATAACAGTCCTGTGAAGTCAGTATTTGCAGTGTCTC
CGGTTAACCAGCAAGGGACTAGATGCCTCAAAGTGGTTCTGTTTACCCATGTACAAGTG
TGAGAACTTGACTTTGAATGTAGGCCTTTCAATGCTCTAGTCCTTGGTCATTTGACTTTT
CCAGTGGTTTTAAAAAATGTGTTTGAGCCCAGAATGCTGTCTATTAAAAAACTTAATCTG
GAACAGTTCCTACCCCTGTTTCAAAGGTTCTGATGTACCTTTTCAGAA

Supplemental Figure 5

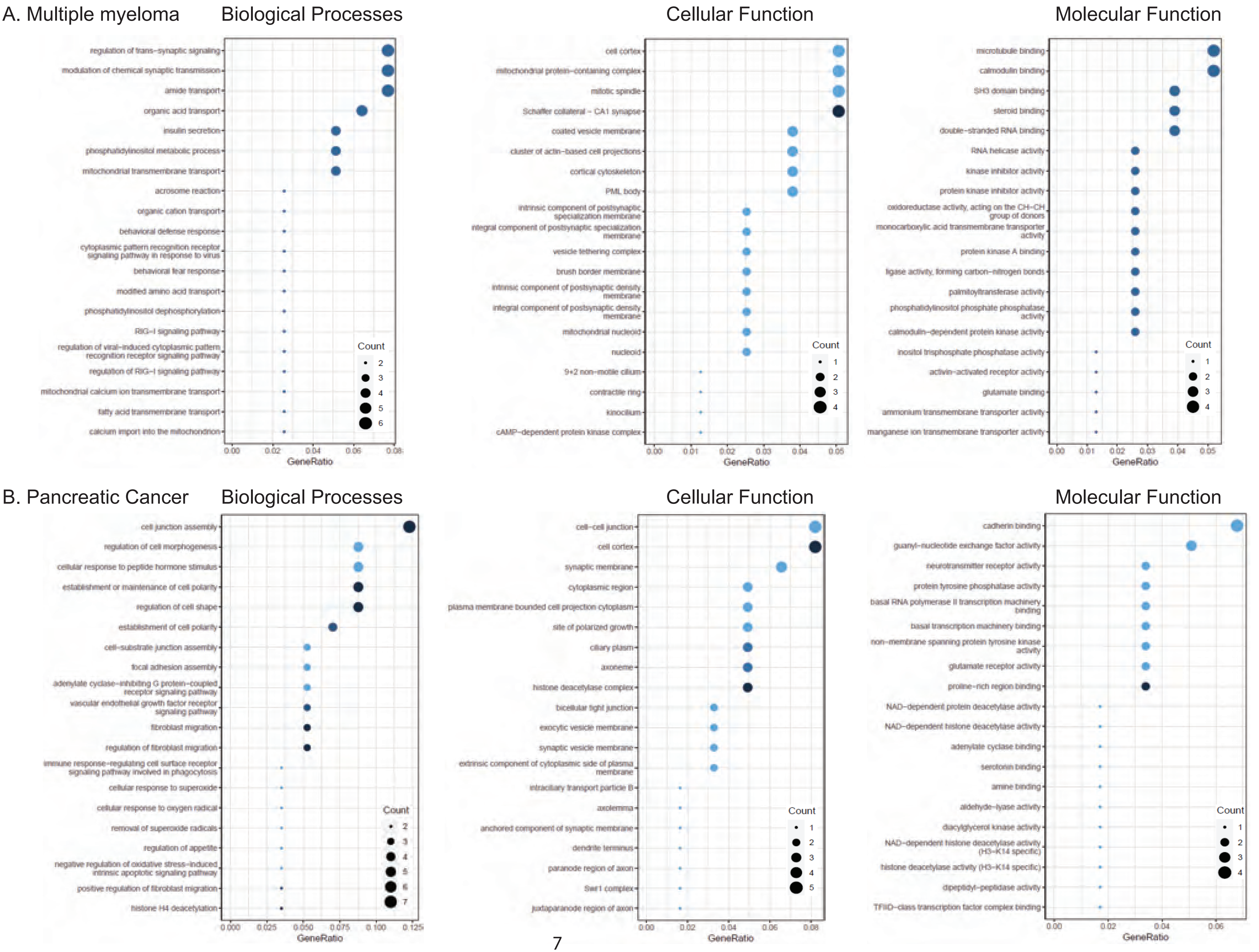

Supplemental Figure 6

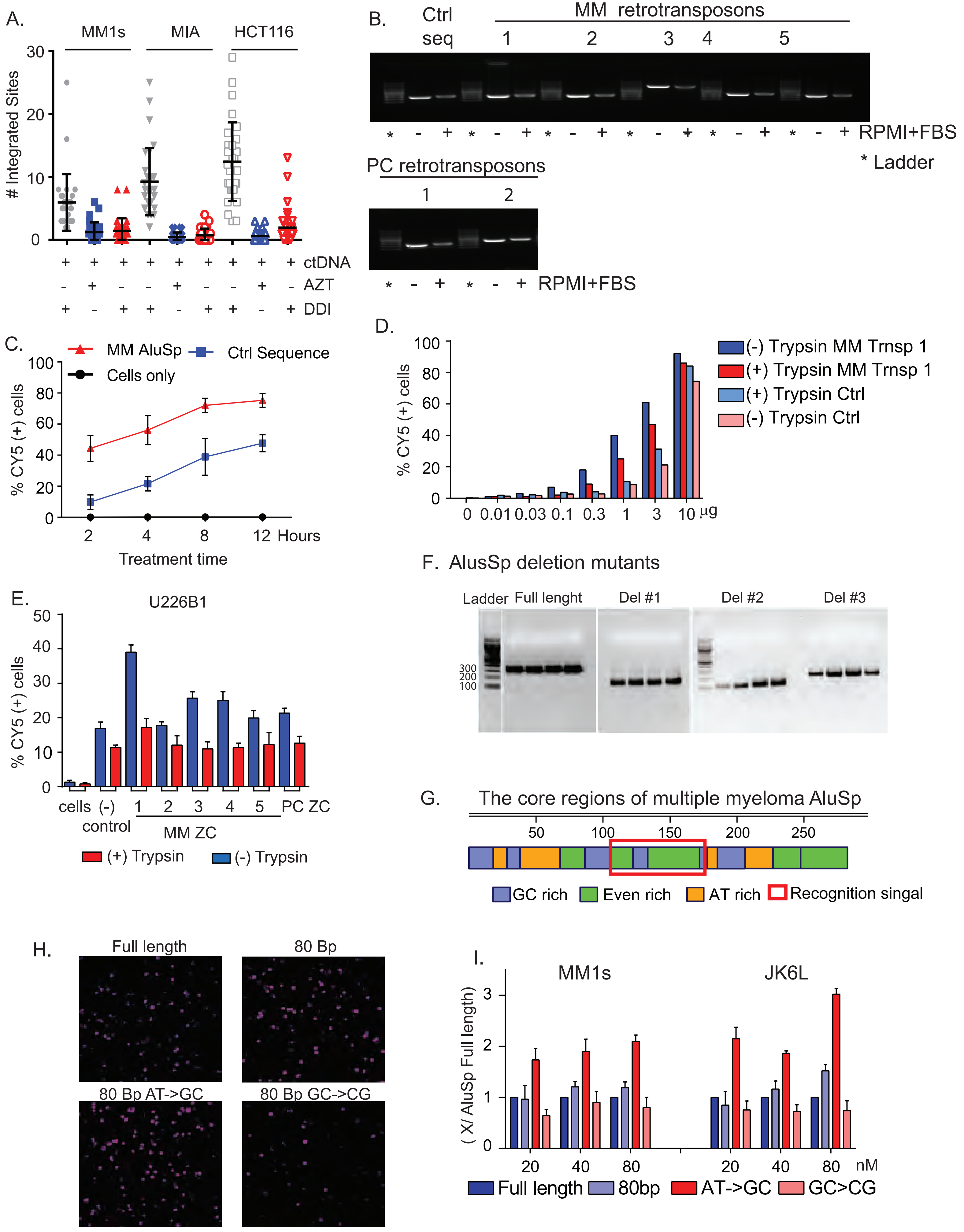

Supplemental Figure 7

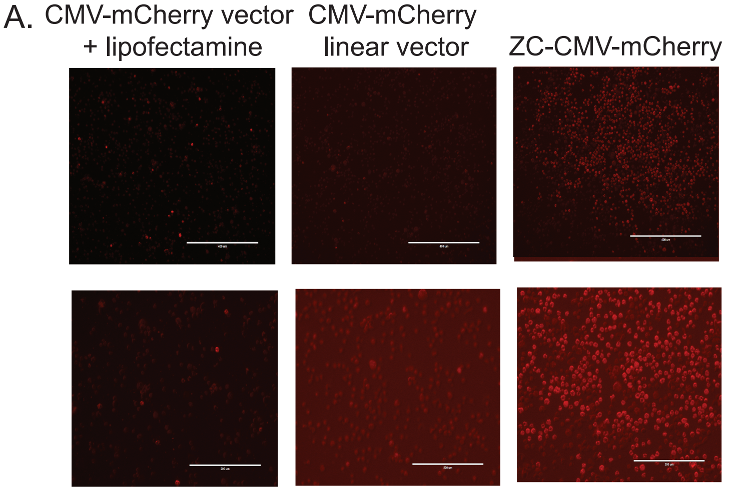

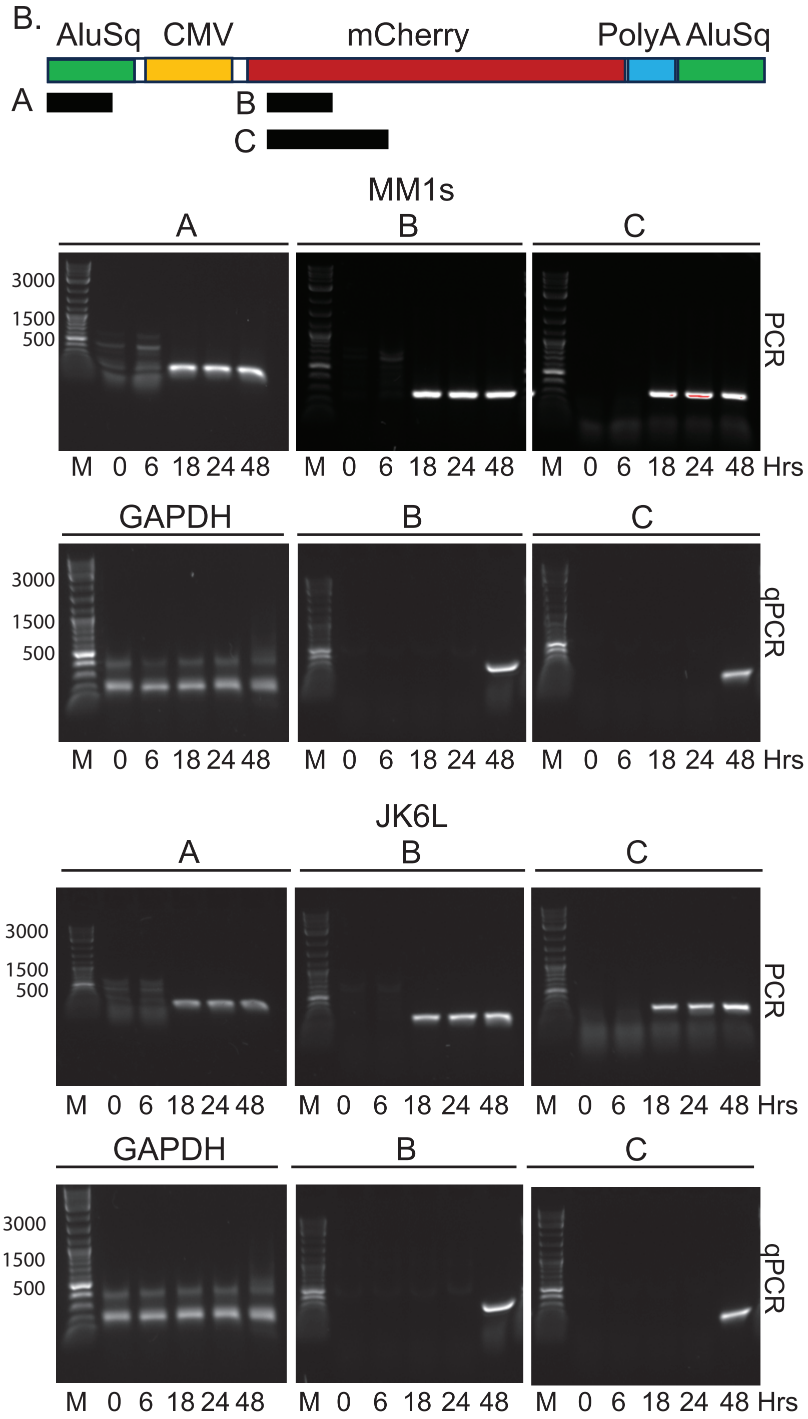

-

Supplemetal Figure 8

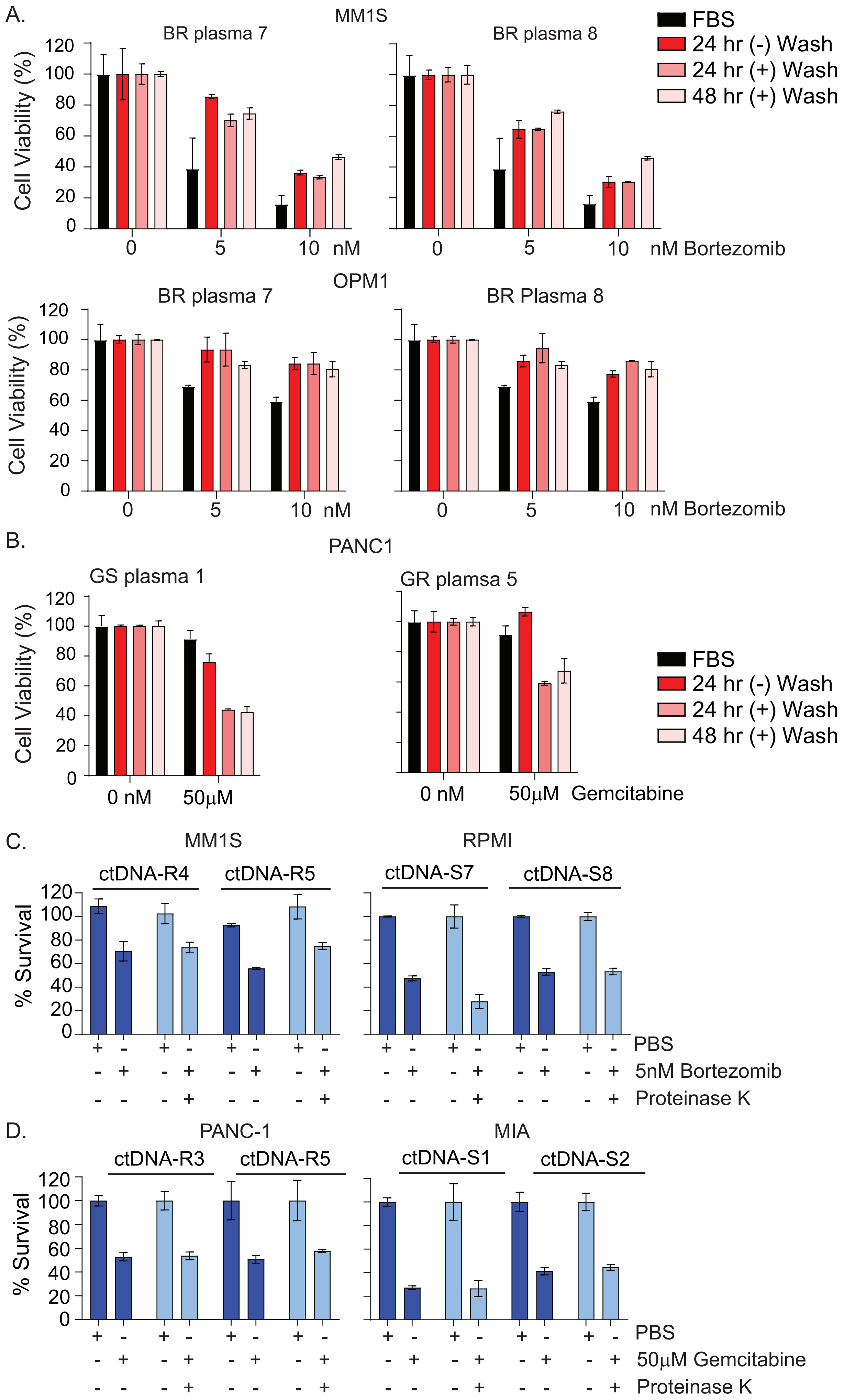

Supplementary Table Legends

Supplemental Table 1A and 1B. Tables summarizing oncogenes enriched by copy number in coculture conditions in MM and PC in comparisons with cell lines alone.

Supplemental Table 2. Table summarizing the number of contigs containing a specific class and type of TE in all inserted and non-inserted contigs in MM and PC coculture experiments. Statistical analysis was performed using the Chi-square test. Significance was determined on the basis of a p-value of <0.05. MM: multiple myeloma, PC: pancreatic cancer.

Supplemental Table 3. Summary table of the TE family members for all inserted contigs from matching multiple myeloma (A) and pancreatic cancer (B) co-culture experiments.

Supplemental Table 4. Supplemental Table 4. Median expression, median absolute deviation (MAD), and expression variation estimation TEs. Ranks are calculated across all genes and transposons.

Supplemental Table 5. Table of reagents and constructs used in the manuscript.

Supplemental Table 1A. Tables summarizing oncogenes enriched by copy number in coculture conditions in MM in comparisons with cell lines alone.

GENE	Insertions	Entrez.Gene.ID	GRCh37.Isoform	GRCh37.RefSeq	GRCh38.Isoform	GRCh38.RefSeq	X..of.occurrence.within.resources..Column.D.J.	OncoKB.Annotated	Is.Oncogene	Is.Tumor.Suppressor.Gene	MSK.IMPACT	MSK.HEME	FOUNDATION.ONE	FOUNDATION.ONE.HEME	Vogelstein	SANGER.CGC.05.30.2017.	Gene.Aliases
No Annotation	105																
MAST2	2																
MCCC1	2																
PLCD4	2																
RP11-308D16.4	2																
TBL1XR1	2	79718	ENST0000043006	NM_024665.4	ENST0000043006	NM_024665.4	4	Yes	No	Yes	No	Yes	No	Yes	No	Yes	C21, DC42, FLJ12894, IRA1, TBLR1
TPTE	2																
ZDHHC1	2																
AADACL4	1																
ABCG2	1																
ABR	1																
AC005154.6	1																
AC006960.7	1																
AC009784.3	1																
AGBL2	1																
AKR1D1	1																
AMHR2	1																
ANKRD29	1																
ANLN	1																
APOL4	1																
ATRX	1	546	ENST0000037334	NM_000489.3	ENST0000037334	NM_000489.3	7	Yes	No	Yes	Yes	Yes	Yes	Yes	Yes	Yes	JMS, MRX52, RAD54, XH2, XNP
C1orf63,RHD	1																
C4orf29	1																
C9orf3	1																
CAMK2G	1																
CLASRP	1																
CLSTN2	1																
COPG1	1																
CPT1A	1																
CTC-459F4.3,LLNLF-65H9.1,CTC-459F4.7	1																
CYP4A22-AS1	1																
DCC	1																
DDX60	1																
DOCK3	1																
DPP6	1																
DUS2	1																
EEF2K	1																
EMCN	1																
ENPP7P8	1																
FAM87B	1																
FBLN2	1																
FBXL20	1																
FCRL5	1																
GAD1	1																
GPR125	1																
GREB1L	1																
HERC2P3	1																
HKR1	1																
HMBOX1	1																
IL27RA	1																
INPP5J	1																
JAKMIP3	1																
KLF13	1																
KLRG1	1																
KRTAP5-5	1																
LINC00669	1																
LIPE-AS1,CEACAM1	1																
LRBA	1																
LRG_510,MPL	1																
LRP2	1																
LUC7L3	1																
MAP4	1																
MCU	1																
MORN1	1																
MUC16	1																
MYRIP	1																
NBAS	1																
NEU3	1																
NFATC2	1	4773					1	No	No	No	No	No	No	No	No	Yes	NF-ATP, NFAT1, NFATp
NHSL1	1																
NPSR1-AS1	1																
NR3C2	1																
NTMT1	1																
PAIP2	1																
PCSK4	1																
PCSK4,CTB-25B13.6	1																
PET112	1																
PIGB	1																
PLEKHB2	1																
PRKAR1B	1																
PRR14L	1																
RIMKLB	1																
RNF4	1																
RP11-107P7.5	1																
RP11-111A22.1,C15orf57	1																
RP11-148L24.1	1																
RP11-161D15.2,RP11-161D15.1	1																
RP11-24I21.1	1																
RP11-24P14.1	1																
RP11-266O8.1	1																
RP11-294O2.2	1																
RP11-2F9.4	1																
RP11-326I11.4	1																
RP11-361M10.3,RP11-1006G14.2	1																
RP11-454C18.2,RP11-64D22.2	1																
RP11-551L14.1	1																
RP11-575L7.4,KIF27	1																
RP11-776A13.3	1																
RP11-81N13.1	1																
RP13-578N3.3	1																
S1PR5	1																
SCAI	1																
SEC14L1	1																
SERPINI1	1																
SLC25A20	1																
SLC39A14	1																
SSR1,RP11-69L16.4	1																
STRC	1																
SUPV3L1	1																
TAF6,RP11-506M12.1	1																
TBCK	1																
TFDP2	1																
TMEM144	1																
TPM4	1	7171					2	No	No	No	No	No	No	Yes	No	Yes	
TRIM16	1																
USP6	1	9098					2	No	No	No	No	No	No	Yes	No	Yes	HRP1, TRE17, TRESMCR, Tre-2, Tre2
VDAC1	1																
ZNF705A	1																

Supplemental Table 1B. Tables summarizing oncogenes enriched by copy number in coculture conditions in PC in comparisons with cell lines alone.

GENE	Insertions	Entrez. Gene.ID	GRCh37. Isoform	GRCh37. RefSeq	GRCh38. Isoform	GRCh38. RefSeq	X..of.occurrence.within.resources..Column.D.J.	OncoKB. Annotated	Is.Oncogene	Is.Tumor.Suppressor. Gene	MSK.IMPACT	MSK.HEME	FOUNDATION .ONE	FOUNDATION .ONE.HEME	Vogelstein	SANGER.CGC.05.30.2017.	Gene.Aliases
No Annota	624																
AIG1	6																
MCF2L2	6																
PRIM2	6																
RABL6	4																
C6orf203	3																
COA1	3																
DAF1	3																
DPP6	3																
EIF2B5,ECE	3																
EYS	3																
GPHN	3	10243					2	No	No	No	No	No	No	Yes	No	Yes	KIAA1385
MACF1	3																
MAD1L1	3																
MIR3654,TU	3																
ALADL2	3																
RP11-296A	3																
RP11-379B	3																
RYR1	3																
SASH1	3																
SNTB2	3																
STAT3,LRG_	3																
STX3	3																
TXNRD1	3																
UBE2D2	3																
AC007970.	2																
ACOT7	2																
ANXA7	2																
APP	2																
ARHGAP19	2																
CCDC77	2																
CDH12	2																
CDH18	2																
CIRH1A,SN	2																
CLEC9A	2																
CLPTM1	2																
COL24A1	2																
COL4A2	2																
COX7A2	2																
CRYBB2P1	2																
CYP4X1	2																
DAPL1	2																
DGKB	2																
EFCAB8	2																
EP400	2	57634	ENST00000389561	NM_015409.3	ENST00000389561	NM_015409.3	2	Yes	No	Yes	No	Yes	No	No	No	No	CAGH32, DKFZP434I225, KIAA1498, KIAA1818, P400, TNRC12
EP400NL	2																
EVA1C	2																
FAM73A	2																
FRMD5	2																
GRB10	2																
GRIK1	2																
GSTM4	2																
GTF2IRD1	2																
GYPC	2																
HIP1	2	3092					2	No	No	No	No	No	No	Yes	No	Yes	
HIPK3	2																
JMY	2																
KCNIP4	2																
KCTD21-AS	2																
LINC00313	2																
MAGI2	2																
MOK	2																
NPSR1-AS1	2																
NRG4	2																
OPA3	2																
OSBPL1A	2																
PDSS2	2																
PGBD2	2																
PHF11	2																
PLD5	2																
POC1B	2																
POU6F2	2																
PTPRN2	2																
RAB6A	2																
RALGPS2	2																
RDX	2																
RELB	2																
RFFL,RAD5	2																
RP11-281O	2																
RP11-538P	2																
RP11-615I2	2																
RP11-849H	2																
RP11-85M1	2																
RP13-20L14	2																
SEC63	2																
SEPT7P2	2																
SLC7A7	2																
SPAG16	2																
SUCLG1	2																
TBC1D32	2																
TNRC18	2																
TTC28	2																
TUBB3	2																
UBTD1	2																
WDR27	2																
ZBTB20	2	26137	ENST00000474710	NM_001164342.2	ENST00000474710	NM_001164342.2	1	Yes	Yes	Yes	No	No	No	No	No	No	DKFZp566F123, DPZF, ODA-8S, ZNF288
ZNF114,CT	2																
ZNF267	2																
ZNF431	2																
ZNF717	2																
ZNF793	2																
ABCC6	1																
AC004538.	1																
AC005537.	1																
AC005592.	1																
AC007879.	1																
AC008060.	1																
AC008984.	1																
AC009410.	1																
AC012593.	1																
AC068196.	1																
AC087499.	1																
AC093415.	1																
AC105393.	1																
ACAD10	1																
ACIN1	1																
ACTB	1	60					1	No	No	No	No	No	No	Yes	No	No	
ADAMTS2	1																
ADAMTSL3	1																
ADCY3	1																
AGPAT3	1																
AHK	1																
AJ006995.3	1																
AKAP12	1																
ALG1L10P	1																
ALS2CR12	1																
AMN1	1																
ANKRD13A	1																
ANKRD18A	1																
ANKRD20A	1																
ANKRD20A	1																
ANKRD30A	1																
ANKRD36C	1																
ANO2	1																
ANO5	1																
AP000230.	1																
AP000320.	1																
AP000344.	1																
AP000936.	1																
AP002856.	1																
AP1G1	1																
AP2A1	1																
AP2A2	1																
AP2B1	1																
APBA3	1																
APPBP2	1																
ARHGAP10	1																
ARHGAP23	1																
ARHGAP39	1																
ARHGAP42	1																
ARL13B,STX	1																
ASB15,RP1	1																
ASCC1	1																
ASPH	1																
ASTN2	1																
ASZ1	1																
ATP5L	1																
ATP6V1C2	1																
AVL9,DPY1	1																
AVL9,NT5C	1																
AVL9,RP9P	1																
B3GNTL1	1																
BACH1,BAC	1																
BAD,GPR13	1																
BAI1	1																
BAIAP2L1	1																
BAZ1B	1																
BBX	1																
BCL7C,MIR	1																
BLM,LRG_2	1																
BZW2	1																
C11orf65	1																
C16orf95	1																
C19orf57	1																
C1orf100,R	1																
C1orf112,S	1																
C2orf42,TIA	1																
C3orf35	1																
C4BPA	1																
C4orf19,RE	1																
C6ORF165,	1																
C6orf183	1																
CAC1C,LRG	1																
CACNG4	1																
CALM2P3	1																
CAMSAP1	1																
CARM1P1	1																
CASC1	1																
CASC4	1																
CATSPER2P	1																
CCBL1	1																
CCDC113	1																
CCDC141	1																
CCDC170	1																
CCDC3	1																
CCNB3	1	85417	00276014	NM_033031.2	00276014	NM_033031.2	1	Yes	Yes	No	No	No	No	No	No	No	
CCNF	1																
CCNY	1																
CCNYL1	1																

CDK14	1																
CDKL3,CTD-	1																
CEACAMP1	1																
CELF6,RP11	1																
CEP68,RAB	1																
CERS6	1																
CHD8	1																
CHKA	1																
CIB1	1																
CKMT2-AS1	1																
CKMT2-AS1	1																
CLDN11	1																
CLMP	1																
CNOT1	1																
CNTN4	1																
CNTN5	1																
CNTP2	1																
COL19A1	1																
CPEB3	1																
CPED1	1																
CPM	1																
CRTC1	1	23373					1	No	No	No	No	No	No	No	No	Yes	FLJ14027,<br>KIAA0616,<br>MECT1,<br>TORC1
CSF3R,LRG_	1																
CSNK1E	1																
CSNK1G1,C	1																
CSPG4P13	1																
CTB-12O2.1	1																
CTB-35F21.	1																
CTC-281M2	1																
CTC-340A1	1																
CTC-490E2	1																
CTC-559E9.	1																
CTD-2034I4	1																
CTD-2303H	1																
CTD-2337A	1																
CTD-2547E	1																
CTD-3074O	1																
CTN3	1																
CYB5B	1																
CYSTM1,PF	1																
DAGLB	1																
DAK,CYB56	1																
DBNDD1	1																
DCLRE1C,LI	1																
DDX46	1																
DEF8	1																
DENND1B	1																
DENND5B	1																
DIABLO,RP1	1																
DIP2C	1																
DH12	1																
DNM1P46	1																
DOPEY2	1																
DPEP1	1																
DPP8	1																
DPY19L2	1																
DSCR4	1																
DT	1																
DTNB	1																
DUS2	1																
DYX1C1-CC	1																
EDAR	1																
EDARADD	1																
EDEM2	1																
EEF2K	1																
EFR3B	1																
EHMT1	1																
EIF2B5	1																
EIF3L	1																
EIF4B,RP11	1																
EIF4G3	1																
ELK2BP	1																
ELMSAN1,R	1																
ELP4	1																
EML5	1																
ENPP7P4,RI	1																
ENPP7P5	1																
EPB41L3	1																
EPS8	1																
EXOC6	1																
EXOSC1	1																
FAHD1,MEI	1																
FAM57A	1																
FAM92A1P	1																
FANCA,LRG	1																
FANCC,LRG	1																
FAT3	1																
FBN3	1																
FCGBP	1																
FGR	1																
FHOD3	1																
FNBP1	1	23048					1	No	No	No	No	No	No	Yes	No	No	FBP17,<br>KIAA0554
FOXN3	1																
FRG1	1																
FRMD4A	1																
FUK	1																
GABRP	1																
GAN,LRG_2	1																
GAS8	1																
GDPD1	1																
GEMIN5	1																
GGT5	1																
GGTA1P	1																
GIGYF2	1																
GLG1	1																
GMDS	1																
GNB1	1	2782	0037860<br>9	282539.<br>1	0037860<br>9	282539.<br>1	3	Yes	Yes	No	Yes	Yes	No	No	No	No	
GNG7	1																
GPATCH1	1																
GPC5	1																
GPR156	1																
GRIK4	1																
GS1-259H1	1																
GTF2IP2	1																
GTF2IRD1P	1																
GXYLT2	1																
GZMAP1	1																
HDAC4	1	9759	ENST000<br>0034561<br>7	NM_006<br>037.3	ENST000<br>0034561<br>7	NM_006<br>037.3	3	Yes	Yes	Yes	No	Yes	No	Yes	No	No	BDMR,<br>HA6116,<br>HD4, HDAC-<br>4, HDAC-A,<br>HDACA,<br>KIAA0288
HDAC9	1																
HEATR2	1																
HELLS	1																
HELZ	1																
HEXDC	1																
HIPK4	1																
HOGA1,PI4	1																
HSP90AA1	1	3320					2	No	No	No	No	No	No	Yes	No	Yes	FLJ31884,<br>HSP90N,<br>HSPC1,<br>HSPCA,<br>Hsp89,<br>Hsp90
HSPE1-MOE	1																
HTR4	1																
HTR7	1																
HUNK	1																
IGF2BP2	1																
IGSF3	1																
IKBKB	1	3551					1	No	No	No	No	No	No	No	No	Yes	IKK-beta, IKK2
IL1RAPL2	1																
IL20RB	1																
IL34	1																
IL9RP2	1																
INPP5A	1																
INPP5D	1	3635					1	No	No	No	No	No	No	Yes	No	No	SHIP, SHIP1,<br>hp51CN
INPPL1	1	3636	0029822<br>9	NM_001<br>567.3	0029822<br>9	NM_001<br>567.3	3	Yes	No	Yes	Yes	Yes	No	No	No	No	SHIP2
INSR	1	3643	0030285<br>0	NM_000<br>208.2	0030285<br>0	NM_000<br>208.2	3	Yes	Yes	No	Yes	Yes	No	No	No	No	CD220
IPP	1																
IQCJ-SCHIP1	1																
IQSEC3	1																
ITGAE	1																
ITIH6	1																
ITSN2	1																
JAZF1	1	221895					1	No	No	No	No	No	No	Yes	No	No	DKFZp761K2<br>222, TIP27,<br>ZNF802
JUP	1																
KCB1	1																
KCNJ6	1																
KCNN1	1																
KCTD20	1																
KDM2A	1																
KDM4A	1																
KIAA0100	1																
KIAA0226,F	1																
KIAA1324	1																
KIAA1324L	1																
KIAA1467	1																
KIAA1522	1																
KIF11	1																
KIF27	1																
KIFC3	1																
KIN	1																
KLC1,XRCC3	1																
KLHL6	1	89857	0034131<br>9	NM_130<br>446	0034131<br>9	NM_130<br>446	3	Yes	Yes	Yes	No	No	Yes	Yes	No	No	FLJ00029
KMT2C	1	58508	ENST000<br>0026218<br>9	NM_170<br>606.2	ENST000<br>0026218<br>9	NM_170<br>606.2	6	Yes	No	Yes	Yes	Yes	No	Yes	Yes	Yes	HALR,<br>KIAA1506,<br>MLL3
KMT2E	1																
LAPTM4A	1																
LARS2	1																
LHX2	1																
LINC00277,	1																
LINC00478	1																
LINC00910	1																
LINC00930	1																
LIPE-AS1	1																
LMF1	1																
LPIN1	1																
LRCH4,FBXO	1																
LRG_212,ZN	1																
LRG_252,KI	1																
LRG_308,PA	1																
LRG_377,AI	1																
LRG_433,LA	1																
LRG_502,FA	1																
LRRC37B	1																
LRRC59	1																
LRRTM4	1																
LSM12	1																
LTBR	1																
MAN2B1	1																
MAP3K14	1	9020	0034468<br>6	NM_003<br>954.3	0034468<br>6	NM_003<br>954.3	4	Yes	Yes	No	Yes	Yes	No	Yes	No	No	FTDCR1B,<br>HS, HSNIK
8-Mar	1																
MARK4,ACC	1																
MARVELD2	1																
MAST2	1																
MATK	1																
MBNL1	1																
MCHR2-AS1	1																

MCM9	1																
MCU	1																
MDN1	1																
MEIOB	1																
MFN1	1																
MFSD1,RP1	1																
MFSD6	1																
MLIP	1																
MLTK	1																
MOGS	1																
MOXD1	1																
MRAP2	1																
MROH2B	1																
MROH7-TT	1																
MRPL33,RB	1																
MRPS17,ZN	1																
MRPS5	1																
MSANTD3,	1																
MTFR2	1																
MTOR	1	2475	ENST00000361445	NM_004958.3	ENST00000361445	NM_004958.3	6	Yes	Yes	No	Yes	Yes	Yes	Yes	No	Yes	FLJ44809, FRAP, FRAP1, FRAP2, RAFT1, RAPT1
MYBBP1A	1																
MYO1C	1																
MYO1D,RP1	1																
MYO1F	1																
PA-AS1	1																
V2	1																
NCOA1	1	8648					1	No	No	No	No	No	No	No	No	Yes	F-SRC-1, KAT13A, NCoA-1, RIP160, bHLHe74
NEURL1B	1																
NFE2L2	1	4780	00397062	NM_006164.4	00397062	NM_006164.4	7	Yes	Yes	No	Yes	Yes	Yes	Yes	Yes	Yes	
NIFK-AS1	1																
NIPA1	1																
NIPA1,RP11	1																
NKAIN1	1																
NLRC5	1																
NLRP12	1																
NLRX1	1																
NMT1	1																
NOD1	1	10392					1	No	No	No	No	No	No	Yes	No	No	CARD4, CLR7.1, NLRC1
NOP58	1																
NPSR1-AS1	1																
NRCAM	1																
NTRK2	1	4915	00277120	NM_006180.3	00277120	NM_006180.3	5	Yes	Yes	No	Yes	Yes	Yes	Yes	No	No	TRKB
NUBPL	1																
NUCB2	1																
NUMA1	1	4926					2	No	No	No	No	No	No	Yes	No	Yes	
NUP160	1																
NUP210L	1																
OBSCN	1																
OFCC1	1																
OPRM1	1																
ORAI1	1																
OSBPL6	1																
OSCP1	1																
OTOGL,RN7	1																
OTUD7A	1																
PACS2	1																
PAK2	1																
PALM	1																
PART1	1																
PCBD2	1																
PCDH15,RP	1																
PCDP1	1																
PCNT	1																
PCNXL2	1																
PCYOX1	1																
PCYT1A,RP1	1																
PDE7B,RP11	1																
PDE9A	1																
PELI3,CTD-3	1																
PFDN2	1																
PGA4,CTD-	1																
PHF13	1																
PI4KA	1																
PIEZO1	1																
PIEZO2	1																
PITPNM1	1																
PLAT	1																
PLCB1	1																
PLCH1	1																
PLEKHA5	1																
PLEKHG1,R	1																
PMS2P3	1																
POFUT2	1																
POLD3	1																
POLE	1	5426	00320574	NM_006231.2	00320574	NM_006231.2	5	Yes	No	Yes	Yes	Yes	Yes	No	No	Yes	POLE1
POLN	1																
POU6F2,PO	1																
PPP1R2P3	1																
PRCP	1																
PRDM10	1																
PRDM16	1	63976					2	No	No	No	No	No	No	Yes	No	Yes	KIAA1675, KMT8F, MEL1, MGC166915, PFM13
PRDX3	1																
PRKACA	1	5566	00308677	NM_002730.3	00308677	NM_002730.3	2	Yes	Yes	No	No	No	No	No	No	Yes	PKACa
PRKDC	1	5591					1	No	No	No	No	No	No	Yes	No	No	D-PKC, D-PKcs, DPK, DPKc, DNPK1, HYRC, HYRC1, XRCC7, p350, p460
PROS1	1																
PRR4,TAS2	1																
PSG1	1																
PSG8	1																
PTK2	1																
PTP4A1	1	7803	00370651	NM_003463.4	00626021	NM_003463.4	3	Yes	No	No	Yes	Yes	No	No	No	No	PRL-1, PTPCAAX1
PTPRT	1	11122	00373198	NM_133170.3	00373198	NM_133170.3	4	Yes	No	Yes	Yes	Yes	No	No	No	Yes	KIAA0283, RPTPrho
QPRT	1																
QRICH1	1																
RAB27B	1																
RABGAP1L	1																
RAD51B	1	5890	ENST00000487270	NM_133509.3	ENST00000487270	NM_133509.3	5	Yes	No	Yes	Yes	Yes	Yes	No	No	Yes	R51H2, RAD51L1, REC2, hREC2
RCBTB2	1																
RCC2,RP1-2	1																
RCOR1	1																
RFC2	1																
RFWD2	1																
RGPD8	1																
RGS7	1																
RHBG	1																
RHOD	1																
RHPN2	1																
RIBC2	1																
RMND1	1																
RNF121	1																
RNF213	1	57674					2	No	No	No	No	No	No	Yes	No	Yes	ALO17, C17orf27, KIAA1554, KIAA1618, MYMY2, NET57
RP1-27O5.3	1																
RP1-283E3.	1																
RP11-101E3	1																
RP11-1082L	1																
RP11-10A14	1																
RP11-1220K	1																
RP11-124N	1																
RP11-1277A	1																
RP11-13N1	1																
RP11-167H	1																
RP11-176N	1																
RP11-179A	1																
RP11-187O	1																
RP11-218I7	1																
RP11-241G	1																
RP11-25K21	1																
RP11-260M	1																
RP11-266L9	1																
RP11-27G24	1																
RP11-32K4.	1																
RP11-340E6	1																
RP11-344E1	1																
RP11-345J4	1																
RP11-347E1	1																
RP11-359J1	1																
RP11-361L1	1																
RP11-379C1	1																
RP11-37B2.	1																
RP11-393J1	1																
RP11-405A1	1																
RP11-415I1	1																
RP11-416O	1																
RP11-426C2	1																
RP11-426L1	1																
RP11-444E1	1																
RP11-467H1	1																
RP11-479J7	1																
RP11-516C1	1																
RP11-525K1	1																
RP11-578F2	1																
RP11-597K2	1																
RP11-598P2	1																
RP11-644C3	1																
RP11-716O	1																
RP11-844P9	1																
RP11-84A19	1																
RP3-331H24	1																
RP3-439F8.	1																
RP4-791C19	1																
RP5-1103G	1																
RP5-896L10	1																
RP5-991G20	1																
RPN1	1	6184					2	No	No	No	No	No	No	Yes	No	Yes	
RUFY1,RP11	1																
RYR2	1																
SARS2,CTC-	1																
SEC11A	1																
SEPP1	1																
9-Sep	1																
SETD1A	1	9739	ENST00000262519	NM_014712.2	ENST00000262519	NM_014712.2	2	Yes	Yes	No	No	Yes	No	No	No	No	KIAA0339, KMT2F, SET1A, Set1
SGK1	1	6446	00237305	NM_005627.3	00237305	NM_005627.3	4	Yes	Yes	No	No	Yes	Yes	Yes	No	No	SGK
SKIL	1																
SLC13A3	1																
SLC16A3,CS	1																
SLC16A8	1																
SLC22A10,S	1																
SLC25A20	1																
SLC25A44	1																
SLC30A9	1																
SLC38A6	1																
SLC8A1-AS1	1																
SMIM11	1																
SOCS2-AS1	1																
SOD2	1																
SORBS1	1																
SPECC1	1																
SPON2	1																
SPTBN2	1																
SPTBN4	1																
ST6GAL1	1																

STAG1	1	10274	00383202	NM_005862.2	00383202	NM_005862.2	2	Yes	No	Yes	No	Yes	No	No	No	No	SA-1, SA1, SCC3A
STEAP1B	1																
STIL	1	6491					1	No	No	No	No	No	No	No	No	Yes	MCPH7, SIL
STIM1,LRG_	1																
STK19	1	8859	00375331	NM_004197.1	00375331	NM_004197.1	3	Yes	Yes	No	Yes	Yes	No	No	No	No	D6S60, G11
STK38L	1																
STON1-GTF	1																
STX18-AS1	1																
SYNPO2L,R	1																
SYT14	1																
TACR2	1																
TANGO6	1																
TBC1D19	1																
TCIRG1,LRG	1																
TEAD4	1																
TIAM2	1																
TJP1	1																
TLL2	1	7093					1	No	No	No	No	No	No	Yes	No	No	
TMCC1	1																
TMEM120B	1																
TMEM181	1																
TMEM183A	1																
TMEM184C	1																
TMEM199,(	1																
TMTC2	1																
TNFRSF13B	1																
TNS3	1																
TOMM20L,	1																
TPD52L2	1																
TPM3	1	7170					2	No	No	No	No	No	No	Yes	No	Yes	NEM1, TRK
TPRA1	1																
TPT1-AS1	1																
TPTE	1																
TPTE2	1																
TRAF3IP1	1																
TRIM54	1																
TRRAP	1	8295					1	No	No	No	No	No	No	No	No	Yes	PAF400, TR-AP, Tra1
TSC22D2	1																
TSPEAR	1																
TTC27	1																
TTC40	1																
TXLNG	1																
UBE4B	1																
UGT1A8,UG	1																
UIMC1	1																
UNC93A	1																
USP10	1																
USP20	1																
VAC14	1																
VKORC1L1	1																
VPS13A	1																
VPS13B	1																
VPS18	1																
WDPCP	1																
WDR47	1																
WWOX	1																
WWTR1	1	25937	00360632	168280.1	00360632	168280.1	4	Yes	Yes	No	Yes	Yes	No	No	No	Yes	DKFZp586I1419
XIRP2	1																
XKR4	1																
XRCC2	1	7516	00359321	NM_005431.1	00359321	NM_005431.1	4	Yes	No	Yes	Yes	Yes	Yes	No	No	No	FANCU
XYLB	1																
YWHAEP7	1																
ZC3H14	1																
ZCCHC4	1																
ZEB2	1																
ZNF223,ZNF	1																
ZNF28	1																
ZNF287,RP1	1																
ZNF341	1																
ZNF41	1																
ZNF415	1																
ZNF418	1																
ZNF442	1																
ZNF512B,PR	1																
ZNF630	1																
ZNF669	1																
ZNF670	1																
ZNF675	1																
ZNF689	1																
ZNF700,ZNF	1																
ZNF709,ZNF	1																
ZNF725P	1																
ZNF726,CTE	1																
ZNF747,ZNF	1																
ZNF808,ZNF	1																
ZNF90	1																

Supplemental Table 2. Table summarizing the distribution of the contigs containing transposons and the fraction of transposons observed at 5’ or 3’ end in inserted vs. non-inserted ctDNA fragments.

	Multiple Myeloma		Pancreatic Cancer		P value
	Inserted	Non-inserted	Inserted	Non-inserted	
Trasnposon/Contig	1.5	1.08	1.8	1.3	<0.001
% Contigs with Transposons	77	68.5	81.5	74	<0.001

Supplemental Table 3A. Summary table of the TE family members for all inserted contigs from matchingmultiple myeloma co-culture experiments.

Transposon	Location relative to insertion	Contig position	Insertion position	Transposon position	Transposon orientation	Distance from insertion	blast chr	blast position	blast orientation
AluSx	intersect	1-2229	1616-1669	1570-1871	reverse complement	0	chr2	86528424 - 86530643	Plus/Plus
L1MB3	right	1-2677	1616-1669	1737-2677	reverse complement	-68	chr2	86528424 - 86530643	Plus/Plus
L2a	intersect	1-2331	1372-2331	941-1454	direct	0	chr14	32672352-32670273	Plus/Minus
THE1C	intersect	1-2519	1344-2990	2050-2519	direct	0	chr14	32672352-32670273	Plus/Minus
AluY	intersect	1-2656	2355-2461	2346-2656	reverse complement	0	chr13	22562455-22559956	Plus/Minus
]L1ME4b	intersect	1-2686	2390-2477	1767-2686	reverse complement	0	chr13	22562455-22559956	Plus/Minus
AluSx	intersect	1-3772	2547-2494	1573-1880	direct	0	chr17		
AluY	intersect	1-3123	419-446	153-469	reverse complement	0	chr12 LIM domain only 3	58715-55601	Plus/Minus
]AluY	intersect	1-1628	1327-1433	1327-1628	reverse complement	0	chr8	15650931-15649499	Plus/Minus
AluY	intersect	1-2816	2445-2709	2505-2816	direct	0	chr3	156308018-156310801	Plus/Plus
AluJb	intersect	1-1096	277-318	1-317	direct	0	chr10	103114349-103115218	Plus/Plus
AluSx	intersect	1-1096	227-318	1-312	direct	0	chr10	103114349-103115218	Plus/Plus
MER11C	intersect	1-3272	1584-3024	2188-3272	reverse complement	0	chr14	46404852-46401883	Plus/Plus
AluY	intersect	1-1632	213-261	1-311	reverse complement	0	chr2	178310609-178312037	Plus/Plus
MER11B	left	1-2915	2789-2911	1-1235	reverse complement	1554	chr1	145617544-145619382	Plus/Plus
AluSx	intersect	1-1235	1031-1111	883-1190	reverse complement	0	chr3	180870641-180871874	Plus/Plus

AluY	intersect	1-1786	133-284	1-312	direct	0	chr6	143000057-143001731	Plus/Plus
AluSx	intersect	1-4743	381-552	261-574	direct	0	chr5	71047678-71042940	Plus/Minus
AluY	intersect	1-4743	381-552	261-574	direct	0	chr5	71047678-71042940	Plus/Minus
Tigger3a	right	1-4764	40-210	2599-2941	direct	2389	chr5	71047678-71042940	Plus/Minus

Supplemental Table 3B. Summary table of the TE family members for all inserted contigs from matching pancreatic cancer co-culture experiments.

Transposon	Location relative to insertion	Contig position	Insertion position	Transposon position	Transposon orientation	Distance from insertion	blast chr	blast position	blast orientation
AluJb	right	1-3705	51-220	3394-3705	direct	+3174	chr18	23191079-23187483	Plus/Minus
AluSx	right	1-3705	145-220	3394-3705	direct	+3174	chr18	23191079-23187483	Plus/Minus
L1PA15	right	1-7716	84-279	6793-7704	reverse complement	-6514	chr4	74809282-74804853	Plus/Minus
L1PB4	right	1-7716	84-279	6801-7704	reverse complement	-6522	chr4	74809282-74804853	Plus/Minus
AluSx	intersect	1-3368	198-336	169-483	direct	0	chr20	60039432-60036075	Plus/Minus
AluSx	intersect	1-4730	4456-4591	4409-4720	direct	0	chr16	30075661-30080375	Plus/Plus
MIRc	left	1-4739	4465-4600	2056-2259	reverse complement	+2206	chr16	30075661-30080375	Plus/Plus
AluJb	intersect	1-3676	2619-2651	2450-2747	reverse complement	0	chr7	78202685-78206359	Plus/Plus
AluSx	intersect	1-3676	2619-2651	2450-2747	reverse complement	0	chr7	78202685-78206359	Plus/Plus
AluSx	intersect	1-7197	1963-1980	1677-1989	reverse complement	0	chr8	17791254-17789008	Plus/Plus
AluY	intersect	1-7197	6052-6112	5828-6138	reverse complement	0	chr8	17791254-17789008	Plus/Plus

MIRb	left	1-7240	5476-5518	4109-4377	direct	-1099	chr8	17791254-17789008	Plus/Plus
MIRc	left	1-7205	5441-5483	4106-4324	direct	-1117	chr8	17791254-17789008	Plus/Plus
AluSx	intersect	1-3669	3257-3375	3270-3567	direct	0	chr3	128761943-128759611	Plus/Plus
MLT2B1	intersect	1-3763	3352-3470	2852-3356	direct	0	chr3	128761943-128759611	Plus/Plus
AluSx	intersect	1-7277	1421-3369	2841-3166	reverse complement	0	chr21	39057278-39054368	Plus/Plus
L2a	intersect	1-7340	1421-3654	2474-2881	direct	0	chr21	39057278-39054368	Plus/Plus
MLT1J2	intersect	1-7311	1473-3625	1167-1529	direct	0	chr21	39057278-39054368	Plus/Plus
AluSx	intersect	1-4351	989-1127	960-1264	direct	0	chr7	22688622-22692178	Plus/Plus
AluY	intersect	1-4251	989-1127	960-1264	direct	0	chr7	22688622-22692178	Plus/Plus
L1ME4b	right	1-4422	989-1127	3057-3720	direct	+1930	chr7	22688622-22692178	Plus/Plus
MIRb	intersect	1-9468	3869-3929	3790-3904	direct	0	chr14	101625831-101623094	Plus/Minus
MER41E	right	1-5966	2493-2559	5071-5660	direct	+2512	chr2	240694078-240699987	Plus/Plus
AluSx	intersect	1-7125	2220-2359	2191-2502	direct	0	chr9	77999115-78001422	Plus/Plus
Mam_R4	intersect	1-7466	2269-2410	1015-4872	direct	0	chr9	77999115-78001422	Plus/Plus
MIRb	left	1-7141	2236-2375	281-550	reverse complement	+1686	chr9	77999115-78001422	Plus/Plus
MIRb	right	1-10415	2751-2783	3949-4170	reverse complement	-1166	chr4	155243887-155240822	Plus/Minus

Supplemental Table 4A. Multiple Myeloma. Median expression, median absolute deviation (MAD), and expression variation estimation TEs. Ranks are calculated across all genes and transposons.

Transposable element	Median Expression	Obs.MAD	Exp.MAD	EV	EV.pvalue
MIRb:MIR:SINE	16.42452619	0.293837	0.764082	-0.47024	0.001174
UCON4:UCON4:Unknown	5.28630054	0.225595	0.684546	-0.45895	0.002207
MER70C:ERVL:LTR	5.437504319	0.223497	0.682194	-0.4587	0.002254
MER65D:ERV1:LTR	6.325347484	0.237214	0.673479	-0.43626	0.005728
Charlie11:hAT-Charlie:DNA	3.948138858	0.25965	0.693243	-0.43359	0.006385
MamRep1151:LTR:LTR?	6.806633164	0.22651	0.65915	-0.43264	0.00662
MamGypLTR2b:Gypsy:LTR	6.537076009	0.239388	0.668908	-0.42952	0.007371
MER89-int:ERV1:LTR	6.415874639	0.243337	0.671804	-0.42847	0.007559
MER6C:TcMar-Tigger:DNA	4.334143943	0.267216	0.694173	-0.42696	0.007887
LTR10F:ERV1:LTR	6.112025228	0.249348	0.676187	-0.42684	0.007981
MER95:ERV1:LTR	4.939624647	0.263106	0.689387	-0.42628	0.008122
LTR81B:Gypsy:LTR	6.683757999	0.24635	0.664133	-0.41778	0.010516
MamGypLTR1d:Gypsy:LTR	6.02614937	0.259781	0.676939	-0.41716	0.010657
MER34C:ERV1:LTR	3.825579451	0.275632	0.692148	-0.41652	0.010939
L2b:L2:LINE	15.65831427	0.293151	0.709587	-0.41644	0.010986
MER65B:ERV1:LTR	5.049375037	0.272309	0.687968	-0.41566	0.011221
PABL_B-int:ERV1:LTR	5.988747832	0.263779	0.677229	-0.41345	0.011878
UCON78:DNA:DNA	4.524625074	0.282155	0.693338	-0.41118	0.012629
LTR102_Mam:ERVL:LTR	6.913024099	0.243735	0.654297	-0.41056	0.012817
ERVL47-int:ERVL:LTR	4.74519846	0.283089	0.691553	-0.40846	0.013991
MIRc:MIR:SINE	15.24706283	0.275302	0.683523	-0.40822	0.014131
MIR3:MIR:SINE	15.23721078	0.276343	0.682925	-0.40658	0.014507
MER75:PiggyBac:DNA	6.801704976	0.253935	0.659364	-0.40543	0.014977
MER99:hAT:DNA	5.255327422	0.280285	0.685015	-0.40473	0.01554
MER92C:ERV1:LTR	3.786286105	0.289002	0.691716	-0.40271	0.016479
LTR9C:ERV1:LTR	4.549513064	0.293674	0.693178	-0.3995	0.017746
Ricksha_0:MULE-MuDR:DNA	6.794519899	0.261631	0.659674	-0.39804	0.018451
Mam_R4:Dong-R4:LINE	6.836820573	0.260869	0.657817	-0.39695	0.018779
Tigger17:TcMar-Tigger:DNA	3.91254862	0.297508	0.692965	-0.39546	0.019296
LTR26E:ERV1:LTR	5.886175621	0.284021	0.677957	-0.39394	0.020094
UCON6:UCON6:Unknown	4.772162765	0.298181	0.691282	-0.3931	0.020376
UCON80_AMi:UCON80_AMi:Unknown	4.303043253	0.301752	0.694239	-0.39249	0.020704
LTR27:ERV1:LTR	5.495895464	0.289672	0.681376	-0.3917	0.020939
UCON34:UCON34:Unknown	4.445280039	0.303368	0.693773	-0.39041	0.021502
LTR53-int:ERVL:LTR	5.674360896	0.289856	0.679506	-0.38965	0.02169
LTR3:ERVK:LTR	4.682893405	0.3032	0.692136	-0.38894	0.021972
MER72B:ERV1:LTR	4.574431825	0.3074	0.693006	-0.38561	0.023568
Charlie4:hAT-Charlie:DNA	5.921039118	0.292842	0.677717	-0.38488	0.023944
MER57B1:ERV1:LTR	7.395666408	0.245236	0.630093	-0.38486	0.023991
MER92A:ERV1:LTR	4.020428707	0.308997	0.69371	-0.38471	0.024085
LTR68:ERV1:LTR	5.183149584	0.302137	0.686087	-0.38395	0.024601
LTR47B2:ERVL:LTR	4.78213191	0.30779	0.69118	-0.38339	0.024977
MER61D:ERV1:LTR	5.309075443	0.302385	0.684197	-0.38181	0.025915
LTR1F:ERV1:LTR	4.435017512	0.312771	0.69382	-0.38105	0.026338

LTR38A1:ERV1:LTR	4.725345436	0.311221	0.691745	-0.38052	0.02662
LTR88c:Gypsy:LTR	6.220039894	0.29613	0.674999	-0.37887	0.0277
MIR:MIR:SINE	15.92123698	0.349826	0.727413	-0.37759	0.028263
MER34C_:ERV1:LTR	6.680439894	0.287195	0.664256	-0.37706	0.028498
MamGypLTR3:Gypsy:LTR	6.657156279	0.288202	0.665103	-0.3769	0.028545
MamGypLTR2c:Gypsy:LTR	6.6831388	0.287413	0.664156	-0.37674	0.028592
MER61A:ERV1:LTR	7.093796234	0.269063	0.645335	-0.37627	0.028873
LTR13_:ERVK:LTR	6.837569808	0.282367	0.657783	-0.37542	0.029812
AluY:Alu:SINE	16.6761329	0.408352	0.783676	-0.37532	0.029953
LTR2C:ERV1:LTR	6.612666588	0.293186	0.666628	-0.37344	0.031127
FordPrefect_a:hAT-Tip100:DNA	4.765954882	0.318652	0.691345	-0.37269	0.031643
MER94B:hAT-Blackjack:DNA	6.834548331	0.286011	0.657918	-0.37191	0.032207
MLT2C2:ERVL:LTR	7.766272531	0.243972	0.613632	-0.36966	0.03338
MER57E2:ERV1:LTR	3.714939823	0.32165	0.690832	-0.36918	0.034131
LTR16D:ERVL:LTR	7.154866571	0.273473	0.642208	-0.36874	0.034507
LTR1C:ERV1:LTR	4.582260784	0.324379	0.69295	-0.36857	0.034648
LTR47B4:ERVL:LTR	5.162270915	0.318173	0.68639	-0.36822	0.03507
LTR18B:ERVL:LTR	6.627670117	0.299069	0.666128	-0.36706	0.035587
MER44D:TcMar-Tigger:DNA	7.196533195	0.275434	0.64007	-0.36464	0.036667
MER66C:ERV1:LTR	6.332931447	0.308951	0.673352	-0.3644	0.036808
MER41D:ERV1:LTR	6.767949388	0.296411	0.6608	-0.36439	0.036854
MamGypLTR1a:Gypsy:LTR	7.373800964	0.267367	0.631152	-0.36378	0.037277
LTR65:ERV1:LTR	7.164452921	0.279148	0.641716	-0.36257	0.038169
MER47C:TcMar-Tigger:DNA	6.606046743	0.304766	0.666844	-0.36208	0.038451
UCON74:DNA:DNA?	4.713102124	0.329872	0.691861	-0.36199	0.038545
MLT-int:ERVL-MaLR:LTR	5.933928406	0.316443	0.677627	-0.36118	0.039484
UCON105:UCON105:Unknown	6.649179482	0.305224	0.665386	-0.36016	0.040657
LTR3A:ERVK:LTR	7.759341857	0.254132	0.613964	-0.35983	0.04108
MADE2:TcMar-Mariner:DNA	8.740808054	0.210774	0.569018	-0.35824	0.042723
LTR72B:ERV1:LTR	3.882150892	0.334816	0.692701	-0.35789	0.043052
MER57C1:ERV1:LTR	5.341391303	0.32694	0.683698	-0.35676	0.044038
MER127:TcMar-Tigger:DNA	3.971448446	0.337042	0.693409	-0.35637	0.04446
MLT1J2-int:ERVL-MaLR:LTR	6.175876758	0.319585	0.675524	-0.35594	0.044648
LTR9A1:ERV1:LTR	7.790465601	0.257793	0.612462	-0.35467	0.045869
MER92-int:ERV1:LTR	6.699732519	0.3091	0.66353	-0.35443	0.046291
L5:RTE-X:LINE	7.036679997	0.295537	0.648232	-0.35269	0.047324
MER121:hAT:DNA	6.264799688	0.322417	0.674403	-0.35199	0.047934
LTR26:ERV1:LTR	6.715995745	0.311324	0.662902	-0.35158	0.048263
MER66-int:ERV1:LTR	7.08148398	0.295455	0.645963	-0.35051	0.049108
MER92D:ERV1:LTR	4.515379924	0.343358	0.693395	-0.35004	0.049484
L2c:L2:LINE	15.81179312	0.37009	0.719882	-0.34979	0.049718
MLT1F1-int:ERVL-MaLR:LTR	5.288731614	0.335314	0.684509	-0.34919	0.050235
LOR1a:ERV1:LTR	7.428489514	0.279545	0.628529	-0.34898	0.050516
LTR53B:ERVL:LTR	4.492565459	0.345659	0.693528	-0.34787	0.051408
MER45A:hAT-Tip100:DNA	9.82419265	0.19429	0.541807	-0.34752	0.051784
Arthur1C:hAT-Tip100:DNA	7.21777262	0.291569	0.638982	-0.34741	0.051878
Kanga1c:TcMar-Tc2:DNA	7.826900749	0.263252	0.610664	-0.34741	0.051925

LTR38C:ERV1:LTR	5.288025963	0.337347	0.684519	-0.34717	0.052113
Tigger6b:TcMar-Tigger:DNA	5.901345343	0.33069	0.677853	-0.34716	0.05216
MER135:DNA:DNA	6.080467665	0.329408	0.676479	-0.34707	0.052254
MER91C:hAT-Tip100:DNA	8.119538718	0.24943	0.595421	-0.34599	0.053286
LTR3B:ERVK:LTR	3.815077541	0.346273	0.692036	-0.34576	0.053568
LTR52-int:ERVL:LTR	4.237144524	0.349481	0.694301	-0.34482	0.054601
LTR106_Mam:LTR:LTR	6.352564472	0.32835	0.673014	-0.34466	0.054883
LTR2B:ERV1:LTR	7.044222353	0.30383	0.647851	-0.34402	0.055352
HERV30-int:ERV1:LTR	3.838406729	0.349387	0.69228	-0.34289	0.056526
L2a:L2:LINE	16.25791924	0.408882	0.751571	-0.34269	0.056854
L1MC3:L1:LINE	12.20759562	0.215515	0.558038	-0.34252	0.05723
MER110:ERV1:LTR	5.750887711	0.336603	0.678902	-0.3423	0.057465
UCON37:UCON37:Unknown	3.863554608	0.350261	0.692528	-0.34227	0.057512
LTR81A:Gypsy:LTR	5.928837969	0.336711	0.677662	-0.34095	0.058685
Charlie9:hAT-Charlie:DNA	8.375293862	0.243848	0.58271	-0.33886	0.060845
LTR59:ERV1:LTR	4.336282712	0.355309	0.694168	-0.33886	0.060892
UCON49:L2:LINE	4.418587411	0.35583	0.693892	-0.33806	0.061784
ffrom PC co-culture experiments. .	3.80965209	0.355289	0.691977	-0.33669	0.063192
Charlie17:hAT-Charlie:DNA	7.961828595	0.267359	0.603734	-0.33638	0.063568
AluYh9:Alu:SINE	4.249752048	0.358215	0.694298	-0.33608	0.064038
MamGypsy2-I:Gypsy:LTR	7.763195211	0.278351	0.61378	-0.33543	0.064883
LTR42:ERVL:LTR	7.531078167	0.288473	0.623881	-0.33541	0.06493
UCON39:TcMar-Tigger:DNA	4.114025344	0.359193	0.694115	-0.33492	0.065634
LTR39-int:ERV1:LTR	4.347015349	0.359535	0.69414	-0.3346	0.066056
LTR90A:LTR:LTR	6.235435873	0.340889	0.674801	-0.33391	0.067042
Ricksha_a:MULE-MuDR:DNA	4.0101113	0.361307	0.693652	-0.33234	0.069061
MER57A1:ERV1:LTR	7.61567489	0.28828	0.620375	-0.3321	0.06939
Tigger5b:TcMar-Tigger:DNA	8.871784304	0.233043	0.564568	-0.33153	0.069906
LTR86A2:ERVL:LTR	6.005851738	0.346645	0.677098	-0.33045	0.07108
MLT1J1-int:ERVL-MaLR:LTR	5.284526113	0.354685	0.684573	-0.32989	0.071502
Charlie17b:hAT-Charlie:DNA	5.570138458	0.350654	0.680498	-0.32984	0.071643
LTR105_Mam:ERVL:LTR	6.156459437	0.346207	0.675736	-0.32953	0.07216
LTR47A:ERVL:LTR	7.386536088	0.301931	0.630533	-0.3286	0.073005
MER68-int:ERVL:LTR	6.570528081	0.339869	0.66795	-0.32808	0.073756
Tigger11a:TcMar-Tigger:DNA	7.040247577	0.31999	0.648052	-0.32806	0.073897
Penelope1_Vert:Penelope:LINE	7.095248281	0.317798	0.645261	-0.32746	0.074554
X2_LINE:CR1:LINE	4.718820763	0.364511	0.691807	-0.3273	0.07493
LTR7C:ERV1:LTR	7.936802571	0.278991	0.605043	-0.32605	0.076479
LTR66:ERVL:LTR	5.023811061	0.363063	0.688309	-0.32525	0.077559
MERX:TcMar-Tigger:DNA	6.049765969	0.351868	0.676745	-0.32488	0.077981
LTR10B:ERV1:LTR	3.670990856	0.365367	0.690224	-0.32486	0.078028
LTR108e_Mam:ERVL:LTR	3.986179845	0.368936	0.693506	-0.32457	0.078357
LTR10E:ERV1:LTR	7.014758287	0.326202	0.649331	-0.32313	0.080469
7SK:RNA:RNA	6.596594011	0.344306	0.667147	-0.32284	0.080892
MamGypsy2-LTR:Gypsy:LTR	6.188707703	0.352695	0.675377	-0.32268	0.081033
LTR56:ERV1:LTR	7.975945734	0.280742	0.602993	-0.32225	0.081408
LTR29:ERV1:LTR	6.972712496	0.329277	0.651414	-0.32214	0.081784

LTR57:ERVL:LTR	5.618866668	0.358417	0.680004	-0.32159	0.082488
AluYk11:Alu:SINE	8.26569212	0.266559	0.587935	-0.32138	0.082723
LTR88b:Gypsy:LTR	7.043051676	0.326831	0.647911	-0.32108	0.083239
HERV4_I-int:ERV1:LTR	7.195150227	0.319401	0.640141	-0.32074	0.083568
(CATTC)n:Satellite:Satellite	3.22174249	0.36055	0.681264	-0.32071	0.083615
MER70A:ERVL:LTR	6.380644563	0.352722	0.672501	-0.31978	0.084695
LTR1:ERV1:LTR	5.96176728	0.358184	0.677428	-0.31924	0.085399
MER41C:ERV1:LTR	7.366031082	0.314242	0.631531	-0.31729	0.087887
LTR84a:ERVL:LTR	6.689091316	0.346679	0.663933	-0.31725	0.087981
LTR16D2:ERVL:LTR	4.956384816	0.37196	0.689178	-0.31722	0.088075
PRIMA41-int:ERV1:LTR	6.760998485	0.344848	0.661089	-0.31624	0.089061
ERV24B_Prim-int:ERV1:LTR	6.52524745	0.353354	0.669228	-0.31587	0.089531
LTR33B:ERVL:LTR	7.838603468	0.294494	0.610078	-0.31558	0.090094
LTR22B1:ERVK:LTR	4.461473953	0.378758	0.693694	-0.31494	0.090986
MLT1-int:ERVL-MaLR:LTR	6.100428904	0.361813	0.676297	-0.31448	0.091596
LTR103_Mam:ERV1:LTR	6.154966275	0.361913	0.675752	-0.31384	0.0923
MER81:hAT-Blackjack:DNA	9.677319673	0.229912	0.543718	-0.31381	0.092394
L1M5:L1:LINE	13.84690685	0.297511	0.611192	-0.31368	0.092676
LTR26D:ERV1:LTR	6.171199011	0.362121	0.675576	-0.31345	0.092958
MER72:ERV1:LTR	7.553360939	0.309732	0.622929	-0.3132	0.093146
Kanga1a:TcMar-Tc2:DNA	7.097477095	0.33196	0.645147	-0.31319	0.093192
X8_LINE:CR1:LINE	5.899897762	0.365024	0.677863	-0.31284	0.093756
LTR75:ERVL:LTR	6.74682048	0.349075	0.661671	-0.3126	0.094131
Kanga1:TcMar-Tc2:DNA	6.89816003	0.342769	0.654999	-0.31223	0.094648
BSR/Beta:Satellite:Satellite	8.018079077	0.288592	0.600772	-0.31218	0.094742
LTR16D1:ERVL:LTR	5.67707565	0.369118	0.679483	-0.31037	0.097606
MADE1:TcMar-Mariner:DNA	9.264590995	0.242431	0.552347	-0.30992	0.098122
LTR47A2:ERVL:LTR	5.658018678	0.370188	0.679646	-0.30946	0.098638
LTR24B:ERV1:LTR	8.192539331	0.283588	0.591629	-0.30804	0.100376
UCON71_Crp:UCON71_Crp:Unknown	5.153633342	0.37978	0.686514	-0.30673	0.101972
MER47B:TcMar-Tigger:DNA	7.351532114	0.325558	0.632242	-0.30668	0.102066
LTR45B:ERV1:LTR	6.915478119	0.347724	0.654181	-0.30646	0.1023
LTR14:ERVK:LTR	5.058047566	0.381509	0.687851	-0.30634	0.102441
L1ME1:L1:LINE	13.4995345	0.290958	0.597121	-0.30616	0.102535
UCON132b:hAT-Tip100:DNA	6.805065448	0.353464	0.659218	-0.30575	0.102817
MER88:ERVL:LTR	5.034930916	0.382409	0.688162	-0.30575	0.102864
Tigger5:TcMar-Tigger:DNA	6.9217429	0.348502	0.653882	-0.30538	0.103662
MER50-int:ERV1:LTR	7.274172725	0.330787	0.636109	-0.30532	0.103709
LTR10D:ERV1:LTR	5.844473908	0.373596	0.678242	-0.30465	0.10493
LTR53:ERVL:LTR	7.68404371	0.313747	0.617443	-0.3037	0.105869
LTR21A:ERV1:LTR	5.477643435	0.37814	0.681618	-0.30348	0.106197
Zaphod2:hAT-Tip100:DNA	7.264632384	0.33384	0.636593	-0.30275	0.107559
LTR9D:ERV1:LTR	7.723229896	0.313301	0.615661	-0.30236	0.108122
MER52-int:ERV1:LTR	6.365862114	0.370542	0.672776	-0.30223	0.108404
MER130:DNA:DNA	4.540250432	0.391056	0.693239	-0.30218	0.108638
LTR14B:ERVK:LTR	7.504039595	0.32311	0.625066	-0.30196	0.109014
MER66D:ERV1:LTR	5.702950113	0.377482	0.679271	-0.30179	0.109155

LTR35A:ERV1:LTR	5.183223049	0.384619	0.686086	-0.30147	0.109906
MER84:ERV1:LTR	5.934998675	0.377346	0.677619	-0.30027	0.111549
MLT1A1-int:ERVL-MaLR:LTR	6.336810376	0.373347	0.673287	-0.29994	0.111925
Helitron2Na_Mam:Helitron:RC?	6.454966403	0.371246	0.670959	-0.29971	0.11216
MER67A:ERV1:LTR	6.763329268	0.362977	0.660993	-0.29802	0.114319
MER4A1_:ERV1:LTR	6.836644431	0.360622	0.657825	-0.2972	0.115681
THE1-int:ERVL-MaLR:LTR	5.35397826	0.386389	0.683502	-0.29711	0.116009
LTR82A:ERVL:LTR	7.207696593	0.342484	0.639498	-0.29701	0.116197
LTR81:Gypsy:LTR	3.934273528	0.396282	0.693139	-0.29686	0.11662
MER8:TcMar-Tigger:DNA	9.245934767	0.256601	0.552862	-0.29626	0.117418
MSTA1:ERVL-MaLR:LTR	7.225451768	0.342455	0.638589	-0.29613	0.117606
MER52C:ERV1:LTR	8.808445634	0.270836	0.566712	-0.29588	0.118075
Tigger14a:TcMar-Tigger:DNA	7.703741478	0.320844	0.616556	-0.29571	0.118263
MER4B-int:ERV1:LTR	7.81398337	0.315612	0.611306	-0.29569	0.118357
X7C_LINE:CR1:LINE	7.374986301	0.336409	0.631094	-0.29469	0.119718
LTR76:ERV1:LTR	6.298742581	0.379548	0.673902	-0.29435	0.120282
MER4D:ERV1:LTR	8.836657216	0.272205	0.565754	-0.29355	0.121221
Tigger3d:TcMar-Tigger:DNA	6.814046439	0.366205	0.658826	-0.29262	0.123192
Helitron1Na_Mam:Helitron:RC	5.186784097	0.393539	0.686034	-0.29249	0.123333
LTR27B:ERV1:LTR	6.014633923	0.384783	0.67703	-0.29225	0.123662
LTR75_1:ERV1:LTR	5.280064019	0.393063	0.684641	-0.29158	0.124977
THE1B-int:ERVL-MaLR:LTR	11.57330319	0.255171	0.546378	-0.29121	0.125634
LTR23-int:ERV1:LTR	5.243823837	0.394162	0.685188	-0.29103	0.126103
Zaphod3:hAT-Tip100:DNA	7.319311113	0.343152	0.63384	-0.29069	0.126385
MLT1G3-int:ERVL-MaLR:LTR	4.488634213	0.402915	0.69355	-0.29063	0.126432
HERV1_LTRc:ERV1:LTR	3.776490286	0.401172	0.691602	-0.29043	0.126714
LTR16A2:ERVL:LTR	7.830943056	0.320114	0.610462	-0.29035	0.127042
MER101:ERV1:LTR	6.759501587	0.370873	0.661151	-0.29028	0.12723
LTR16:ERVL:LTR	8.961206417	0.271484	0.561591	-0.29011	0.127465
LTR75B:ERVL:LTR	5.845686127	0.388899	0.678234	-0.28933	0.128357
MER31A:ERV1:LTR	9.205636611	0.264779	0.554002	-0.28922	0.128592
ERV24_Prim-int:ERV1:LTR	5.346060861	0.39464	0.683625	-0.28898	0.128967
LTR49:ERV1:LTR	7.677027285	0.330762	0.617754	-0.28699	0.132113
LTR22B:ERVK:LTR	6.102109673	0.389538	0.676281	-0.28674	0.132441
MER44A:TcMar-Tigger:DNA	9.699403824	0.256797	0.543405	-0.28661	0.132582
LTR86B1:ERVL:LTR	5.330420465	0.397546	0.683868	-0.28632	0.133052
LTR77:ERV1:LTR	3.806320384	0.405671	0.691941	-0.28627	0.133239
HERVFH19-int:ERV1:LTR	5.115682521	0.401296	0.687054	-0.28576	0.134085
Merlin1_HS:Merlin:DNA	4.778208784	0.406223	0.69122	-0.285	0.135399
Zaphod:hAT-Tip100:DNA	7.226848986	0.353981	0.638517	-0.28454	0.136244
LTR1F1:ERV1:LTR	5.105540488	0.402681	0.687196	-0.28452	0.136291
Charlie30a:hAT-Charlie:DNA	7.33039919	0.34896	0.633288	-0.28433	0.136479
L1ME5:L1:LINE	9.584849773	0.261383	0.545139	-0.28376	0.137371
MER51B:ERV1:LTR	7.188384015	0.356856	0.640488	-0.28363	0.137559
MER34A:ERV1:LTR	8.585144765	0.291081	0.574315	-0.28323	0.138404
ORSL-2a:hAT-Tip100:DNA	6.288840291	0.39109	0.674053	-0.28296	0.138732
LTR26B:ERV1:LTR	5.13380557	0.403889	0.686798	-0.28291	0.138873

LTR23:ERV1:LTR	7.801413326	0.329693	0.611926	-0.28223	0.139906
LTR62:ERVL:LTR	6.824902716	0.376327	0.658348	-0.28202	0.140141
LTR12B:ERV1:LTR	5.51011851	0.399975	0.681194	-0.28122	0.141221
MLT1L:ERVL-MaLR:LTR	11.26673278	0.261656	0.542493	-0.28084	0.141878
L1MC5:L1:LINE	12.58609988	0.286594	0.567343	-0.28075	0.141972
UCON40:UCON40:Unknown	3.66815578	0.40964	0.690183	-0.28054	0.142394
LTR70:ERV1:LTR	6.941927244	0.372662	0.652913	-0.28025	0.143052
HUERS-P1-int:ERV1:LTR	6.933303123	0.373445	0.653328	-0.27988	0.14385
Charlie1:hAT-Charlie:DNA	10.72145642	0.259187	0.538407	-0.27922	0.144836
LTR12D:ERV1:LTR	7.709503677	0.337875	0.616293	-0.27842	0.146526
LTR104_Mam:Gypsy:LTR	7.827854048	0.332379	0.610616	-0.27824	0.146714
LTR16B1:ERVL:LTR	7.597587336	0.343118	0.621109	-0.27799	0.147089
UCON107:hAT-Tag1:DNA	5.042044276	0.410352	0.688067	-0.27771	0.147653
LTR1D1:ERV1:LTR	6.172132916	0.398005	0.675566	-0.27756	0.147981
LTR24C:ERV1:LTR	6.771205786	0.383384	0.660664	-0.27728	0.148404
MARNA:TcMar-Mariner:DNA	9.724541837	0.266146	0.54306	-0.27691	0.14892
MER126:DNA:DNA	5.8530254	0.401303	0.678184	-0.27688	0.149061
PrimLTR79:ERV1:LTR	5.204439614	0.408972	0.685774	-0.2768	0.149202
UCON8:DNA:DNA	4.620303321	0.416091	0.692662	-0.27657	0.149624
MER6B:TcMar-Tigger:DNA	7.163805736	0.365667	0.641749	-0.27608	0.15061
L1PBb:L1:LINE	6.460516763	0.394904	0.670832	-0.27593	0.150892
LTR43-int:ERV1:LTR	3.89145872	0.417197	0.692784	-0.27559	0.151643
MER49:ERV1:LTR	9.527565641	0.271688	0.546169	-0.27448	0.153192
LTR73:ERV1:LTR	5.114581026	0.413428	0.68707	-0.27364	0.154695
Charlie21a:hAT-Charlie:DNA	7.829160403	0.337152	0.610551	-0.2734	0.155164
LTR60B:ERV1:LTR	4.812002979	0.417874	0.690864	-0.27299	0.155587
L1PB:L1:LINE	8.633419915	0.299757	0.572681	-0.27292	0.155681
L1ME3E:L1:LINE	10.38394889	0.265315	0.538068	-0.27275	0.155962
LTR10G:ERV1:LTR	4.892663016	0.417316	0.689954	-0.27264	0.156197
LTR28C:ERV1:LTR	7.581149161	0.349223	0.621774	-0.27255	0.156479
MER97a:hAT-Tip100:DNA	5.233085154	0.412809	0.685349	-0.27254	0.156526
Tigger7:TcMar-Tigger:DNA	9.635303624	0.272012	0.544337	-0.27233	0.157371
MamRep488:hAT-Tip100:DNA	6.351265702	0.401104	0.673037	-0.27193	0.157887
LTR38B:ERV1:LTR	5.535736469	0.409025	0.680884	-0.27186	0.158028
MER30B:hAT-Charlie:DNA	5.72230582	0.408101	0.679118	-0.27102	0.159249
MER83:ERV1:LTR	7.487376477	0.356086	0.625812	-0.26973	0.161502
LTR12E:ERV1:LTR	8.115950568	0.326219	0.595609	-0.26939	0.16216
LTR86C:ERVL:LTR	5.742145164	0.40997	0.678967	-0.269	0.162864
MER34D:ERV1:LTR	6.103436713	0.407709	0.676269	-0.26856	0.163615
AluSx:Alu:SINE	16.71556264	0.51878	0.786823	-0.26804	0.164836
MLT1C-int:ERVL-MaLR:LTR	7.513922909	0.357595	0.62463	-0.26703	0.166291
LTR72:ERV1:LTR	5.570766925	0.413652	0.680491	-0.26684	0.166667
MER20:hAT-Charlie:DNA	13.24256695	0.321094	0.58769	-0.2666	0.16723
LTR91:ERVL:LTR	5.184535397	0.419527	0.686066	-0.26654	0.167324
LTR22C2:ERVK:LTR	5.643309896	0.414026	0.679777	-0.26575	0.168732
ERV3-16A3_LTR:ERVL:LTR	7.767482543	0.348051	0.613574	-0.26552	0.169249
LTR71A:ERV1:LTR	5.687436954	0.413998	0.679397	-0.2654	0.169484

MLT1A1:ERVL-MaLR:LTR	10.51792617	0.272748	0.537976	-0.26523	0.169812
ERVL-B4-int:ERVL:LTR	9.758371393	0.277428	0.542614	-0.26519	0.169953
MER65-int:ERV1:LTR	7.851615113	0.345272	0.609423	-0.26415	0.171549
MamGypLTR3a:Gypsy:LTR	7.408137442	0.365636	0.629495	-0.26386	0.172066
MLT1M:ERVL-MaLR:LTR	9.195095826	0.290476	0.554307	-0.26383	0.17216
X7B_LINE:CR1:LINE	7.637510012	0.355723	0.619464	-0.26374	0.172347
HUERS-P3b-int:ERV1:LTR	6.218718553	0.411293	0.675015	-0.26372	0.172394
L1PA4:L1:LINE	13.06864397	0.318367	0.581777	-0.26341	0.173005
L1MC2:L1:LINE	11.13281483	0.277782	0.541152	-0.26337	0.173192
LTR79:ERVL:LTR	9.424129553	0.285015	0.548336	-0.26332	0.173286
MER131:DNA:DNA?	7.360334369	0.368709	0.63181	-0.2631	0.173615
Charlie14a:hAT-Charlie:DNA	6.08124507	0.413476	0.676472	-0.263	0.173709
L1M3de:L1:LINE	6.799768376	0.396627	0.659448	-0.26282	0.173944
LTR13:ERVK:LTR	10.90880543	0.276605	0.539389	-0.26278	0.173991
LTR71B:ERV1:LTR	6.431472149	0.409375	0.671476	-0.2621	0.175493
UCON33:TcMar-Tigger:DNA	4.048965178	0.431775	0.693857	-0.26208	0.17554
LTR27E:ERV1:LTR	6.08632944	0.414406	0.676427	-0.26202	0.175634
X6A_LINE:CR1:LINE	6.96150298	0.389965	0.651962	-0.262	0.175822
MLT1E2-int:ERVL-MaLR:LTR	5.582623153	0.418551	0.680366	-0.26182	0.17615
Kanga1d:TcMar-Tc2:DNA	6.603450322	0.405409	0.666927	-0.26152	0.176526
Tigger20a:TcMar-Tigger:DNA	8.459571093	0.318278	0.579041	-0.26076	0.177934
Charlie16a:hAT-Charlie:DNA	8.82250569	0.305491	0.566234	-0.26074	0.178075
Ricksha_c:MULE-MuDR:DNA	8.44377176	0.318962	0.579701	-0.26074	0.178122
MER63C:hAT-Blackjack:DNA	6.91238084	0.393881	0.654328	-0.26045	0.178873
LTR90B:LTR:LTR	5.936749438	0.417229	0.677607	-0.26038	0.179061
LTR87:ERVL:LTR	8.186413576	0.332228	0.591944	-0.25972	0.180376
MER110-int:ERV1:LTR	4.849986384	0.430816	0.690446	-0.25963	0.18061
LTR57-int:ERVL:LTR	5.856441971	0.418634	0.67816	-0.25953	0.180798
L1MC4:L1:LINE	13.60966099	0.341917	0.601417	-0.2595	0.180892
LTR82B:ERVL:LTR	7.191280226	0.381335	0.640339	-0.259	0.181831
SATR1:Satellite:Satellite	6.651476331	0.406469	0.665305	-0.25884	0.182394
Charlie2a:hAT-Charlie:DNA	9.051140036	0.30042	0.558686	-0.25827	0.183615
Kanga1b:TcMar-Tc2:DNA	6.586041564	0.409245	0.667478	-0.25823	0.183709
MLT2E:ERVL:LTR	6.370904771	0.415198	0.672683	-0.25749	0.184601
SATR2:Satellite:Satellite	5.028909767	0.431292	0.688242	-0.25695	0.185446
MER76:ERVL:LTR	7.542704514	0.366962	0.623382	-0.25642	0.185822
LTR34:ERV1:LTR	6.257651642	0.418198	0.674502	-0.2563	0.186009
UCON38:UCON38:Unknown	4.366438786	0.437782	0.694083	-0.2563	0.186056
Eulor9C:DNA:DNA	4.498512361	0.437411	0.693494	-0.25608	0.186526
LTR44:ERV1:LTR	5.981139792	0.421635	0.677286	-0.25565	0.187371
MER4A:ERV1:LTR	8.371972501	0.327254	0.582862	-0.25561	0.187418
LTR81C:Gypsy:LTR	5.837840372	0.422766	0.678287	-0.25552	0.1877
LTR1B:ERV1:LTR	6.247807814	0.419145	0.674637	-0.25549	0.187793
LTR54B:ERV1:LTR	8.025029548	0.345189	0.600405	-0.25522	0.188075
Helitron1Nb_Mam:Helitron:RC	4.870824846	0.435036	0.690208	-0.25517	0.188216
LTR1B0:ERV1:LTR	3.650127641	0.434981	0.689918	-0.25494	0.188685
HERVL32-int:ERVL:LTR	4.453178555	0.439258	0.693735	-0.25448	0.189296

MER53:hAT:DNA	9.280926478	0.29759	0.551904	-0.25431	0.189859
AluSz:Alu:SINE	16.59911842	0.523303	0.777588	-0.25429	0.189953
LTR81AB:Gypsy:LTR	4.206003855	0.440315	0.694291	-0.25398	0.190563
MLT2D:ERVL:LTR	9.747996948	0.288878	0.542749	-0.25387	0.190798
AluJo:Alu:SINE	15.8988881	0.472371	0.725862	-0.25349	0.191362
MER102b:hAT-Charlie:DNA	10.75850357	0.285123	0.538558	-0.25344	0.191455
HERVI-int:ERV1:LTR	5.506858164	0.428246	0.681235	-0.25299	0.192535
MER97d:hAT-Tip100:DNA	6.746755109	0.408952	0.661674	-0.25272	0.19338
hAT-N1_Mam:hAT-Tip100:DNA	6.62938962	0.414018	0.66607	-0.25205	0.194742
Charlie1b:hAT-Charlie:DNA	10.23670903	0.286833	0.538521	-0.25169	0.195493
MER9a2:ERVK:LTR	7.740840154	0.363282	0.61484	-0.25156	0.195869
HERVK14-int:ERVK:LTR	6.952825324	0.401231	0.652385	-0.25115	0.196995
LTR25:ERV1:LTR	5.796111569	0.428318	0.678577	-0.25026	0.19831
MSTC-int:ERVL-MaLR:LTR	5.4652468	0.431588	0.68179	-0.2502	0.198498
HERVIP10F-int:ERV1:LTR	6.547604789	0.418571	0.668616	-0.25004	0.198873
LTR52:ERVL:LTR	6.882584242	0.40594	0.655727	-0.24979	0.19939
LTR22:ERVK:LTR	5.115234288	0.437465	0.68706	-0.2496	0.199859
Charlie19a:hAT-Charlie:DNA	9.133125079	0.307302	0.556144	-0.24884	0.201408
LTR18A:ERVL:LTR	5.715464153	0.430354	0.679172	-0.24882	0.201596
L2:L2:LINE	14.63679061	0.40091	0.648898	-0.24799	0.20338
MLT1E2:ERVL-MaLR:LTR	10.29717708	0.29042	0.53829	-0.24787	0.203662
HAL1b:L1:LINE	10.48883152	0.290272	0.537971	-0.2477	0.203944
AluSx1:Alu:SINE	16.83327471	0.548691	0.796343	-0.24765	0.204085
MSTA:ERVL-MaLR:LTR	12.27058759	0.312685	0.559464	-0.24678	0.206338
MLT1E:ERVL-MaLR:LTR	7.877740854	0.361639	0.608095	-0.24646	0.207042
MER107:hAT-Charlie:DNA	7.828080343	0.364428	0.610605	-0.24618	0.207606
MER50:ERV1:LTR	9.474527739	0.301269	0.547234	-0.24596	0.208075
LTR43:ERV1:LTR	7.550138925	0.377268	0.623065	-0.2458	0.208216
MLT1J-int:ERVL-MaLR:LTR	7.474846275	0.381309	0.626379	-0.24507	0.209765
LTR9B:ERV1:LTR	7.763504576	0.369289	0.613765	-0.24448	0.210751
MamTip1:hAT-Tip100:DNA	7.194860401	0.395682	0.640155	-0.24447	0.210798
L1PA7:L1:LINE	13.22161449	0.342722	0.586958	-0.24424	0.211455
HSMAR2:TcMar-Mariner:DNA	9.287493835	0.30817	0.551727	-0.24356	0.212488
LTR48:ERV1:LTR	6.977786679	0.407729	0.651164	-0.24344	0.212629
SVA_B:SVA:Retroposon	11.37796395	0.300514	0.543772	-0.24326	0.213286
MER31-int:ERV1:LTR	7.870705566	0.365507	0.608454	-0.24295	0.213709
MER4E:ERV1:LTR	8.092571485	0.354177	0.596838	-0.24266	0.214507
MLT1E3-int:ERVL-MaLR:LTR	5.017942783	0.446159	0.688387	-0.24223	0.215352
AluSc8:Alu:SINE	14.76229848	0.413465	0.655626	-0.24216	0.215446
L1M4:L1:LINE	12.82814605	0.332248	0.574222	-0.24197	0.215728
MSTB:ERVL-MaLR:LTR	11.55298763	0.304132	0.546085	-0.24195	0.215822
MER83C:ERV1:LTR	4.401999883	0.452085	0.693959	-0.24187	0.216009
MER87:ERV1:LTR	5.893585215	0.436397	0.677907	-0.24151	0.216385
LTR12C:ERV1:LTR	12.53298633	0.324513	0.565931	-0.24142	0.216573
MLT1B:ERVL-MaLR:LTR	12.61639754	0.326753	0.568164	-0.24141	0.21662
LTR12_:ERV1:LTR	7.178951496	0.399799	0.640972	-0.24117	0.21723
Tigger3b:TcMar-Tigger:DNA	11.36231083	0.302948	0.543583	-0.24064	0.217981

MER103C:hAT-Charlie:DNA	11.21061659	0.30212	0.541905	-0.23978	0.219718
MER57E3:ERV1:LTR	8.375616221	0.342989	0.582696	-0.23971	0.219859
MER66A:ERV1:LTR	6.251647711	0.435145	0.674585	-0.23944	0.220423
LTR8:ERV1:LTR	10.60627577	0.298783	0.53808	-0.2393	0.220798
PRIMA4_LTR:ERV1:LTR	6.467652538	0.4316	0.670667	-0.23907	0.220986
MamRep4096:hAT-Tip100:DNA	7.661234349	0.379748	0.618447	-0.2387	0.221408
MER11D:ERVK:LTR	6.831562942	0.419498	0.658052	-0.23855	0.221643
MER65A:ERV1:LTR	8.155696409	0.35521	0.593533	-0.23832	0.221925
HSMAR1:TcMar-Mariner:DNA	8.483593078	0.339841	0.578062	-0.23822	0.222254
MER52D:ERV1:LTR	8.928913922	0.325117	0.562657	-0.23754	0.223803
Charlie18a:hAT-Charlie:DNA	9.184548715	0.317248	0.554614	-0.23737	0.224413
MER87B:ERV1:LTR	6.565363626	0.430943	0.668103	-0.23716	0.224789
FLAM_A:Alu:SINE	12.50480885	0.328392	0.565196	-0.2368	0.225587
MER4E1:ERV1:LTR	9.334421762	0.313761	0.550501	-0.23674	0.225634
CR1-16_AMi:CR1:LINE	5.553305166	0.44399	0.680683	-0.23669	0.225822
MLT1J:ERVL-MaLR:LTR	12.02522097	0.318001	0.554182	-0.23618	0.227042
MER31B:ERV1:LTR	8.836097235	0.329777	0.565773	-0.236	0.227183
L1MC5a:L1:LINE	12.74915882	0.336124	0.571897	-0.23577	0.227559
LTR8B:ERV1:LTR	8.654654499	0.33644	0.571959	-0.23552	0.227934
L1ME3A:L1:LINE	12.26938308	0.32415	0.559436	-0.23529	0.228263
MER101B:ERV1:LTR	5.09921583	0.452014	0.687285	-0.23527	0.22831
AmnSINE1:5S-Deu-L2:SINE	9.270972636	0.316915	0.552173	-0.23526	0.228357
MLT1G1-int:ERVL-MaLR:LTR	5.264084432	0.449818	0.684883	-0.23507	0.228779
LTR16E1:ERVL:LTR	8.484628752	0.343096	0.578021	-0.23493	0.228873
Plat_L3:CR1:LINE	9.675888813	0.308935	0.543738	-0.2348	0.229249
L1PA5:L1:LINE	13.05377053	0.347071	0.581289	-0.23422	0.230235
MER58C:hAT-Charlie:DNA	9.932357717	0.306505	0.540652	-0.23415	0.230329
MER33:hAT-Charlie:DNA	11.45944855	0.310726	0.544803	-0.23408	0.230516
LTR85b:Gypsy:LTR	6.790103216	0.426283	0.659863	-0.23358	0.231127
MER65C:ERV1:LTR	7.711590329	0.383019	0.616197	-0.23318	0.232019
HERV16-int:ERVL:LTR	8.300138307	0.353151	0.586247	-0.2331	0.232066
MER45B:hAT-Tip100:DNA	7.768961943	0.380412	0.613503	-0.23309	0.232113
Ricksha_b:MULE-MuDR:DNA	4.783716796	0.458221	0.691163	-0.23294	0.232254
Arthur1:hAT-Tip100:DNA	8.376853349	0.349743	0.58264	-0.2329	0.232441
MER61E:ERV1:LTR	5.588541947	0.447459	0.680305	-0.23285	0.232582
Charlie23a:hAT-Charlie:DNA	8.532219954	0.343637	0.576185	-0.23255	0.233146
Charlie13b:hAT-Charlie:DNA	7.000592381	0.417575	0.650037	-0.23246	0.233333
MER68C:ERVL:LTR	4.226209006	0.463327	0.694301	-0.23097	0.236291
LTR14A:ERVK:LTR	5.791151997	0.447699	0.678612	-0.23091	0.236432
Tigger12:TcMar-Tigger:DNA	7.838578145	0.379257	0.61008	-0.23082	0.236573
X9_LINE:L1:LINE	7.328510563	0.402809	0.633382	-0.23057	0.237653
AluJb:Alu:SINE	16.65828596	0.551733	0.782258	-0.23053	0.237887
Charlie3:hAT-Charlie:DNA	7.574565165	0.391742	0.622044	-0.2303	0.238451
HAL1ME:L1:LINE	10.99548629	0.309764	0.54	-0.23024	0.238592
MLT1N2:ERVL-MaLR:LTR	9.786831253	0.312713	0.542255	-0.22954	0.240141
MER74A:ERVL:LTR	8.955450236	0.332496	0.56178	-0.22928	0.240845
LTR1F2:ERV1:LTR	6.018731755	0.448587	0.676998	-0.22841	0.242066

MamRep564:MamRep564:Unknown	7.850593571	0.38117	0.609475	-0.2283	0.242207
LTR21B:ERV1:LTR	4.427716014	0.465646	0.693853	-0.22821	0.242488
L1MEg:L1:LINE	12.34101562	0.333487	0.561116	-0.22763	0.243521
LTR85a:Gypsy:LTR	7.973413302	0.375661	0.603126	-0.22746	0.243803
Eulor2B:DNA:DNA?	4.314883039	0.466888	0.694217	-0.22733	0.244038
MSTB1-int:ERVL-MaLR:LTR	5.445384376	0.454807	0.682076	-0.22727	0.244178
Tigger12c:TcMar-Tigger:DNA	7.775354449	0.386078	0.613195	-0.22712	0.244319
UCON1:UCON1:Unknown	4.045388699	0.46696	0.69384	-0.22688	0.244977
L1MB8:L1:LINE	12.84191734	0.348212	0.574635	-0.22642	0.245634
LTR1E:ERV1:LTR	5.385595013	0.457167	0.683007	-0.22584	0.246573
MER21-int:ERVL:LTR	8.105979995	0.370631	0.596133	-0.2255	0.24723
AluYh7:Alu:SINE	4.531264688	0.468275	0.693296	-0.22502	0.248216
LTR86A1:ERVL:LTR	7.277421532	0.410976	0.635945	-0.22497	0.248404
MER57B2:ERV1:LTR	7.265794601	0.411597	0.636534	-0.22494	0.248498
MER34C2:ERV1:LTR	7.616205327	0.395551	0.620353	-0.2248	0.248732
LTR40b:ERVL:LTR	8.436887525	0.355213	0.579993	-0.22478	0.248826
MER110A:ERV1:LTR	7.076605995	0.421471	0.646211	-0.22474	0.24892
MLT2B5:ERVL:LTR	5.344622802	0.458917	0.683647	-0.22473	0.248967
MLT2B3:ERVL:LTR	9.8146885	0.317342	0.541919	-0.22458	0.249202
GSAT:centr:Satellite	3.342616834	0.459758	0.684161	-0.2244	0.249859
LTR41C:ERVL:LTR	7.733095248	0.391078	0.615203	-0.22412	0.250188
MER106A:hAT-Charlie:DNA	7.210306138	0.415645	0.639364	-0.22372	0.251033
MER77B:ERVL:LTR	9.20089408	0.330787	0.554139	-0.22335	0.251737
Cheshire:hAT-Charlie:DNA	8.693087172	0.347324	0.570649	-0.22332	0.251831
L1PA6:L1:LINE	12.47766837	0.341477	0.564497	-0.22302	0.252629
LTR26C:ERV1:LTR	6.897072433	0.432041	0.65505	-0.22301	0.252676
LTR33C:ERVL:LTR	7.735098825	0.392239	0.615109	-0.22287	0.253005
LTR88a:Gypsy:LTR	6.471062251	0.447789	0.670587	-0.2228	0.253333
MER97c:hAT-Tip100:DNA	4.417749145	0.471902	0.693895	-0.22199	0.254648
Charlie10a:hAT-Charlie:DNA	6.421057522	0.449915	0.671697	-0.22178	0.255164
MSTD:ERVL-MaLR:LTR	10.9318259	0.317885	0.539543	-0.22166	0.255399
MamRep1527:LTR:LTR	6.862487	0.43512	0.656654	-0.22153	0.255822
MER5B:hAT-Charlie:DNA	12.65465703	0.348184	0.569218	-0.22103	0.256854
MER74C:ERVL:LTR	4.015633629	0.473184	0.693683	-0.2205	0.258498
MLT1I:ERVL-MaLR:LTR	10.76009415	0.318129	0.538565	-0.22044	0.258592
UCON29:PiggyBac:DNA	5.390784618	0.462659	0.682925	-0.22027	0.259249
L1MA9:L1:LINE	11.71601809	0.328549	0.548574	-0.22002	0.259859
LTR41B:ERVL:LTR	8.143314678	0.374197	0.594178	-0.21998	0.259953
MER58D:hAT-Charlie:DNA	7.740844961	0.394918	0.61484	-0.21992	0.260141
MLT1K-int:ERVL-MaLR:LTR	4.759203107	0.471749	0.691413	-0.21966	0.26061
HERVL40-int:ERVL:LTR	8.293429174	0.367008	0.586573	-0.21956	0.260704
MLT1O:ERVL-MaLR:LTR	9.900837193	0.32148	0.540966	-0.21949	0.260845
AluYk12:Alu:SINE	6.858354368	0.437458	0.656843	-0.21939	0.26108
LTR35B:ERV1:LTR	5.365050247	0.464042	0.683329	-0.21929	0.261455
L1P4b:L1:LINE	4.010921064	0.474618	0.693656	-0.21904	0.262207
Charlie5:hAT-Charlie:DNA	9.130129738	0.338255	0.556235	-0.21798	0.263615
MER34:ERV1:LTR	8.350176921	0.365904	0.583868	-0.21796	0.263756

LTR16B2:ERVL:LTR	8.221889269	0.3726	0.590131	-0.21753	0.264695
MLT1G:ERVL-MaLR:LTR	9.798536179	0.324633	0.542112	-0.21748	0.264883
MER41G:ERV1:LTR	4.347455034	0.476832	0.694139	-0.21731	0.265352
MER11A:ERVK:LTR	9.17960846	0.337472	0.554758	-0.21729	0.265399
MamGypLTR1c:Gypsy:LTR	6.992919795	0.433301	0.650417	-0.21712	0.265681
LTR64:ERV1:LTR	6.393391121	0.455368	0.672256	-0.21689	0.266197
MER89:ERV1:LTR	8.378144357	0.366131	0.582581	-0.21645	0.266948
LTR67B:ERVL:LTR	9.537047351	0.32975	0.54599	-0.21624	0.267418
MER58B:hAT-Charlie:DNA	11.35863581	0.327642	0.543539	-0.2159	0.267887
MER104:TcMar-Tc2:DNA	9.08151592	0.342148	0.557731	-0.21558	0.268685
MLT1D-int:ERVL-MaLR:LTR	7.786268597	0.397184	0.612666	-0.21548	0.268967
Tigger19b:TcMar-Tigger:DNA	10.3259898	0.322751	0.538202	-0.21545	0.269061
LTR107_Mam:LTR:LTR	5.682114698	0.464118	0.679441	-0.21532	0.269249
MER5A:hAT-Charlie:DNA	13.36239828	0.377249	0.591985	-0.21474	0.270329
MER5A1:hAT-Charlie:DNA	11.99859955	0.339198	0.553654	-0.21446	0.270751
LTR50:ERVL:LTR	9.127687175	0.341973	0.556309	-0.21434	0.271268
L1MC4a:L1:LINE	12.41283389	0.348973	0.562864	-0.21389	0.271831
AluSp:Alu:SINE	15.77999712	0.503881	0.717724	-0.21384	0.271972
LOR1b:ERV1:LTR	7.577460597	0.408413	0.621925	-0.21351	0.272535
MER4A1:ERV1:LTR	9.23974347	0.339643	0.553035	-0.21339	0.27277
CR1-L3A_Croc:CR1:LINE	5.320941657	0.47079	0.684014	-0.21322	0.273286
L1PB4:L1:LINE	11.04377931	0.327643	0.540379	-0.21274	0.274366
MER57-int:ERV1:LTR	7.838518737	0.397712	0.610083	-0.21237	0.274883
L1MB3:L1:LINE	13.01122041	0.368054	0.579907	-0.21185	0.275915
HERVKC4-int:ERVK:LTR	4.442840356	0.482548	0.693784	-0.21124	0.27723
Charlie10b:hAT-Charlie:DNA	6.611787931	0.455493	0.666657	-0.21116	0.277512
MER58A:hAT-Charlie:DNA	12.25035529	0.347982	0.559001	-0.21102	0.27784
Arthur1A:hAT-Tip100:DNA	8.32781112	0.374023	0.58492	-0.2109	0.278075
LTR43B:ERV1:LTR	4.934857781	0.478672	0.689445	-0.21077	0.278216
Charlie4z:hAT-Charlie:DNA	11.16391136	0.33087	0.541444	-0.21057	0.278451
CR1_Mam:CR1:LINE	8.88309062	0.354288	0.564188	-0.2099	0.279718
L1PREC2:L1:LINE	11.64095944	0.337989	0.547388	-0.2094	0.28108
Charlie13a:hAT-Charlie:DNA	7.908131669	0.397156	0.606532	-0.20938	0.281127
MER54B:ERVL:LTR	6.615329259	0.458248	0.66654	-0.20829	0.283333
MER39:ERV1:LTR	9.742247505	0.334875	0.542824	-0.20795	0.284038
CER:Satellite:Satellite	4.444121176	0.485902	0.693778	-0.20788	0.284319
MER83B-int:ERV1:LTR	5.757275323	0.471064	0.678855	-0.20779	0.284507
MER3:hAT-Charlie:DNA	11.74545731	0.341404	0.549058	-0.20765	0.284742
MER2:TcMar-Tigger:DNA	11.48657611	0.337745	0.545164	-0.20742	0.285446
MLT2A1:ERVL:LTR	9.704045524	0.336746	0.54334	-0.20659	0.287136
L1PA11:L1:LINE	10.91038718	0.332809	0.539399	-0.20659	0.287277
L1M4c:L1:LINE	10.86362295	0.333578	0.539106	-0.20553	0.289484
LTR1A1:ERV1:LTR	7.045455267	0.442345	0.647789	-0.20544	0.289718
L1PA8A:L1:LINE	9.995106498	0.334854	0.540078	-0.20522	0.290141
LTR10A:ERV1:LTR	7.213592625	0.43411	0.639196	-0.20509	0.290282
LTR40a:ERVL:LTR	8.285222433	0.382725	0.586973	-0.20425	0.291972
AluYb9:Alu:SINE	7.420125113	0.424769	0.628924	-0.20415	0.292347

MER105:hAT-Charlie:DNA	8.423786393	0.376423	0.580556	-0.20413	0.292394
L1ME3:L1:LINE	11.79731065	0.345939	0.549936	-0.204	0.29277
L1MEg2:L1:LINE	7.159167705	0.43835	0.641987	-0.20364	0.293709
L1MC:L1:LINE	10.80709532	0.335313	0.53879	-0.20348	0.293897
L3b:CR1:LINE	11.36470269	0.340516	0.543611	-0.2031	0.294601
L1PB1:L1:LINE	12.75575556	0.36901	0.572088	-0.20308	0.294695
MLT1E1A:ERVL-MaLR:LTR	9.41807828	0.345523	0.548475	-0.20295	0.294977
MER50B:ERV1:LTR	7.459577912	0.424404	0.627079	-0.20268	0.295728
MLT2B1:ERVL:LTR	9.52359296	0.343987	0.546245	-0.20226	0.296526
Helitron3Na_Mam:Helitron:RC	7.939089254	0.402723	0.604924	-0.2022	0.296761
Tigger12A:TcMar-Tigger:DNA	7.161951193	0.44006	0.641844	-0.20178	0.297465
THE1B:ERVL-MaLR:LTR	12.16109618	0.355247	0.557016	-0.20177	0.297606
ERVL-E-int:ERVL:LTR	10.44614615	0.336386	0.537988	-0.2016	0.297793
MER9a3:ERVK:LTR	7.959574424	0.40234	0.603852	-0.20151	0.297981
LTR24:ERV1:LTR	6.597329136	0.465724	0.667123	-0.2014	0.298216
L1PA13:L1:LINE	11.52444583	0.344316	0.545683	-0.20137	0.298263
MER102c:hAT-Charlie:DNA	10.28939078	0.337141	0.538316	-0.20118	0.298732
Tigger2b_Pri:TcMar-Tigger:DNA	9.138530849	0.355309	0.555981	-0.20067	0.300047
MER113A:hAT-Charlie:DNA	8.703796179	0.369892	0.570283	-0.20039	0.300563
HERVP71A-int:ERV1:LTR	7.157608809	0.442053	0.642067	-0.20001	0.301221
Kanga2_a:TcMar-Tc2:DNA	8.887256131	0.364075	0.564048	-0.19997	0.301268
L1M6:L1:LINE	9.959108385	0.340451	0.540399	-0.19995	0.301455
PRIMA4-int:ERV1:LTR	8.056106163	0.398842	0.598763	-0.19992	0.301549
MER39B:ERV1:LTR	8.868043914	0.365024	0.564694	-0.19967	0.301972
Alu:Alu:SINE	9.850952744	0.341985	0.541501	-0.19952	0.30216
MER67D:ERV1:LTR	6.572485385	0.46866	0.667891	-0.19923	0.303146
MER117:hAT-Charlie:DNA	10.23038228	0.339796	0.538548	-0.19875	0.303991
MLT1G3:ERVL-MaLR:LTR	9.546425591	0.347232	0.545816	-0.19858	0.304225
L3:CR1:LINE	13.91697178	0.415809	0.614216	-0.19841	0.304742
L1PA3:L1:LINE	13.56986903	0.401445	0.599847	-0.1984	0.304789
LTR78:ERV1:LTR	9.998791501	0.341934	0.540047	-0.19811	0.305305
UCON20:UCON20:Unknown	3.879569847	0.494748	0.692677	-0.19793	0.305775
LTR2752:ERV1:LTR	6.584731457	0.469733	0.667518	-0.19779	0.30615
LTR5A:ERVK:LTR	8.876988436	0.366636	0.564393	-0.19776	0.306291
MER5C:hAT-Charlie:DNA	8.350681554	0.386253	0.583844	-0.19759	0.306761
Charlie8:hAT-Charlie:DNA	10.18977644	0.341391	0.538743	-0.19735	0.307371
MER57C2:ERV1:LTR	7.015461419	0.452021	0.649296	-0.19727	0.307559
L1ME2:L1:LINE	12.18582285	0.360651	0.557556	-0.1969	0.308075
BLACKJACK:hAT-Blackjack:DNA	8.214162035	0.393969	0.590524	-0.19656	0.308451
MER6:TcMar-Tigger:DNA	8.458491313	0.382735	0.579085	-0.19635	0.308967
MER51E:ERV1:LTR	5.711742131	0.482957	0.679201	-0.19624	0.309108
LTR80B:ERVL:LTR	5.134890386	0.49091	0.686782	-0.19587	0.309671
MIR1_Amn:MIR:SINE	12.96822683	0.383223	0.578535	-0.19531	0.310704
AluSq2:Alu:SINE	15.88157059	0.529816	0.724665	-0.19485	0.311737
L1P3b:L1:LINE	3.71510483	0.49605	0.690834	-0.19478	0.311831
LTR54:ERV1:LTR	8.549212953	0.380866	0.575565	-0.1947	0.312019
MLT1E3:ERVL-MaLR:LTR	9.072396791	0.363341	0.558016	-0.19467	0.312113

MER85:PiggyBac:DNA	7.563911709	0.427846	0.622486	-0.19464	0.312254
AluSc:Alu:SINE	14.86859598	0.466947	0.661482	-0.19453	0.312394
MER11B:ERVK:LTR	9.281312149	0.357494	0.551893	-0.1944	0.312582
AluYk4:Alu:SINE	9.843225149	0.347494	0.541588	-0.19409	0.313474
MER90a:ERV1:LTR	8.658604037	0.378473	0.571825	-0.19335	0.315117
MLT1J1:ERVL-MaLR:LTR	9.187937063	0.361204	0.554515	-0.19331	0.315258
LTR51:ERV1:LTR	6.99963031	0.456994	0.650084	-0.19309	0.315728
LTR37B:ERV1:LTR	8.262399611	0.395263	0.588098	-0.19284	0.316338
MER106B:hAT-Charlie:DNA	7.988602138	0.41006	0.602327	-0.19227	0.317418
MER41B:ERV1:LTR	9.828046881	0.349704	0.541762	-0.19206	0.317981
L1MC1:L1:LINE	12.4482512	0.372354	0.56375	-0.1914	0.318873
MER34B:ERV1:LTR	7.586023887	0.430551	0.621575	-0.19102	0.319437
MER115:hAT-Tip100:DNA	9.798495999	0.352806	0.542113	-0.18931	0.322864
LTR85c:Gypsy:LTR	7.381892592	0.441718	0.630758	-0.18904	0.323427
MLT1G1:ERVL-MaLR:LTR	9.792245517	0.35319	0.542189	-0.189	0.323662
MER83A-int:ERV1:LTR	4.378301719	0.505462	0.694045	-0.18858	0.324648
MER73:ERVL:LTR	5.927875118	0.489323	0.677669	-0.18835	0.325352
AluYj4:Alu:SINE	11.10635982	0.352959	0.540912	-0.18795	0.326244
MamTip2:hAT-Tip100:DNA	10.29545191	0.350632	0.538296	-0.18766	0.327089
MER119:hAT-Charlie:DNA	8.3408579	0.396705	0.584304	-0.1876	0.327183
MER61C:ERV1:LTR	5.742669211	0.49188	0.678963	-0.18708	0.328122
LTR28B:ERV1:LTR	6.769443258	0.473688	0.660738	-0.18705	0.328169
L1ME4c:L1:LINE	11.50667639	0.35843	0.545437	-0.18701	0.328216
MER91B:hAT-Tip100:DNA	8.502208508	0.390858	0.577327	-0.18647	0.329061
MER45R:hAT-Tip100:DNA	6.54857303	0.482211	0.668588	-0.18638	0.329249
L1ME4a:L1:LINE	13.38008421	0.406305	0.592634	-0.18633	0.329624
Tigger1:TcMar-Tigger:DNA	13.10230732	0.396816	0.582892	-0.18608	0.329859
MER2B:TcMar-Tigger:DNA	8.704774799	0.384596	0.57025	-0.18565	0.33061
Charlie7a:hAT-Charlie:DNA	8.711015779	0.384555	0.570036	-0.18548	0.330845
Charlie17a:hAT-Charlie:DNA	8.521626844	0.391389	0.576582	-0.18519	0.331268
AluSq:Alu:SINE	14.18112313	0.441619	0.626174	-0.18456	0.33216
L1MA8:L1:LINE	11.15777781	0.357048	0.541386	-0.18434	0.332535
L1MDa:L1:LINE	11.21670195	0.357676	0.541967	-0.18429	0.332629
L1MA7:L1:LINE	11.09971106	0.356666	0.540854	-0.18419	0.332958
MER57A-int:ERV1:LTR	7.932420991	0.421277	0.605271	-0.18399	0.333286
MER57E1:ERV1:LTR	6.685569561	0.480201	0.664065	-0.18386	0.333568
LTR33A:ERVL:LTR	8.872700118	0.380888	0.564537	-0.18365	0.333897
LTR16B:ERVL:LTR	7.321915421	0.450203	0.63371	-0.18351	0.334272
MLT1F2-int:ERVL-MaLR:LTR	5.860551891	0.494638	0.678132	-0.18349	0.334366
AluYc:Alu:SINE	11.51392541	0.362104	0.545537	-0.18343	0.334601
LTR47B3:ERVL:LTR	4.464645985	0.511178	0.693678	-0.1825	0.336667
AluYi6_4d:Alu:SINE	6.096254881	0.493923	0.676336	-0.18241	0.336901
LTR84b:ERVL:LTR	7.803744244	0.430163	0.611811	-0.18165	0.338216
AluYb8:Alu:SINE	11.17999975	0.360726	0.5416	-0.18087	0.339531
Eulor9A:DNA:DNA	4.300588466	0.513438	0.694244	-0.18081	0.339624
MER52A:ERV1:LTR	10.21585511	0.357844	0.538615	-0.18077	0.339671
MLT1A:ERVL-MaLR:LTR	10.80098205	0.35835	0.538759	-0.18041	0.340376

Charlie15a:hAT-Charlie:DNA	10.09162208	0.359172	0.539333	-0.18016	0.340939
LTR83:ERVL:LTR	6.731086293	0.482555	0.662306	-0.17975	0.34169
MamRep1894:hAT:DNA	5.740745462	0.499334	0.678978	-0.17964	0.341972
LTR17:ERV1:LTR	8.552033469	0.3959	0.575463	-0.17956	0.342113
MLT1A0-int:ERVL-MaLR:LTR	7.410627188	0.449825	0.629376	-0.17955	0.34216
MER94:hAT-Blackjack:DNA	10.10347199	0.359997	0.539253	-0.17926	0.342817
MLT1J2:ERVL-MaLR:LTR	9.936532301	0.361636	0.540611	-0.17898	0.343521
MER4B:ERV1:LTR	8.84300755	0.386565	0.565539	-0.17897	0.343568
L1MA4:L1:LINE	11.77658977	0.370645	0.549581	-0.17894	0.343662
LTR49-int:ERV1:LTR	8.751502528	0.391307	0.568653	-0.17735	0.346761
MSTB-int:ERVL-MaLR:LTR	8.347174518	0.407712	0.584008	-0.1763	0.348826
Tigger19a:TcMar-Tigger:DNA	10.47919383	0.361777	0.537972	-0.17619	0.349108
AluSg:Alu:SINE	15.23113801	0.506683	0.682558	-0.17588	0.349531
LTR1C3:ERV1:LTR	3.789722612	0.515992	0.691755	-0.17576	0.349765
AluSx3:Alu:SINE	15.01594253	0.494219	0.669842	-0.17562	0.350094
MER4CL34:ERV1:LTR	7.682825552	0.441883	0.617497	-0.17561	0.350188
Chap1_Mam:hAT-Charlie:DNA	8.452282597	0.404179	0.579344	-0.17516	0.351455
AluJr:Alu:SINE	15.94251251	0.55413	0.728895	-0.17476	0.351925
LTR12F:ERV1:LTR	7.759460844	0.440398	0.613958	-0.17356	0.353709
L1M2c:L1:LINE	7.70786396	0.443164	0.616368	-0.1732	0.354648
LTR3B_:ERVK:LTR	6.89882456	0.481772	0.654968	-0.1732	0.354695
MLT1F2:ERVL-MaLR:LTR	10.78288494	0.365478	0.53867	-0.17319	0.354742
LTR48B:ERV1:LTR	8.343263815	0.411358	0.584191	-0.17283	0.355493
Tigger3:TcMar-Tigger:DNA	8.923124718	0.390227	0.56285	-0.17262	0.356009
Tigger4:TcMar-Tigger:DNA	8.920419197	0.390544	0.56294	-0.1724	0.356385
L1P5:L1:LINE	6.055520634	0.504453	0.676697	-0.17224	0.356714
MamGypLTR1b:Gypsy:LTR	7.342395581	0.460595	0.632693	-0.1721	0.357136
LTR45C:ERV1:LTR	7.835619744	0.438133	0.610228	-0.17209	0.357183
LTR41:ERVL:LTR	8.859825739	0.392994	0.564971	-0.17198	0.357559
TAR1:telo:Satellite	7.542262165	0.452046	0.623401	-0.17135	0.358685
LTR5B:ERVK:LTR	10.09919792	0.36802	0.539281	-0.17126	0.358826
MLT2C1:ERVL:LTR	8.558532682	0.403997	0.575232	-0.17124	0.358873
LTR16E2:ERVL:LTR	8.435281652	0.409141	0.580062	-0.17092	0.359343
THE1D:ERVL-MaLR:LTR	11.11285102	0.37062	0.54097	-0.17035	0.360798
LTR40c:ERVL:LTR	8.498021469	0.407278	0.57749	-0.17021	0.361268
L1ME3G:L1:LINE	12.37491241	0.391803	0.561934	-0.17013	0.361362
MLT1H:ERVL-MaLR:LTR	11.1068653	0.370993	0.540917	-0.16992	0.361596
THE1A:ERVL-MaLR:LTR	9.668331294	0.374304	0.543847	-0.16954	0.362207
MER70B:ERVL:LTR	5.466590791	0.512397	0.681771	-0.16937	0.362582
AluYa8:Alu:SINE	6.590993421	0.498269	0.667323	-0.16905	0.36338
LTR19A:ERV1:LTR	8.578252719	0.406236	0.574548	-0.16831	0.364883
MSTA-int:ERVL-MaLR:LTR	10.2832476	0.370076	0.538337	-0.16826	0.365023
Charlie1a:hAT-Charlie:DNA	11.71268878	0.38027	0.54852	-0.16825	0.36507
L1PA8:L1:LINE	11.55650071	0.378	0.546136	-0.16814	0.365164
MER57D:ERV1:LTR	6.656161862	0.497074	0.665139	-0.16807	0.365352
LTR61:ERV1:LTR	6.65574249	0.497347	0.665154	-0.16781	0.365728
LTR1B1:ERV1:LTR	5.443813189	0.514456	0.682099	-0.16764	0.366009

SVA_C:SVA:Retroposon	11.65350182	0.380144	0.547582	-0.16744	0.366432
THE1C:ERVL-MaLR:LTR	10.77732909	0.371436	0.538643	-0.16721	0.366808
Tigger4b:TcMar-Tigger:DNA	9.723536281	0.376275	0.543073	-0.1668	0.367793
L1ME3B:L1:LINE	11.41201683	0.377737	0.544193	-0.16646	0.368404
LTR33:ERVL:LTR	11.0401087	0.373937	0.54035	-0.16641	0.368498
MER6A:TcMar-Tigger:DNA	8.788820664	0.400995	0.56738	-0.16638	0.368592
Tigger18a:TcMar-Tigger:DNA	8.947332052	0.396114	0.562048	-0.16593	0.369202
L1MB4:L1:LINE	11.88140211	0.386243	0.551429	-0.16519	0.37108
AluSg7:Alu:SINE	13.10219104	0.418653	0.582888	-0.16423	0.373333
MER4-int:ERV1:LTR	9.032099077	0.395081	0.559292	-0.16421	0.37338
Tigger2a:TcMar-Tigger:DNA	10.2627698	0.37515	0.538413	-0.16326	0.375258
Tigger1a_Art:TcMar-Tigger:DNA	6.30725699	0.510615	0.67377	-0.16315	0.375587
Charlie4a:hAT-Charlie:DNA	10.0710059	0.376396	0.539478	-0.16308	0.375728
MER54A:ERVL:LTR	8.882277117	0.401292	0.564215	-0.16292	0.375962
LOR1-int:ERV1:LTR	7.955284892	0.441362	0.604077	-0.16272	0.37615
LTR13A:ERVK:LTR	9.311441092	0.388584	0.551094	-0.16251	0.376573
L1M7:L1:LINE	9.802954716	0.379772	0.542059	-0.16229	0.377136
MamRep38:hAT:DNA	9.478088564	0.385281	0.547159	-0.16188	0.377746
Tigger17a:TcMar-Tigger:DNA	10.03678383	0.378399	0.539736	-0.16134	0.378826
MER45C:hAT-Tip100:DNA	8.075525416	0.436769	0.597737	-0.16097	0.379437
MER68:ERVL:LTR	10.34801745	0.377264	0.538145	-0.16088	0.379577
MER46C:TcMar-Tigger:DNA	10.23666868	0.377876	0.538521	-0.16064	0.380094
LTR55:ERVL:LTR	6.466412447	0.510342	0.670696	-0.16035	0.380704
HERVK3-int:ERVK:LTR	8.993053176	0.400274	0.56055	-0.16028	0.380939
L1M3a:L1:LINE	7.757168661	0.454035	0.614067	-0.16003	0.381408
L1P4d:L1:LINE	4.32638489	0.534273	0.694192	-0.15992	0.381737
L1MB2:L1:LINE	11.93341842	0.392626	0.552396	-0.15977	0.381925
AluYg6:Alu:SINE	8.808906729	0.407131	0.566696	-0.15957	0.382019
PABL_B:ERV1:LTR	6.769234494	0.501433	0.660746	-0.15931	0.382582
L1PA14:L1:LINE	10.45128589	0.378978	0.537984	-0.15901	0.38338
LTR47B:ERVL:LTR	4.324331693	0.53536	0.694196	-0.15884	0.383803
L1MEf:L1:LINE	12.11810426	0.397321	0.556095	-0.15877	0.383991
AluYd8:Alu:SINE	7.253041436	0.478454	0.637182	-0.15873	0.384272
LTR15:ERV1:LTR	6.542595664	0.510153	0.668756	-0.1586	0.384601
HERV9N-int:ERV1:LTR	7.538533288	0.465428	0.62356	-0.15813	0.385352
LTR35:ERV1:LTR	5.666595014	0.521634	0.679572	-0.15794	0.385775
L1PA16:L1:LINE	12.15209177	0.399576	0.556821	-0.15725	0.38662
LTR8A:ERV1:LTR	8.681749716	0.414072	0.571036	-0.15696	0.387277
LTR16C:ERVL:LTR	10.80481839	0.381946	0.538778	-0.15683	0.387418
ERVL-int:ERVL:LTR	6.802863258	0.502508	0.659314	-0.15681	0.387465
LTR4:ERV1:LTR	7.000138154	0.493384	0.650059	-0.15668	0.387981
LTR89:ERVL:LTR	7.610598903	0.464075	0.620583	-0.15651	0.388498
Tigger15a:TcMar-Tigger:DNA	11.21957936	0.385538	0.541997	-0.15646	0.388545
LTR10C:ERV1:LTR	8.243207397	0.433637	0.589056	-0.15542	0.390235
LTR27D:ERV1:LTR	4.563407104	0.537738	0.693083	-0.15535	0.390469
LTR27C:ERV1:LTR	6.711652402	0.507728	0.663071	-0.15534	0.390516
MLT1K:ERVL-MaLR:LTR	12.49063415	0.409501	0.56483	-0.15533	0.390563

MLT1H2:ERVL-MaLR:LTR	9.857746627	0.386509	0.541426	-0.15492	0.391408
LTR12:ERV1:LTR	8.557896429	0.42054	0.575255	-0.15471	0.391643
MLT1H2-int:ERVL-MaLR:LTR	5.498571363	0.526669	0.681341	-0.15467	0.391878
Ricksha:MULE-MuDR:DNA	6.674480539	0.509841	0.664476	-0.15463	0.392019
SVA_A:SVA:Retroposon	12.07890536	0.400862	0.555275	-0.15441	0.392441
UCON26:UCON26:Unknown	6.026737117	0.522848	0.676934	-0.15409	0.392723
L1PA17:L1:LINE	10.53327167	0.384148	0.537985	-0.15384	0.393239
L1MCa:L1:LINE	11.27971287	0.390263	0.542635	-0.15237	0.395352
MamRTE1:RTE-Bo.B:LINE	11.17746674	0.389401	0.541575	-0.15217	0.395775
L1MB7:L1:LINE	13.64110176	0.45064	0.602672	-0.15203	0.39615
Charlie6:hAT-Charlie:DNA	6.799161938	0.507723	0.659474	-0.15175	0.396526
MER124:DNA:DNA?	7.832403315	0.458969	0.610389	-0.15142	0.397042
ERV3-16A3_I-int:ERVL:LTR	10.65334563	0.386818	0.538187	-0.15137	0.397183
MER51-int:ERV1:LTR	6.788218442	0.508674	0.659944	-0.15127	0.397418
L1MD2:L1:LINE	12.07647589	0.404103	0.555225	-0.15112	0.39784
L1M4b:L1:LINE	10.63144319	0.387037	0.538133	-0.1511	0.397887
Tigger9a:TcMar-Tigger:DNA	8.17786019	0.442178	0.592385	-0.15021	0.399812
MSTB2-int:ERVL-MaLR:LTR	5.19694971	0.535916	0.685885	-0.14997	0.400376
MER112:hAT-Charlie:DNA	10.26944078	0.388488	0.538388	-0.1499	0.400516
MLT1F-int:ERVL-MaLR:LTR	7.094260011	0.495932	0.645312	-0.14938	0.401455
MLT1E1:ERVL-MaLR:LTR	8.936531801	0.413063	0.562405	-0.14934	0.401502
MER34-int:ERV1:LTR	5.953678146	0.52815	0.677486	-0.14934	0.401549
L4_B_Mam:RTE-X:LINE	9.840322373	0.392299	0.541621	-0.14932	0.401596
AluYm1:Alu:SINE	11.88940328	0.402336	0.551575	-0.14924	0.401643
Charlie2b:hAT-Charlie:DNA	9.761486231	0.394264	0.542574	-0.14831	0.403333
L1P4e:L1:LINE	5.036566447	0.540203	0.68814	-0.14794	0.404272
HERVK22-int:ERVK:LTR	7.597326999	0.473828	0.62112	-0.14729	0.405446
MLT1H1-int:ERVL-MaLR:LTR	5.084084273	0.540513	0.687494	-0.14698	0.405869
Tigger16b:TcMar-Tigger:DNA	8.712056097	0.423019	0.570001	-0.14698	0.405915
LTR19-int:ERV1:LTR	7.773112173	0.466465	0.613303	-0.14684	0.40615
MER47A:TcMar-Tigger:DNA	9.377008898	0.402821	0.549443	-0.14662	0.406479
LTR78B:ERV1:LTR	8.974560275	0.41562	0.561153	-0.14553	0.408216
Tigger9b:TcMar-Tigger:DNA	8.501264247	0.431869	0.577364	-0.14549	0.40831
Tigger17c:TcMar-Tigger:DNA	8.082094592	0.451903	0.597391	-0.14549	0.408357
Tigger3a:TcMar-Tigger:DNA	10.80716817	0.3935	0.53879	-0.14529	0.408779
Arthur1B:hAT-Tip100:DNA	8.748839886	0.423932	0.568744	-0.14481	0.409718
UCON55:TcMar-Tigger:DNA	6.082998383	0.531784	0.676457	-0.14467	0.410094
MLT2B4:ERVL:LTR	9.652785778	0.399606	0.544076	-0.14447	0.410376
MER96B:hAT-Tip100:DNA	8.83798231	0.421898	0.56571	-0.14381	0.411784
AluSg4:Alu:SINE	12.7106459	0.427432	0.570792	-0.14336	0.41277
L1MEc:L1:LINE	12.68179915	0.426743	0.569976	-0.14323	0.412958
HERVL18-int:ERVL:LTR	7.320684989	0.490736	0.633771	-0.14304	0.413427
MER34A1:ERV1:LTR	7.470654122	0.483575	0.626571	-0.143	0.413662
MER102a:hAT-Charlie:DNA	10.40216735	0.395102	0.538038	-0.14294	0.413944
MLT2B2:ERVL:LTR	7.927272829	0.462837	0.605539	-0.1427	0.41446
MSTD-int:ERVL-MaLR:LTR	9.011864494	0.417327	0.559941	-0.14261	0.414742
L1PA10:L1:LINE	11.8539396	0.408589	0.550932	-0.14234	0.415352

Tigger6a:TcMar-Tigger:DNA	7.857458586	0.466833	0.609127	-0.14229	0.415493
LTR39:ERV1:LTR	8.937096869	0.420262	0.562386	-0.14212	0.415869
L1PB3:L1:LINE	10.20404918	0.396866	0.538671	-0.14181	0.416385
L1M2a:L1:LINE	9.151308858	0.415244	0.555597	-0.14035	0.419437
LTR22C0:ERVK:LTR	4.826913649	0.551112	0.690702	-0.13959	0.42108
MLT1D:ERVL-MaLR:LTR	12.87799076	0.436344	0.575728	-0.13938	0.421408
L1MD3:L1:LINE	11.24934086	0.402986	0.542307	-0.13932	0.421502
Charlie10:hAT-Charlie:DNA	7.73293187	0.475947	0.61521	-0.13926	0.421643
SVA_D:SVA:Retroposon	14.98218982	0.528961	0.667902	-0.13894	0.422113
PABL_A-int:ERV1:LTR	7.889266805	0.468923	0.607504	-0.13858	0.42277
MER4D1:ERV1:LTR	9.444376731	0.40982	0.547883	-0.13806	0.423803
MLT1F:ERVL-MaLR:LTR	10.68828541	0.400283	0.538291	-0.13801	0.423897
ORSL:hAT-Tip100:DNA	8.092365316	0.458947	0.596849	-0.1379	0.424225
L1ME2z:L1:LINE	11.09773601	0.403096	0.540836	-0.13774	0.42446
LTR60:ERV1:LTR	3.813275152	0.55499	0.692017	-0.13703	0.425587
L1ME3D:L1:LINE	10.26491206	0.401941	0.538405	-0.13646	0.426854
SVA_E:SVA:Retroposon	12.61074672	0.431878	0.56801	-0.13613	0.427465
AluYk3:Alu:SINE	9.947327193	0.40471	0.540508	-0.1358	0.427934
HAL1:L1:LINE	13.42572743	0.458739	0.594328	-0.13559	0.428263
MER91A:hAT-Tip100:DNA	10.42630995	0.403287	0.538007	-0.13472	0.429624
MLT1A0:ERVL-MaLR:LTR	12.20146572	0.42319	0.557901	-0.13471	0.429718
MamTip3:hAT-Tip100:DNA	7.907195809	0.472041	0.606581	-0.13454	0.430235
L1M3f:L1:LINE	8.762496482	0.433894	0.568278	-0.13438	0.430516
L1P1:L1:LINE	10.29760034	0.404226	0.538288	-0.13406	0.43108
LTR103b_Mam:ERV1:LTR	7.217414741	0.505417	0.639	-0.13358	0.431737
LTR10B2:ERV1:LTR	4.338895234	0.560634	0.694162	-0.13353	0.432066
LTR22A:ERVK:LTR	6.218161965	0.541779	0.675022	-0.13324	0.432394
LTR16A:ERVL:LTR	9.752056624	0.410414	0.542696	-0.13228	0.434085
HERVL74-int:ERVL:LTR	7.277282568	0.503831	0.635952	-0.13212	0.434272
MER74B:ERVL:LTR	8.795549329	0.4356	0.567151	-0.13155	0.434977
MLT1C:ERVL-MaLR:LTR	13.16060259	0.453347	0.584856	-0.13151	0.43507
LTR2:ERV1:LTR	9.146175974	0.425139	0.555751	-0.13061	0.436948
Tigger2:TcMar-Tigger:DNA	10.99468891	0.409415	0.539994	-0.13058	0.436995
Tigger13a:TcMar-Tigger:DNA	10.03250212	0.4096	0.53977	-0.13017	0.4377
L1MA5:L1:LINE	10.70129675	0.408483	0.538334	-0.12985	0.438685
L1MA10:L1:LINE	9.533141157	0.416386	0.546063	-0.12968	0.439061
HERV9NC-int:ERV1:LTR	7.593627256	0.492358	0.621268	-0.12891	0.44
LTR7:ERV1:LTR	10.11471892	0.41049	0.539179	-0.12869	0.440423
LTR31:ERV1:LTR	5.831267818	0.54975	0.678333	-0.12858	0.440798
L1MB1:L1:LINE	10.66206619	0.4099	0.538211	-0.12831	0.441174
Charlie22a:hAT-Charlie:DNA	8.354655791	0.456034	0.583659	-0.12763	0.442207
L1ME3F:L1:LINE	10.54018414	0.411131	0.53799	-0.12686	0.443568
MER90:ERV1:LTR	7.650459882	0.492126	0.618913	-0.12679	0.443662
MSTB1:ERVL-MaLR:LTR	10.16410456	0.412413	0.538881	-0.12647	0.444272
MER76-int:ERVL:LTR	5.045041568	0.562822	0.688026	-0.1252	0.446808
MLT1B-int:ERVL-MaLR:LTR	8.21017939	0.465889	0.590727	-0.12484	0.4477
MamRep1879:hAT-Tip100:DNA	8.5357641	0.45147	0.576054	-0.12458	0.448357

MER113B:hAT-Charlie:DNA	7.257317773	0.512663	0.636964	-0.1243	0.44892
LTR33A_:ERVL:LTR	9.072153546	0.433882	0.558023	-0.12414	0.449343
Tigger4a:TcMar-Tigger:DNA	10.44061454	0.414754	0.537993	-0.12324	0.450423
Charlie24:hAT-Charlie:DNA	8.896682365	0.441161	0.563732	-0.12257	0.451174
MER101-int:ERV1:LTR	8.21971028	0.467846	0.590242	-0.1224	0.451596
L1ME3Cz:L1:LINE	11.88426541	0.431175	0.551481	-0.12031	0.455352
L1M3e:L1:LINE	7.12627435	0.523451	0.643674	-0.12022	0.455775
L1MEi:L1:LINE	10.66899436	0.418347	0.538231	-0.11988	0.456385
MER21C:ERVL:LTR	11.47830762	0.425609	0.545053	-0.11944	0.457465
FLAM_C:Alu:SINE	13.44120602	0.475493	0.594908	-0.11941	0.457512
HERVH48-int:ERV1:LTR	7.966287487	0.484575	0.6035	-0.11892	0.458732
MER21A:ERVL:LTR	10.47150259	0.419305	0.537974	-0.11867	0.459296
ACRO1:acro:Satellite	3.416830345	0.567477	0.685764	-0.11829	0.460282
L1P3:L1:LINE	10.19067591	0.421223	0.538738	-0.11752	0.461596
HUERS-P2-int:ERV1:LTR	5.89191491	0.560645	0.677918	-0.11727	0.462019
L1M3c:L1:LINE	6.882547226	0.538737	0.655729	-0.11699	0.462676
LTR6A:ERV1:LTR	8.006392396	0.48479	0.601389	-0.1166	0.463333
AluYf1:Alu:SINE	10.61285014	0.421502	0.538093	-0.11659	0.46338
L1P4a:L1:LINE	6.82571385	0.541806	0.658312	-0.11651	0.463568
LTR19B:ERV1:LTR	7.174212107	0.525415	0.641215	-0.1158	0.464601
Charlie26a:hAT-Charlie:DNA	7.132545116	0.52769	0.643353	-0.11566	0.46493
L2-3_Crp:L2:LINE	9.527716132	0.43211	0.546166	-0.11406	0.467465
L1PA15:L1:LINE	11.63531277	0.434076	0.547302	-0.11323	0.468685
HERVK13-int:ERVK:LTR	6.153782399	0.562594	0.675765	-0.11317	0.468779
MLT1F1:ERVL-MaLR:LTR	9.936254014	0.428116	0.540614	-0.1125	0.469812
LTR69:ERVL:LTR	4.062791478	0.582653	0.693921	-0.11127	0.472019
L1M3:L1:LINE	10.69838455	0.427248	0.538324	-0.11108	0.472347
MER113:hAT-Charlie:DNA	10.02310239	0.429228	0.539845	-0.11062	0.473192
Tigger8:TcMar-Tigger:DNA	8.669485304	0.461039	0.571454	-0.11041	0.473286
ORSL-2b:hAT-Tip100:DNA	7.778476286	0.50295	0.613044	-0.11009	0.473662
MLT2A2:ERVL:LTR	9.4878605	0.437317	0.546956	-0.10964	0.47446
L1MCc:L1:LINE	9.793433555	0.432552	0.542174	-0.10962	0.474554
D20S16:Satellite:Satellite	5.793254426	0.569149	0.678597	-0.10945	0.47493
L1M4a2:L1:LINE	9.516813607	0.437085	0.546377	-0.10929	0.475446
L2-1_AMi:L2:LINE	8.449397637	0.471504	0.579465	-0.10796	0.47784
LTR25-int:ERV1:LTR	6.897304141	0.547326	0.655039	-0.10771	0.478404
L1MEg1:L1:LINE	7.163446339	0.534289	0.641767	-0.10748	0.478779
LTR16A1:ERVL:LTR	9.26549224	0.445113	0.552323	-0.10721	0.479249
L1MEj:L1:LINE	9.550813139	0.438536	0.545736	-0.1072	0.479343
Charlie20a:hAT-Charlie:DNA	7.552249174	0.516424	0.622976	-0.10655	0.480423
MER30:hAT-Charlie:DNA	11.0055542	0.433569	0.540077	-0.10651	0.480469
AluYe6:Alu:SINE	7.671462718	0.511752	0.618	-0.10625	0.480939
LTR45:ERV1:LTR	5.723691208	0.572946	0.679108	-0.10616	0.48108
L1ME4b:L1:LINE	13.08327008	0.476333	0.58226	-0.10593	0.481502
MER44B:TcMar-Tigger:DNA	9.556306257	0.440117	0.545636	-0.10552	0.482394
MER83B:ERV1:LTR	7.647126632	0.515488	0.619056	-0.10357	0.485915
MER1B:hAT-Charlie:DNA	11.79125041	0.446496	0.549832	-0.10334	0.486291

CR1-3_Croc:CR1:LINE	8.151750684	0.490634	0.593738	-0.1031	0.486573
L4_C_Mam:RTE-X:LINE	10.39806837	0.43505	0.538044	-0.10299	0.486761
THE1A-int:ERVL-MaLR:LTR	10.25667721	0.435545	0.538437	-0.10289	0.486995
Eulor8:TcMar:DNA	4.571021343	0.591544	0.69303	-0.10149	0.489296
AluYh3:Alu:SINE	10.55420234	0.436992	0.538003	-0.10101	0.490141
MER67C:ERV1:LTR	8.425191464	0.479619	0.580495	-0.10088	0.490282
MER77:ERVL:LTR	9.481174871	0.44704	0.547095	-0.10005	0.491596
AluJr4:Alu:SINE	13.66108517	0.503423	0.603476	-0.10005	0.491643
Tigger3c:TcMar-Tigger:DNA	9.2588057	0.452559	0.552506	-0.09995	0.491972
HAL1M8:L1:LINE	9.579877045	0.445448	0.545223	-0.09978	0.4923
HERV17-int:ERV1:LTR	8.488153679	0.479402	0.57788	-0.09848	0.494178
MER92B:ERV1:LTR	8.684465228	0.472613	0.570943	-0.09833	0.494413
L1M3b:L1:LINE	7.473171268	0.5293	0.626456	-0.09716	0.496338
MER68B:ERVL:LTR	8.87913257	0.467958	0.564321	-0.09636	0.497371
MER20B:hAT-Charlie:DNA	10.53825501	0.442264	0.537989	-0.09572	0.498357
L1M3d:L1:LINE	6.670082276	0.568957	0.664637	-0.09568	0.498404
L1MA1:L1:LINE	10.5250526	0.44233	0.53798	-0.09565	0.498498
MSTB2:ERVL-MaLR:LTR	9.176132366	0.459635	0.55486	-0.09523	0.499437
MER44C:TcMar-Tigger:DNA	8.670467858	0.477456	0.57142	-0.09396	0.501737
MER96:hAT-Tip100:DNA	8.365665407	0.489236	0.583151	-0.09391	0.501784
L1P4:L1:LINE	9.082037528	0.464508	0.557714	-0.09321	0.503052
LTR46-int:ERV1:LTR	5.877383993	0.584866	0.678017	-0.09315	0.503239
LTR37A:ERV1:LTR	8.511744172	0.484472	0.576958	-0.09249	0.504272
Charlie15b:hAT-Charlie:DNA	9.793374225	0.452564	0.542175	-0.08961	0.508592
AluSz6:Alu:SINE	15.20909835	0.591724	0.681228	-0.0895	0.508826
L4_A_Mam:RTE-X:LINE	10.77463974	0.449372	0.538631	-0.08926	0.509155
MER61-int:ERV1:LTR	8.196206184	0.502523	0.591441	-0.08892	0.51
OldhAT1:hAT-Ac:DNA	9.994528521	0.45157	0.540083	-0.08851	0.510751
AluSq4:Alu:SINE	10.74799059	0.450224	0.538513	-0.08829	0.511127
LTR36:ERV1:LTR	7.67707756	0.530531	0.617752	-0.08722	0.512817
L1MEh:L1:LINE	9.627691375	0.457243	0.544453	-0.08721	0.512864
MER51A:ERV1:LTR	8.265724671	0.500978	0.587933	-0.08696	0.513239
HUERS-P3-int:ERV1:LTR	8.268920101	0.501128	0.587775	-0.08665	0.513662
UCON35:UCON35:Unknown	4.352842458	0.607802	0.694124	-0.08632	0.514131
MER9a1:ERVK:LTR	7.663442497	0.533578	0.618351	-0.08477	0.516338
L1MD:L1:LINE	11.25643764	0.457833	0.542383	-0.08455	0.516714
HERV35I-int:ERV1:LTR	7.194972304	0.555907	0.64015	-0.08424	0.517324
MER21B:ERVL:LTR	11.09659712	0.458127	0.540826	-0.0827	0.519718
MER1A:hAT-Charlie:DNA	11.5854656	0.464975	0.546556	-0.08158	0.521408
L1M1:L1:LINE	12.05101451	0.473941	0.554703	-0.08076	0.522723
Charlie7:hAT-Charlie:DNA	9.951186043	0.459917	0.540472	-0.08055	0.523239
L1M2a1:L1:LINE	7.899737861	0.526634	0.606966	-0.08033	0.523615
MER48:ERV1:LTR	7.835270651	0.531077	0.610246	-0.07917	0.525117
LTR5_Hs:ERVK:LTR	9.951966798	0.461492	0.540465	-0.07897	0.525587
L1PB2:L1:LINE	9.787690847	0.464235	0.542245	-0.07801	0.526761
LTR80A:ERVL:LTR	5.200165108	0.608045	0.685837	-0.07779	0.527136
MER51C:ERV1:LTR	7.503385686	0.549117	0.625095	-0.07598	0.529202

AluYe5:Alu:SINE	10.10042056	0.463364	0.539273	-0.07591	0.529343
LTR9:ERV1:LTR	8.222213949	0.515004	0.590115	-0.07511	0.53061
LTR1C1:ERV1:LTR	5.527512793	0.606227	0.680982	-0.07475	0.531174
X7A_LINE:CR1:LINE	8.771736451	0.496947	0.567963	-0.07102	0.536714
L1MEb:L1:LINE	8.693000954	0.500167	0.570652	-0.07048	0.537324
MER63D:hAT-Blackjack:DNA	8.498377688	0.507091	0.577476	-0.07039	0.537512
Tigger16a:TcMar-Tigger:DNA	8.963850845	0.491152	0.561504	-0.07035	0.537559
FRAM:Alu:SINE	11.86495737	0.480942	0.55113	-0.07019	0.537793
LTR1D:ERV1:LTR	7.411449371	0.559682	0.629337	-0.06966	0.538404
MamRep605:LTR:LTR?	10.13867868	0.470197	0.539029	-0.06883	0.539202
MamGyp-int:Gypsy:LTR	10.02521753	0.471496	0.539828	-0.06833	0.539812
MLT1A-int:ERVL-MaLR:LTR	6.914107062	0.585992	0.654246	-0.06825	0.539953
L1MA4A:L1:LINE	11.87923998	0.483394	0.551389	-0.068	0.540188
L1MB5:L1:LINE	12.27171956	0.492796	0.55949	-0.06669	0.542394
THE1D-int:ERVL-MaLR:LTR	9.626822206	0.478512	0.544467	-0.06596	0.543756
LTR10B1:ERV1:LTR	6.416916299	0.606218	0.671783	-0.06557	0.544366
MER41-int:ERV1:LTR	8.631968669	0.507185	0.572731	-0.06555	0.544413
MamRep137:TcMar-Tigger:DNA	9.665931562	0.478428	0.543882	-0.06545	0.544742
LTR37-int:ERV1:LTR	7.995368608	0.537183	0.60197	-0.06479	0.545869
AluYa5:Alu:SINE	11.69678143	0.484496	0.548264	-0.06377	0.547512
THE1C-int:ERVL-MaLR:LTR	9.610805629	0.480997	0.544715	-0.06372	0.547606
L1P2:L1:LINE	9.999594793	0.478918	0.54004	-0.06112	0.550939
HERV9-int:ERV1:LTR	7.612404396	0.559687	0.620509	-0.06082	0.551596
MamRep434:TcMar-Tigger:DNA	9.387201203	0.489263	0.549198	-0.05993	0.553099
L1M8:L1:LINE	9.002207461	0.500362	0.560253	-0.05989	0.553286
AluSq10:Alu:SINE	10.37797246	0.481745	0.538079	-0.05633	0.557606
LTR86B2:ERVL:LTR	5.826646936	0.622184	0.678364	-0.05618	0.557887
L1MEd:L1:LINE	12.78014365	0.517302	0.5728	-0.0555	0.559296
MER61B:ERV1:LTR	7.224133787	0.584352	0.638656	-0.0543	0.560892
(GAATG)n:Satellite:Satellite	5.226833184	0.63124	0.685442	-0.0542	0.561127
AluSx4:Alu:SINE	13.35872642	0.537698	0.591851	-0.05415	0.561268
MER4C:ERV1:LTR	7.477311697	0.572186	0.626267	-0.05408	0.561549
LTR22E:ERVK:LTR	6.839508435	0.604014	0.657696	-0.05368	0.562207
AluYh3a3:Alu:SINE	8.383058749	0.529729	0.582358	-0.05263	0.563615
L1M4a1:L1:LINE	10.05535852	0.491065	0.539593	-0.04853	0.569437
Tigger10:TcMar-Tigger:DNA	8.930695166	0.516099	0.562598	-0.0465	0.571502
LTR30:ERV1:LTR	5.476829984	0.635298	0.681629	-0.04633	0.571737
HERVK11-int:ERVK:LTR	6.719537946	0.616438	0.662763	-0.04633	0.571784
L1MDb:L1:LINE	8.216987582	0.544306	0.59038	-0.04607	0.572207
L1MA6:L1:LINE	10.61942977	0.492367	0.538106	-0.04574	0.57277
HERV15-int:ERV1:LTR	7.826617825	0.565739	0.610678	-0.04494	0.573944
HERVE-int:ERV1:LTR	8.742281888	0.524508	0.568968	-0.04446	0.574789
L1M6B:L1:LINE	7.451150033	0.583523	0.627469	-0.04395	0.575634
L1PA15-16:L1:LINE	8.972246415	0.518007	0.561229	-0.04322	0.57662
LTR19C:ERV1:LTR	5.951854305	0.634534	0.677499	-0.04297	0.577136
MSTC:ERVL-MaLR:LTR	11.00197601	0.498171	0.540049	-0.04188	0.578873
MLT1H-int:ERVL-MaLR:LTR	6.173357354	0.634129	0.675552	-0.04142	0.579484

Charlie25:hAT-Charlie:DNA	7.690204153	0.576013	0.617167	-0.04115	0.579812
LTR7B:ERV1:LTR	9.554008071	0.505143	0.545678	-0.04053	0.580798
MLT1I-int:ERVL-MaLR:LTR	8.07227887	0.559268	0.597909	-0.03864	0.58385
AluSc5:Alu:SINE	12.31477988	0.5222	0.560494	-0.03829	0.58446
MLT2F:ERVL:LTR	8.643103997	0.534399	0.572352	-0.03795	0.584836
L1MA3:L1:LINE	12.00146971	0.517301	0.55371	-0.03641	0.586573
MER63B:hAT-Blackjack:DNA	9.169488813	0.519421	0.555056	-0.03564	0.588075
MLT1H1:ERVL-MaLR:LTR	10.15663187	0.503423	0.538923	-0.0355	0.58831
L1MA2:L1:LINE	11.53182237	0.510773	0.545786	-0.03501	0.588967
MER11C:ERVK:LTR	10.50918725	0.504153	0.537973	-0.03382	0.590188
HERVIP10B3-int:ERV1:LTR	8.834465327	0.532632	0.565829	-0.0332	0.591408
L1PA2:L1:LINE	12.43793436	0.530408	0.56349	-0.03308	0.591643
HERVE_a-int:ERV1:LTR	7.084494533	0.614054	0.64581	-0.03176	0.593803
HERVL-int:ERVL:LTR	9.713383959	0.51406	0.543212	-0.02915	0.597042
L1MD1:L1:LINE	11.42081197	0.51608	0.544304	-0.02822	0.598028
FAM:Alu:SINE	11.19221027	0.51377	0.54172	-0.02795	0.598638
DNA1_Mam:TcMar:DNA	6.51387963	0.643155	0.669527	-0.02637	0.600657
AluYi6:Alu:SINE	8.718485954	0.54386	0.569781	-0.02592	0.601127
L1HS:L1:LINE	10.97798938	0.515754	0.539869	-0.02412	0.60385
L1MCb:L1:LINE	9.201227687	0.532688	0.554129	-0.02144	0.606995
L1MA5A:L1:LINE	10.31593098	0.52042	0.538231	-0.01781	0.611925
HERVK-int:ERVK:LTR	8.330273444	0.5712	0.584803	-0.0136	0.617089
L1ME3C:L1:LINE	10.30194604	0.525597	0.538274	-0.01268	0.618263
MER41E:ERV1:LTR	7.505561732	0.61239	0.624999	-0.01261	0.618357
HERVH-int:ERV1:LTR	11.3732934	0.53436	0.543715	-0.00936	0.62216
MER70-int:ERVL:LTR	6.827300369	0.64982	0.658241	-0.00842	0.623521
MER63A:hAT-Blackjack:DNA	9.627361024	0.53669	0.544458	-0.00777	0.624131
PABL_A:ERV1:LTR	8.12580775	0.587957	0.595093	-0.00714	0.624883
LTR46:ERV1:LTR	7.08256931	0.645182	0.645908	-0.00073	0.632394
L1MEa:L1:LINE	7.216339378	0.638534	0.639055	-0.00052	0.632676
X6B_LINE:CR1:LINE	7.502427806	0.62891	0.625138	0.003772	0.637606
MER97b:hAT-Tip100:DNA	5.754436449	0.684595	0.678876	0.005719	0.639531
L1M2:L1:LINE	11.5798609	0.552486	0.546473	0.006012	0.639906
AluYc3:Alu:SINE	9.346659968	0.559538	0.550192	0.009346	0.643897
L1PBa1:L1:LINE	8.309627484	0.596821	0.585789	0.011032	0.646103
MER66B:ERV1:LTR	8.902238852	0.576873	0.563546	0.013327	0.649718
MER5C1:hAT-Charlie:DNA	7.776923614	0.631273	0.613119	0.018154	0.655117
MER4D0:ERV1:LTR	7.698752374	0.636982	0.616782	0.0202	0.657183
LTR6B:ERV1:LTR	8.124814737	0.616594	0.595145	0.021449	0.659202
Looper:PiggyBac:DNA	8.990277801	0.585028	0.56064	0.024388	0.66277
MER51D:ERV1:LTR	8.100412688	0.622986	0.596426	0.02656	0.664883
MER41A:ERV1:LTR	9.962516989	0.568843	0.540367	0.028476	0.666714
Kanga11a:TcMar-Tc2:DNA	7.807805203	0.653009	0.611611	0.041398	0.680047
LTR1A2:ERV1:LTR	9.593248304	0.588285	0.544999	0.043286	0.681831
HERV1_I-int:ERV1:LTR	5.261366042	0.745043	0.684924	0.060119	0.696948
SVA_F:SVA:Retroposon	14.61997382	0.709004	0.648012	0.060992	0.69784
AluYk2:Alu:SINE	9.31049756	0.61845	0.551119	0.067332	0.702958

LTR40A1:ERVL:LTR	7.685407845	0.686493	0.617382	0.069112	0.704319
LTR28:ERV1:LTR	5.161803426	0.757765	0.686397	0.071369	0.707136
Charlie29a:hAT-Charlie:DNA	8.89841117	0.635844	0.563674	0.072169	0.707746
L1M2b:L1:LINE	7.623215467	0.693853	0.620063	0.07379	0.709577
LTR32:ERVL:LTR	8.251397964	0.664679	0.588646	0.076033	0.711878
MER61F:ERV1:LTR	6.909149534	0.732336	0.654481	0.077855	0.713052
L1PBa:L1:LINE	10.13241051	0.62582	0.539067	0.086753	0.721596
HERV-Fc1-int:ERV1:LTR	4.07791062	0.787954	0.693985	0.093969	0.72784
MSR1:Satellite:Satellite	10.85494069	0.642527	0.539055	0.103472	0.736667
MER34B-int:ERV1:LTR	9.160510804	0.66135	0.555322	0.106027	0.738216
HSAT4:centr:Satellite	4.859070924	0.803667	0.690343	0.113324	0.744085
MLT1G-int:ERVL-MaLR:LTR	5.154082135	0.805946	0.686508	0.119439	0.749624
MER67B:ERV1:LTR	9.115694234	0.67745	0.556674	0.120775	0.750798
L1M:L1:LINE	7.854199891	0.737567	0.609292	0.128275	0.757559
SAR:Satellite:Satellite	4.33044964	0.832104	0.694182	0.137922	0.763521
HERVIP10FH-int:ERV1:LTR	8.888625499	0.708177	0.564002	0.144175	0.768732
HERVK9-int:ERVK:LTR	9.54561758	0.691152	0.545831	0.145321	0.769671
LTR101_Mam:ERVL:LTR	8.836451088	0.713009	0.565761	0.147247	0.770986
MER50C:ERV1:LTR	6.293113032	0.822017	0.673988	0.148029	0.771408
HERV1_LTRd:ERV1:LTR	6.604903619	0.821451	0.666881	0.154571	0.776432
LTR7Y:ERV1:LTR	7.53637796	0.789196	0.623653	0.165543	0.784319
Harlequin-int:ERV1:LTR	9.40481877	0.716952	0.548781	0.168171	0.786385
MST-int:ERVL-MaLR:LTR	13.39586952	0.774594	0.593217	0.181377	0.795915
GSATII:centr:Satellite	8.212847435	0.794936	0.590591	0.204345	0.811174
REP522:telo:Satellite	5.84780325	0.889768	0.678219	0.211548	0.815587
HERV3-int:ERV1:LTR	9.096928745	0.769518	0.557252	0.212266	0.816197
HERVS71-int:ERV1:LTR	8.8164163	0.780198	0.566441	0.213757	0.817183
SST1:centr:Satellite	8.602673027	0.794646	0.573724	0.220922	0.821127
LTR38:ERV1:LTR	5.408318932	0.912843	0.682649	0.230195	0.827183
HERVFH21-int:ERV1:LTR	7.409914372	0.974737	0.62941	0.345328	0.879624
FordPrefect:hAT-Tip100:DNA	10.62687542	0.903813	0.538122	0.36569	0.887042
L1PA12:L1:LINE	9.855755028	0.908588	0.541448	0.36714	0.887606
LSAU:Satellite:Satellite	6.433459483	1.069104	0.671434	0.39767	0.89784
L1P4c:L1:LINE	6.804865148	1.104277	0.659227	0.44505	0.911784
MER57F:ERV1:LTR	8.87664962	1.041744	0.564404	0.47734	0.919484
ALR/Alpha:centr:Satellite	8.486103298	1.11396	0.577962	0.535998	0.932113
HSAT5:Satellite:Satellite	6.56275808	1.438794	0.66818	0.770614	0.961878

Supplemental Table 4B. Pancreatic cancer. Median expression, median absolute deviation (MAD), and expression variation estimation TEs. Ranks are calculated across all genes and transposons.

Transposable element	Median Expression	Obs.MAD	Exp.MAD	EV	EV.pvalue
MER2:TcMar-Tigger:DNA	8.82	0.053689	0.307451	-0.25376	0.000697
L1MEd:L1:LINE	9.02	0.053823	0.305059	-0.25124	0.000896
MLT1A0:ERVL-MaLR:LTR	8.88	0.068086	0.306781	-0.23869	0.00254
MER33:hAT-Charlie:DNA	8.38	0.077878	0.310729	-0.23285	0.003984
L1MEf:L1:LINE	8.49	0.078414	0.310252	-0.23184	0.004134
L1MB4:L1:LINE	9.78	0.054474	0.285048	-0.23057	0.004283
HERVL-int:ERVL:LTR	6.39	0.023271	0.251655	-0.22838	0.004781
FAM:Alu:SINE	7.96	0.083165	0.308736	-0.22557	0.005727
MamGyp-int:Gypsy:LTR	10.14	0.052058	0.275347	-0.22329	0.006723
MLT1G:ERVL-MaLR:LTR	6.96	0.058158	0.27933	-0.22117	0.007371
L1MDa:L1:LINE	7.44	0.080585	0.296862	-0.21628	0.009712
MLT1L:ERVL-MaLR:LTR	7.57	0.084841	0.30067	-0.21583	0.009861
AluSq4:Alu:SINE	6.51	0.04291	0.258434	-0.21552	0.01001
L1MA3:L1:LINE	8.25	0.098068	0.310829	-0.21276	0.012102
L1PB4:L1:LINE	8.28	0.098126	0.310858	-0.21273	0.012152
L4_A_Mam:RTE-X:LINE	7.88	0.095806	0.307581	-0.21178	0.01275
L1PA16:L1:LINE	8.86	0.095828	0.307053	-0.21122	0.012999
L1MEg:L1:LINE	8.31	0.100073	0.310855	-0.21078	0.013198
LTR6A:ERV1:LTR	7.15	0.080024	0.28694	-0.20692	0.016236
HERVH-int:ERV1:LTR	7.73	0.098347	0.304664	-0.20632	0.016734
MER104:TcMar-Tc2:DNA	7.30	0.088423	0.292502	-0.20408	0.018726
FRAM:Alu:SINE	8.00	0.105273	0.309338	-0.20407	0.018776
THE1C:ERVL-MaLR:LTR	7.25	0.087108	0.290668	-0.20356	0.019124
MLT1K:ERVL-MaLR:LTR	8.99	0.103013	0.305489	-0.20248	0.019872
MER21A:ERVL:LTR	7.47	0.095354	0.297802	-0.20245	0.019921
SVA_F:SVA:Retroposon	8.26	0.108812	0.310847	-0.20204	0.02037
AluYc:Alu:SINE	7.22	0.090277	0.289484	-0.19921	0.023059
AluSg4:Alu:SINE	7.76	0.10725	0.30514	-0.19789	0.024553
SVA_D:SVA:Retroposon	7.90	0.110538	0.307972	-0.19743	0.025051
MLT1D:ERVL-MaLR:LTR	9.34	0.101739	0.297786	-0.19605	0.026396
AluSc:Alu:SINE	10.59	0.065767	0.26172	-0.19595	0.026545
MER91B:hAT-Tip100:DNA	6.66	0.070434	0.265791	-0.19536	0.027143
L1M4:L1:LINE	9.48	0.099772	0.293864	-0.19409	0.028986
AluSx3:Alu:SINE	10.46	0.073985	0.265346	-0.19136	0.032571
MSTB:ERVL-MaLR:LTR	7.54	0.109662	0.299942	-0.19028	0.034414
LTR16C:ERVL:LTR	6.78	0.081059	0.27133	-0.19027	0.034464
MLT1C:ERVL-MaLR:LTR	9.55	0.101769	0.291682	-0.18991	0.035062
MER20B:hAT-Charlie:DNA	6.93	0.088658	0.27785	-0.18919	0.035908

L1MCa:L1:LINE	7.36	0.105734	0.294346	-0.18861	0.036954
L1P4:L1:LINE	6.55	0.071846	0.26008	-0.18823	0.037502
MLT1F:ERVL-MaLR:LTR	6.84	0.086121	0.273938	-0.18782	0.0381
L1PA4:L1:LINE	9.53	0.104635	0.292104	-0.18747	0.038797
MER8:TcMar-Tigger:DNA	6.29	0.061878	0.24662	-0.18474	0.042532
BLACKJACK:hAT-Blackjack:DNA	7.20	0.104069	0.288764	-0.1847	0.042582
MLT1B:ERVL-MaLR:LTR	9.47	0.109713	0.293932	-0.18422	0.043428
Plat_L3:CR1:LINE	7.30	0.108138	0.292264	-0.18413	0.043677
L1MC4a:L1:LINE	9.02	0.121541	0.305078	-0.18354	0.045072
MLT1A:ERVL-MaLR:LTR	8.54	0.126539	0.309979	-0.18344	0.045221
MER112:hAT-Charlie:DNA	8.16	0.127257	0.310543	-0.18329	0.045421
Tigger1a_Art:TcMar-Tigger:DNA	6.70	0.084576	0.26779	-0.18321	0.04557
MER91A:hAT-Tip100:DNA	7.95	0.12585	0.308624	-0.18277	0.046018
L1MB3:L1:LINE	10.01	0.096873	0.279398	-0.18253	0.046267
SVA_B:SVA:Retroposon	6.64	0.083766	0.264645	-0.18088	0.048359
MER102a:hAT-Charlie:DNA	7.24	0.111131	0.2904	-0.17927	0.051596
MLT2B4:ERVL:LTR	7.70	0.125074	0.303989	-0.17892	0.052293
L1ME4c:L1:LINE	9.40	0.11779	0.296304	-0.17851	0.05304
L1ME2z:L1:LINE	8.12	0.132969	0.310353	-0.17738	0.054684
MLT1F2:ERVL-MaLR:LTR	7.04	0.105972	0.282789	-0.17682	0.05573
AluSc8:Alu:SINE	9.69	0.110823	0.28748	-0.17666	0.055929
Alu:Alu:SINE	6.63	0.088499	0.264127	-0.17563	0.057772
Charlie9:hAT-Charlie:DNA	6.09	0.059089	0.234412	-0.17532	0.058419
L1MCc:L1:LINE	6.54	0.085639	0.25986	-0.17422	0.060461
L1ME4a:L1:LINE	11.58	0.068502	0.242304	-0.1738	0.061208
L1MC:L1:LINE	7.83	0.13305	0.306648	-0.1736	0.061407
Tigger13a:TcMar-Tigger:DNA	7.45	0.123532	0.29711	-0.17358	0.061507
Charlie1a:hAT-Charlie:DNA	8.43	0.137197	0.310559	-0.17336	0.062005
MER58B:hAT-Charlie:DNA	8.18	0.137695	0.310645	-0.17295	0.062902
HAL1ME:L1:LINE	8.78	0.135456	0.307816	-0.17236	0.063649
MER46C:TcMar-Tigger:DNA	7.00	0.108734	0.280897	-0.17216	0.064047
MLT1I:ERVL-MaLR:LTR	7.34	0.121805	0.293675	-0.17187	0.064595
Tigger16a:TcMar-Tigger:DNA	6.24	0.072065	0.24344	-0.17138	0.06594
MER30:hAT-Charlie:DNA	8.03	0.138933	0.309638	-0.1707	0.067334
AluSz6:Alu:SINE	11.34	0.075696	0.245596	-0.1699	0.069127
L1MA5:L1:LINE	7.51	0.129186	0.299015	-0.16983	0.069227
L1MB1:L1:LINE	7.87	0.13773	0.307375	-0.16964	0.069774
MER41B:ERV1:LTR	5.85	0.050807	0.21986	-0.16905	0.07097
Tigger3b:TcMar-Tigger:DNA	8.30	0.141822	0.310855	-0.16903	0.071069
MLT1F1:ERVL-MaLR:LTR	6.43	0.085539	0.25415	-0.16861	0.071767
AluYm1:Alu:SINE	6.96	0.110921	0.279283	-0.16836	0.072265
THE1B:ERVL-MaLR:LTR	8.68	0.141416	0.308874	-0.16746	0.074605
L4_C_Mam:RTE-X:LINE	7.77	0.138913	0.30537	-0.16646	0.076747

Tigger20a:TcMar-Tigger:DNA	6.75	0.103714	0.270161	-0.16645	0.076797
L1PA13:L1:LINE	8.60	0.143544	0.309529	-0.16598	0.077892
Tigger3a:TcMar-Tigger:DNA	7.96	0.143141	0.308832	-0.16569	0.07854
AluSq10:Alu:SINE	5.75	0.047674	0.213261	-0.16559	0.078938
L1M2:L1:LINE	9.07	0.138824	0.304275	-0.16545	0.079337
L1MA10:L1:LINE	7.28	0.126349	0.291714	-0.16536	0.079586
MER81:hAT-Blackjack:DNA	6.45	0.090135	0.255251	-0.16512	0.080034
L1PB2:L1:LINE	6.49	0.092635	0.257304	-0.16467	0.08093
L1PA15:L1:LINE	8.18	0.146133	0.310656	-0.16452	0.081279
L1MC3:L1:LINE	8.65	0.144709	0.30907	-0.16436	0.081777
L1M4a1:L1:LINE	6.18	0.076002	0.240173	-0.16417	0.082225
MER113:hAT-Charlie:DNA	6.93	0.114058	0.277924	-0.16387	0.083221
Tigger17a:TcMar-Tigger:DNA	7.10	0.121351	0.285033	-0.16368	0.08367
AluYh3:Alu:SINE	6.02	0.066771	0.230155	-0.16338	0.084616
L1M3:L1:LINE	7.62	0.138522	0.301905	-0.16338	0.084666
MamTip2:hAT-Tip100:DNA	7.79	0.142782	0.30579	-0.16301	0.08601
L1ME3G:L1:LINE	9.61	0.127013	0.289763	-0.16275	0.086708
CR1_Mam:CR1:LINE	6.28	0.08308	0.245533	-0.16245	0.087753
MLT1H:ERVL-MaLR:LTR	7.97	0.146584	0.308961	-0.16238	0.088102
HAL1b:L1:LINE	7.29	0.129898	0.291973	-0.16207	0.088899
Charlie15a:hAT-Charlie:DNA	6.84	0.113755	0.274032	-0.16028	0.093531
X9_LINE:L1:LINE	8.97	0.146121	0.305769	-0.15965	0.095025
LTR78:ERV1:LTR	6.38	0.092005	0.25133	-0.15932	0.095822
MER44A:TcMar-Tigger:DNA	7.30	0.133716	0.292306	-0.15859	0.097216
OldhAT1:hAT-Ac:DNA	10.16	0.116418	0.274636	-0.15822	0.098411
Penelope1_Vert:Penelope:LINE	6.87524	0.118087	0.275656	-0.15757	0.100702
AluSg7:Alu:SINE	8.323036	0.153796	0.310841	-0.15705	0.102196
MER5A1:hAT-Charlie:DNA	10.47848	0.107719	0.264735	-0.15702	0.102296
MLT1E2:ERVL-MaLR:LTR	6.278543	0.089197	0.245702	-0.15651	0.103591
MER45A:hAT-Tip100:DNA	6.18347	0.084946	0.240252	-0.15531	0.107077
L1MD3:L1:LINE	7.828181	0.151903	0.306614	-0.15471	0.108621
L1MEi:L1:LINE	6.39727	0.097707	0.252243	-0.15454	0.109119
L3b:CR1:LINE	8.880551	0.152374	0.30678	-0.15441	0.109667
MER58C:hAT-Charlie:DNA	6.981828	0.12593	0.280238	-0.15431	0.110115
Charlie4z:hAT-Charlie:DNA	8.566804	0.156077	0.309772	-0.15369	0.111908
Cheshire:hAT-Charlie:DNA	5.399284	0.035561	0.188003	-0.15244	0.115693
HSMAR2:TcMar-Mariner:DNA	6.289886	0.09426	0.24634	-0.15208	0.116639
L1PREC2:L1:LINE	7.831202	0.154739	0.306672	-0.15193	0.117038
L1MC2:L1:LINE	8.311552	0.160184	0.310851	-0.15067	0.120225
AluYk3:Alu:SINE	6.450714	0.10444	0.255092	-0.15065	0.120375
THE1D:ERVL-MaLR:LTR	7.659967	0.15239	0.302936	-0.15055	0.120673
Charlie15b:hAT-Charlie:DNA	5.910702	0.073985	0.223562	-0.14958	0.12401
L1ME3Cz:L1:LINE	8.675667	0.159785	0.308878	-0.14909	0.125305

HERVK9-int:ERVK:LTR	7.258593	0.141869	0.290919	-0.14905	0.125504
Tigger19a:TcMar-Tigger:DNA	8.306604	0.161828	0.310854	-0.14903	0.125654
L1MA4A:L1:LINE	8.589229	0.160703	0.309605	-0.1489	0.126202
L1MC5:L1:LINE	10.28209	0.122954	0.270879	-0.14793	0.130036
MER31A:ERV1:LTR	6.605977	0.115883	0.263032	-0.14715	0.133274
ERVL-E-int:ERVL:LTR	6.663386	0.11873	0.265844	-0.14711	0.133323
AluYj4:Alu:SINE	6.352024	0.102992	0.249785	-0.14679	0.134568
L1PB3:L1:LINE	6.886861	0.129669	0.276168	-0.1465	0.135814
L1M4c:L1:LINE	8.044252	0.163513	0.309756	-0.14624	0.136511
MER53:hAT:DNA	7.244288	0.144677	0.290413	-0.14574	0.138154
L1MA2:L1:LINE	8.183201	0.165126	0.310663	-0.14554	0.138852
L1ME5:L1:LINE	6.533736	0.11407	0.2594	-0.14533	0.139499
MLT1J2:ERVL-MaLR:LTR	7.139639	0.141306	0.286552	-0.14525	0.140047
L1ME4b:L1:LINE	10.25119	0.12664	0.271875	-0.14523	0.140146
MER68:ERVL:LTR	6.571148	0.116212	0.261294	-0.14508	0.140744
HAL1M8:L1:LINE	6.281404	0.101125	0.245863	-0.14474	0.14174
MER117:hAT-Charlie:DNA	7.326445	0.148701	0.293253	-0.14455	0.142238
L1ME3:L1:LINE	8.38776	0.166227	0.310709	-0.14448	0.142537
MER21B:ERVL:LTR	7.029679	0.138111	0.282213	-0.1441	0.144081
MER49:ERV1:LTR	6.429087	0.110788	0.253946	-0.14316	0.14692
MLT1A1:ERVL-MaLR:LTR	7.829353	0.164437	0.306637	-0.1422	0.150356
AluSg:Alu:SINE	10.70403	0.116918	0.25895	-0.14203	0.151352
L1P3:L1:LINE	6.732849	0.127976	0.269161	-0.14118	0.154191
AluSz:Alu:SINE	12.13489	0.097219	0.238235	-0.14102	0.154639
MER20:hAT-Charlie:DNA	9.496198	0.152946	0.293279	-0.14033	0.156532
Tigger2:TcMar-Tigger:DNA	6.800652	0.132033	0.272306	-0.14027	0.156631
L1PA5:L1:LINE	9.116582	0.163737	0.303447	-0.13971	0.158374
MER102b:hAT-Charlie:DNA	7.712449	0.164523	0.304174	-0.13965	0.158574
MamRTE1:RTE-Bo.B:LINE	10.26304	0.132099	0.271493	-0.13939	0.15962
Charlie7:hAT-Charlie:DNA	6.462646	0.116402	0.25572	-0.13932	0.159869
Tigger12c:TcMar-Tigger:DNA	5.560388	0.061403	0.19981	-0.13841	0.163405
L2a:L2:LINE	13.14897	0.101824	0.239816	-0.13799	0.164749
MLT2A1:ERVL:LTR	6.266568	0.107203	0.245026	-0.13782	0.165297
L1P1:L1:LINE	7.115399	0.14782	0.28562	-0.1378	0.165446
Tigger4:TcMar-Tigger:DNA	5.982847	0.09035	0.228129	-0.13778	0.165496
MLT2C1:ERVL:LTR	6.603861	0.125934	0.262927	-0.13699	0.168385
Tigger3c:TcMar-Tigger:DNA	5.714013	0.07376	0.210548	-0.13679	0.169032
MER115:hAT-Tip100:DNA	5.809563	0.080286	0.216973	-0.13669	0.16958
LTR8:ERV1:LTR	6.477274	0.119954	0.256486	-0.13653	0.170128
MER1B:hAT-Charlie:DNA	8.095531	0.173652	0.310184	-0.13653	0.170178
LTR16A:ERVL:LTR	5.915941	0.088006	0.223897	-0.13589	0.172568
MLT1G1:ERVL-MaLR:LTR	5.627091	0.06946	0.204534	-0.13507	0.175507
L1MA8:L1:LINE	8.133193	0.175357	0.310427	-0.13507	0.175606

L1PA8:L1:LINE	7.862068	0.17232	0.307251	-0.13493	0.176005
Helitron3Na_Mam:Helitron:RC	5.887267	0.087131	0.222054	-0.13492	0.176055
X6A_LINE:CR1:LINE	5.765342	0.079367	0.214024	-0.13466	0.17715
LTR16A2:ERVL:LTR	5.616855	0.070279	0.203816	-0.13354	0.180786
L1ME3F:L1:LINE	7.007155	0.147858	0.28129	-0.13343	0.181384
THE1A-int:ERVL-MaLR:LTR	5.623022	0.071348	0.204249	-0.1329	0.183674
MER103C:hAT-Charlie:DNA	8.657839	0.176403	0.309038	-0.13264	0.18482
L1MA1:L1:LINE	6.934833	0.14574	0.278249	-0.13251	0.185318
Charlie5:hAT-Charlie:DNA	5.800159	0.083919	0.216349	-0.13243	0.185567
MLT1H2:ERVL-MaLR:LTR	6.556099	0.128351	0.260536	-0.13219	0.186513
L1ME3C:L1:LINE	6.396132	0.120243	0.252182	-0.13194	0.187908
AluJr:Alu:SINE	12.23546	0.106393	0.237901	-0.13151	0.189601
MER4B-int:ERV1:LTR	5.740805	0.081129	0.212369	-0.13124	0.190747
LTR33A:ERVL:LTR	5.832705	0.087354	0.2185	-0.13115	0.191045
L1ME3E:L1:LINE	7.445815	0.165992	0.297067	-0.13108	0.191494
MIR1_Amn:MIR:SINE	9.620311	0.158748	0.289534	-0.13079	0.19239
MER74B:ERVL:LTR	5.506875	0.065231	0.19595	-0.13072	0.192589
AluSc5:Alu:SINE	8.477873	0.179686	0.310333	-0.13065	0.193087
X6B_LINE:CR1:LINE	6.342548	0.11887	0.249265	-0.13039	0.194332
MER1A:hAT-Charlie:DNA	7.630603	0.17198	0.302208	-0.13023	0.195129
THE1D-int:ERVL-MaLR:LTR	5.611437	0.073825	0.203434	-0.12961	0.19757
MER57A-int:ERV1:LTR	7.107524	0.155861	0.285314	-0.12945	0.198117
L4_B_Mam:RTE-X:LINE	6.867099	0.145943	0.275296	-0.12935	0.198715
L1M1:L1:LINE	8.394325	0.181706	0.310689	-0.12898	0.19991
Tigger5b:TcMar-Tigger:DNA	6.357403	0.121771	0.25008	-0.12831	0.202002
MER65C:ERV1:LTR	5.424548	0.061591	0.189892	-0.1283	0.202102
L1ME3B:L1:LINE	7.921548	0.180231	0.308248	-0.12802	0.203646
L1MC4:L1:LINE	10.54245	0.136115	0.262902	-0.12679	0.208726
L1MD1:L1:LINE	8.03266	0.182932	0.309644	-0.12671	0.209124
AluJb:Alu:SINE	12.69067	0.111183	0.237855	-0.12667	0.209174
L1MD2:L1:LINE	9.04972	0.178008	0.304672	-0.12666	0.209224
AluSq2:Alu:SINE	11.38911	0.118327	0.244802	-0.12647	0.209821
Charlie18a:hAT-Charlie:DNA	6.35697	0.125413	0.250056	-0.12464	0.217242
MER5A:hAT-Charlie:DNA	10.22068	0.148333	0.272857	-0.12452	0.217939
Charlie24:hAT-Charlie:DNA	6.235857	0.119079	0.243279	-0.1242	0.219284
AluYg6:Alu:SINE	6.943019	0.154521	0.278599	-0.12408	0.219732
THE1C-int:ERVL-MaLR:LTR	5.471079	0.069834	0.193334	-0.1235	0.222172
L1ME3A:L1:LINE	9.223731	0.177689	0.301022	-0.12333	0.22282
AluYf1:Alu:SINE	5.693279	0.086048	0.209128	-0.12308	0.224065
MLT1J:ERVL-MaLR:LTR	8.496431	0.187289	0.31023	-0.12294	0.224712
L1MDb:L1:LINE	5.520176	0.074074	0.196915	-0.12284	0.225161
MADE1:TcMar-Mariner:DNA	6.610063	0.140484	0.263234	-0.12275	0.225509
MamGypsy2-LTR:Gypsy:LTR	5.600509	0.080342	0.202663	-0.12232	0.227302

MER94:hAT-Blackjack:DNA	6.682543	0.144663	0.266768	-0.1221	0.22775
L2c:L2:LINE	12.71823	0.11642	0.237921	-0.1215	0.230091
MSTB1:ERVL-MaLR:LTR	6.488008	0.135742	0.257045	-0.1213	0.230838
L1MB8:L1:LINE	9.77551	0.163951	0.285173	-0.12122	0.231336
X7C_LINE:CR1:LINE	6.028822	0.109839	0.230982	-0.12114	0.231486
AluSx:Alu:SINE	12.64177	0.116641	0.237755	-0.12111	0.231735
Charlie4a:hAT-Charlie:DNA	6.883493	0.155426	0.27602	-0.12059	0.234075
MSR1:Satellite:Satellite	9.003715	0.184933	0.305361	-0.12043	0.234872
Tigger4a:TcMar-Tigger:DNA	7.632586	0.183135	0.302258	-0.11912	0.239703
MSTD:ERVL-MaLR:LTR	7.143775	0.167628	0.28671	-0.11908	0.239853
MER51A:ERV1:LTR	5.765699	0.095104	0.214048	-0.11894	0.24045
Charlie1b:hAT-Charlie:DNA	6.678453	0.14812	0.266571	-0.11845	0.242691
L1MB5:L1:LINE	8.951473	0.188541	0.305987	-0.11745	0.246526
MER34A:ERV1:LTR	6.463221	0.138496	0.25575	-0.11725	0.247622
MER50:ERV1:LTR	6.112996	0.119236	0.23609	-0.11685	0.249315
Tigger4b:TcMar-Tigger:DNA	8.405743	0.193967	0.310651	-0.11668	0.250212
MER4-int:ERV1:LTR	6.132013	0.120636	0.237223	-0.11659	0.250411
MER2B:TcMar-Tigger:DNA	6.173079	0.123189	0.239645	-0.11646	0.251009
AmnSINE1:5S-Deu-L2:SINE	6.654909	0.149002	0.265432	-0.11643	0.251058
MamRep605:LTR:LTR?	6.018798	0.114242	0.230364	-0.11612	0.252254
MER44C:TcMar-Tigger:DNA	5.542348	0.082684	0.198515	-0.11583	0.253648
ERV3-16A3_I-int:ERVL:LTR	6.371219	0.135384	0.250833	-0.11545	0.254794
L3:CR1:LINE	12.999	0.123564	0.238971	-0.11541	0.254993
HERVE-int:ERV1:LTR	6.577459	0.146472	0.261611	-0.11514	0.255939
MLT2B1:ERVL:LTR	6.624917	0.150219	0.263967	-0.11375	0.261866
MER6:TcMar-Tigger:DNA	5.598071	0.088831	0.202491	-0.11366	0.262164
L1MEg2:L1:LINE	5.840546	0.105533	0.219014	-0.11348	0.26321
Zaphod:hAT-Tip100:DNA	5.821235	0.104323	0.217744	-0.11342	0.263559
AluJo:Alu:SINE	12.24445	0.125204	0.237877	-0.11267	0.267493
MER63A:hAT-Blackjack:DNA	5.832481	0.106721	0.218485	-0.11176	0.271627
Tigger15a:TcMar-Tigger:DNA	7.747555	0.193281	0.304957	-0.11168	0.271926
MER31B:ERV1:LTR	5.340897	0.072145	0.183586	-0.11144	0.273221
LTR13:ERVK:LTR	5.54957	0.087615	0.199034	-0.11142	0.273271
L1ME2:L1:LINE	9.425386	0.184279	0.295414	-0.11113	0.274217
MER113A:hAT-Charlie:DNA	6.157651	0.127618	0.23874	-0.11112	0.274316
MER119:hAT-Charlie:DNA	5.485074	0.083442	0.19436	-0.11092	0.275014
MER5C:hAT-Charlie:DNA	5.338988	0.073	0.18344	-0.11044	0.277305
MamRep137:TcMar-Tigger:DNA	6.619972	0.153387	0.263723	-0.11034	0.277653
L1M6:L1:LINE	6.564742	0.150727	0.260972	-0.11024	0.278052
MLT1E1A:ERVL-MaLR:LTR	5.917855	0.11449	0.224019	-0.10953	0.280492
MamRep1879:hAT-Tip100:DNA	6.211067	0.13237	0.241854	-0.10948	0.280741
MER102c:hAT-Charlie:DNA	6.941474	0.169496	0.278533	-0.10904	0.28343
LTR33:ERVL:LTR	7.762681	0.196479	0.305283	-0.1088	0.284327

Tigger1:TcMar-Tigger:DNA	9.467588	0.185625	0.294146	-0.10852	0.285373
MER21C:ERVL:LTR	8.958044	0.198335	0.305913	-0.10758	0.289108
L1PB:L1:LINE	5.746376	0.105184	0.212746	-0.10756	0.289307
HAL1:L1:LINE	9.925333	0.174439	0.281562	-0.10712	0.290951
L1MA4:L1:LINE	8.011067	0.202752	0.309418	-0.10667	0.292893
MLT1G3:ERVL-MaLR:LTR	5.626482	0.098128	0.204492	-0.10636	0.293889
L1M8:L1:LINE	5.493061	0.089023	0.194944	-0.10592	0.295582
L1PA12:L1:LINE	7.607655	0.196042	0.301622	-0.10558	0.297077
MER5B:hAT-Charlie:DNA	9.452623	0.189205	0.294597	-0.10539	0.297973
MER3:hAT-Charlie:DNA	8.555761	0.204784	0.309851	-0.10507	0.299118
Charlie13a:hAT-Charlie:DNA	5.707498	0.105555	0.210103	-0.10455	0.30136
Charlie1:hAT-Charlie:DNA	6.015917	0.125997	0.230186	-0.10419	0.302555
Kanga2_a:TcMar-Tc2:DNA	5.350067	0.081607	0.184284	-0.10268	0.30878
MLT2A2:ERVL:LTR	5.711032	0.108237	0.210345	-0.10211	0.310772
L2b:L2:LINE	12.89399	0.136784	0.238497	-0.10171	0.312416
L1PA6:L1:LINE	8.403936	0.209267	0.310658	-0.10139	0.313561
L1PA17:L1:LINE	7.365708	0.193354	0.294549	-0.10119	0.314906
Charlie16a:hAT-Charlie:DNA	6.997826	0.179726	0.280904	-0.10118	0.315105
MSTA-int:ERVL-MaLR:LTR	6.123948	0.136446	0.236744	-0.1003	0.318542
AluSp:Alu:SINE	11.94331	0.138919	0.239209	-0.10029	0.318641
MLT1N2:ERVL-MaLR:LTR	5.580556	0.102239	0.201248	-0.09901	0.323721
Tigger2b_Pri:TcMar-Tigger:DNA	5.842224	0.120428	0.219124	-0.0987	0.325315
L2:L2:LINE	11.38649	0.146152	0.244843	-0.09869	0.325415
MIRb:MIR:SINE	13.44823	0.143456	0.242094	-0.09864	0.325763
MIRc:MIR:SINE	12.7996	0.139642	0.238154	-0.09851	0.326311
hAT-N1_Mam:hAT-Tip100:DNA	5.442144	0.093713	0.191199	-0.09749	0.330893
MER45C:hAT-Tip100:DNA	6.898384	0.179361	0.276672	-0.09731	0.331491
MADE2:TcMar-Mariner:DNA	6.832787	0.176537	0.273764	-0.09723	0.331939
ERVL-B4-int:ERVL:LTR	6.376838	0.155441	0.251139	-0.0957	0.33926
FordPrefect:hAT-Tip100:DNA	6.024885	0.136032	0.230739	-0.09471	0.343045
Mam_R4:Dong-R4:LINE	5.820878	0.123029	0.217721	-0.09469	0.343145
MER6A:TcMar-Tigger:DNA	5.819	0.123103	0.217597	-0.09449	0.34424
HERVH48-int:ERV1:LTR	5.677502	0.114193	0.208042	-0.09385	0.346681
Charlie2a:hAT-Charlie:DNA	5.353871	0.090853	0.184574	-0.09372	0.347577
HSMAR1:TcMar-Mariner:DNA	5.784597	0.122346	0.215313	-0.09297	0.350715
ERV3-16A3_LTR:ERVL:LTR	5.493897	0.102371	0.195005	-0.09263	0.352059
L1MB7:L1:LINE	10.42879	0.173745	0.266233	-0.09249	0.352707
L1MC1:L1:LINE	9.223814	0.208547	0.30102	-0.09247	0.352856
L1ME1:L1:LINE	10.25475	0.179959	0.27176	-0.0918	0.355097
Charlie2b:hAT-Charlie:DNA	5.863549	0.128754	0.220517	-0.09176	0.355247
SVA_E:SVA:Retroposon	6.617184	0.171908	0.263586	-0.09168	0.355546
MLT1J1:ERVL-MaLR:LTR	5.706808	0.118515	0.210056	-0.09154	0.355944
MER41A:ERV1:LTR	6.057305	0.141653	0.232727	-0.09107	0.358036

MER96B:hAT-Tip100:DNA	5.755887	0.122697	0.213388	-0.09069	0.359281
L1PA10:L1:LINE	10.24734	0.18164	0.271999	-0.09036	0.360526
MER61F:ERV1:LTR	5.564219	0.111102	0.200084	-0.08898	0.3671
MLT2D:ERVL:LTR	8.281059	0.222651	0.310857	-0.08821	0.370038
L1MEj:L1:LINE	5.604201	0.115201	0.202924	-0.08772	0.371981
L1MEb:L1:LINE	5.383723	0.099176	0.186833	-0.08766	0.372279
Tigger7:TcMar-Tigger:DNA	6.841524	0.186531	0.274156	-0.08763	0.372479
MSTC:ERVL-MaLR:LTR	6.144516	0.150433	0.237965	-0.08753	0.373027
Tigger2a:TcMar-Tigger:DNA	7.672689	0.216799	0.303243	-0.08644	0.377857
L1PA2:L1:LINE	8.300464	0.224849	0.310857	-0.08601	0.380198
MamRep38:hAT:DNA	6.770466	0.185603	0.270917	-0.08531	0.383186
Tigger19b:TcMar-Tigger:DNA	7.982627	0.224215	0.309088	-0.08487	0.38483
L1P2:L1:LINE	6.226745	0.157897	0.242756	-0.08486	0.38488
MLT1O:ERVL-MaLR:LTR	7.089075	0.20003	0.284592	-0.08456	0.386025
MIR3:MIR:SINE	12.53148	0.15463	0.23761	-0.08298	0.391852
MER63B:hAT-Blackjack:DNA	5.815169	0.134914	0.217344	-0.08243	0.394193
L1PBa:L1:LINE	5.661496	0.125622	0.206934	-0.08131	0.398476
LTR16A1:ERVL:LTR	6.2389	0.162293	0.243453	-0.08116	0.399472
LTR52:ERVL:LTR	5.602422	0.121658	0.202798	-0.08114	0.399671
Ricksha_c:MULE-MuDR:DNA	6.367389	0.169559	0.250625	-0.08107	0.40012
MER105:hAT-Charlie:DNA	6.162438	0.158445	0.239021	-0.08058	0.401863
MER4D1:ERV1:LTR	5.574369	0.120286	0.200808	-0.08052	0.402161
MLT2B3:ERVL:LTR	5.687116	0.128364	0.208705	-0.08034	0.402909
MER65A:ERV1:LTR	5.742162	0.132131	0.212461	-0.08033	0.402958
L1M7:L1:LINE	6.678491	0.186536	0.266573	-0.08004	0.404104
L1MA7:L1:LINE	8.09914	0.230313	0.31021	-0.0799	0.404552
Chap1_Mam:hAT-Charlie:DNA	5.367504	0.106255	0.185608	-0.07935	0.406893
MER4A1_:ERV1:LTR	6.839214	0.194878	0.274053	-0.07917	0.407391
MER106A:hAT-Charlie:DNA	5.673509	0.130182	0.207766	-0.07758	0.41257
MER4E:ERV1:LTR	5.775224	0.137737	0.214686	-0.07695	0.415011
AluYa8:Alu:SINE	6.724938	0.192297	0.268788	-0.07649	0.417102
L1PA11:L1:LINE	7.004761	0.205199	0.281191	-0.07599	0.419045
MLT1H1:ERVL-MaLR:LTR	7.392743	0.219813	0.295418	-0.0756	0.420738
L1M4b:L1:LINE	7.307202	0.217804	0.292603	-0.0748	0.423776
MER58D:hAT-Charlie:DNA	5.958152	0.151879	0.226578	-0.0747	0.424473
Charlie8:hAT-Charlie:DNA	12.15102	0.163954	0.238173	-0.07422	0.426615
MER34C2:ERV1:LTR	7.135491	0.213726	0.286393	-0.07267	0.43289
L1PB1:L1:LINE	9.378193	0.226171	0.296808	-0.07064	0.440759
Charlie19a:hAT-Charlie:DNA	7.949295	0.240931	0.308652	-0.06772	0.450769
Tigger9a:TcMar-Tigger:DNA	5.413371	0.124047	0.189058	-0.06501	0.460083
L1ME3D:L1:LINE	6.98375	0.215882	0.280319	-0.06444	0.462075
LTR49-int:ERV1:LTR	5.566569	0.135881	0.200251	-0.06437	0.462324
MLT1H-int:ERVL-MaLR:LTR	5.669435	0.143386	0.207484	-0.0641	0.463419

L1MA5A:L1:LINE	8.452413	0.246413	0.31046	-0.06405	0.463619
L1PA14:L1:LINE	6.555491	0.197845	0.260505	-0.06266	0.469595
L2-3_Crp:L2:LINE	6.50804	0.197138	0.258082	-0.06094	0.475223
Arthur1B:hAT-Tip100:DNA	7.180919	0.227461	0.288107	-0.06065	0.476518
MER44B:TcMar-Tigger:DNA	7.528943	0.241701	0.299498	-0.0578	0.487126
AluY:Alu:SINE	12.20926	0.181004	0.237976	-0.05697	0.490811
LTR19A:ERV1:LTR	5.526988	0.141021	0.197408	-0.05639	0.492754
L1MD:L1:LINE	13.22535	0.185986	0.240322	-0.05434	0.500025
MER39:ERV1:LTR	5.742087	0.158623	0.212456	-0.05383	0.502216
MIR:MIR:SINE	12.88517	0.184901	0.238462	-0.05356	0.503412
Tigger14a:TcMar-Tigger:DNA	5.476095	0.142049	0.193702	-0.05165	0.50879
LTR37-int:ERV1:LTR	6.532172	0.207718	0.25932	-0.0516	0.50894
L1MEc:L1:LINE	8.968516	0.256838	0.305795	-0.04896	0.517406
L1M5:L1:LINE	12.62799	0.188809	0.237731	-0.04892	0.517506
LTR79:ERVL:LTR	7.531869	0.25189	0.29958	-0.04769	0.520942
MER65-int:ERV1:LTR	5.997019	0.182861	0.229013	-0.04615	0.52657
L1MC5a:L1:LINE	10.82568	0.210643	0.256295	-0.04565	0.528263
MER47A:TcMar-Tigger:DNA	7.014277	0.237166	0.281583	-0.04442	0.532696
THE1A:ERVL-MaLR:LTR	7.127362	0.243489	0.286082	-0.04259	0.538822
7SK:RNA:RNA	9.608393	0.247812	0.289889	-0.04208	0.540366
L1PA8A:L1:LINE	6.683791	0.225746	0.266828	-0.04108	0.543653
ALR/Alpha:centr:Satellite	6.165979	0.200725	0.239229	-0.0385	0.55182
Charlie29a:hAT-Charlie:DNA	5.961813	0.18914	0.226809	-0.03767	0.554958
MER77:ERVL:LTR	5.368117	0.151936	0.185654	-0.03372	0.566811
X7A_LINE:CR1:LINE	7.406655	0.262655	0.295857	-0.0332	0.568206
THE1B-int:ERVL-MaLR:LTR	6.609519	0.233257	0.263207	-0.02995	0.57722
MER57-int:ERV1:LTR	9.59078	0.268571	0.290415	-0.02184	0.599631
L1PA3:L1:LINE	8.706464	0.286808	0.308592	-0.02178	0.599831
L1MA9:L1:LINE	8.986574	0.283962	0.305583	-0.02162	0.600179
MER91C:hAT-Tip100:DNA	5.68096	0.188872	0.20828	-0.01941	0.606704
AluJr4:Alu:SINE	10.1114	0.256911	0.276303	-0.01939	0.606803
LTR12C:ERV1:LTR	9.740931	0.269843	0.286092	-0.01625	0.614423
LTR67B:ERVL:LTR	5.469016	0.176962	0.193182	-0.01622	0.614523
MamRep434:TcMar-Tigger:DNA	5.805033	0.201523	0.216673	-0.01515	0.616963
LTR33B:ERVL:LTR	10.10405	0.264441	0.276529	-0.01209	0.624583
Tigger16b:TcMar-Tigger:DNA	5.546583	0.187505	0.19882	-0.01132	0.626127
MER58A:hAT-Charlie:DNA	9.637567	0.280917	0.289024	-0.00811	0.63549
AluSx4:Alu:SINE	12.49957	0.230628	0.237593	-0.00696	0.637631
AluSq:Alu:SINE	13.54293	0.240749	0.242979	-0.00223	0.649534
Helitron2Na_Mam:Helitron:RC?	5.945754	0.227078	0.225794	0.001283	0.658001
L1MB2:L1:LINE	10.32428	0.28938	0.269522	0.019857	0.700782
MER11B:ERVK:LTR	11.38661	0.265626	0.244841	0.020785	0.702326
UCON80_AMi:UCON80_AMi:Unknown	5.509008	0.219292	0.196105	0.023187	0.706858

MSTA:ERVL-MaLR:LTR	9.993656	0.351487	0.279757	0.071729	0.784551
L1MA6:L1:LINE	7.732143	0.377981	0.304618	0.073363	0.786244
LTR2:ERV1:LTR	8.619393	0.387634	0.309366	0.078268	0.793067
LTR71B:ERV1:LTR	5.688754	0.300036	0.208817	0.091219	0.808755
AluYb8:Alu:SINE	10.9942	0.347149	0.252545	0.094604	0.812491
L1HS:L1:LINE	10.26465	0.366997	0.271441	0.095556	0.813537
AluYe5:Alu:SINE	10.22274	0.379537	0.27279	0.106746	0.825838
Charlie10:hAT-Charlie:DNA	6.425611	0.391926	0.253761	0.138165	0.856118
UCON132b:hAT-Tip100:DNA	8.51027	0.476219	0.310149	0.16607	0.878629
HERV17-int:ERV1:LTR	8.713891	0.496074	0.308521	0.187553	0.890931
MARNA:TcMar-Mariner:DNA	7.858458	0.504686	0.307185	0.197502	0.895961
AluYa5:Alu:SINE	10.73572	0.51601	0.258269	0.257741	0.922805
L1PA7:L1:LINE	10.18965	0.572732	0.273849	0.298883	0.935804
FLAM_A:Alu:SINE	10.66115	0.619879	0.259887	0.359992	0.950396
FLAM_C:Alu:SINE	11.74974	0.600784	0.240655	0.360129	0.950446
AluSx1:Alu:SINE	13.3683	0.655827	0.241408	0.414419	0.961253
AluYb9:Alu:SINE	8.585505	0.838989	0.309633	0.529356	0.975248

Supplemental Table 5

Table of Reagents

1. **Oligos:**

Multiple Myeloma	
**Transposon name**	**Sequence**
AlusSp	5'ACCCGGCCTTGGACACGCCATTTTCAACTCCGTGGTGCGTTTTTTTTTTTTTTTTTTTTTTTTGTAATGGAGTTTTGCTCTTGTTGCCCAGGATGGAGTGCAAGGGATCTTGGCTCACCACAGCCTCTGCCTCCTGGGTTCAAGTGATTCTTCTGCCTCAGCCTCCCAAGTAGCTGGGATTATAAGCACCCACCACCACGCCCAGCTAATTTTGTATTTTTTAGAAGAGATGGAGTTTCTCCAGTTGGCCAGGATGGTCTGTATATCCTGACCTCATGATCTGCCCACCA 3'.
ALUSg2	GGTCAGTCGCGGTGGCTCATACTTGTAATCTCAGCACTTTGGGAGGCCAAGGCGGGTGGATCACCTCAGGTCAGGAGTTCAAGACCAGCCTGACCAATGTGGTAAAACCCTGTCTCTACTAAAAATACAAAAATTAGCTGGGCGTGGTGGCACATGCCTGTAATCCCAGCTACTCAGGAGGCTGAGGCAGGAGAATCGCTTGAACCCGGGAGGCAGAGGTTGCAGTGAGCCAAGATAACACCACTGCACTCCAGCCTGGGTGACAGAGCAAGACTCCGTCTCAAAAAAAAAAAAAAAAAGATCCAGTGATAGG
MER11C	TATCGAAAATTCACCCCCGATATTCACGTAGGTTCTTTTCTATTTTCCCTAAGCGTCGGCAGGTTTGAGAAATAAAGGGACAGAGTACAAAAGAGAGAAATTTTAAAGCTGGGCGTCCGGGGGAGACATCACATGTTGGTAGGTTCCATGATGCCCCACAAGCCGTAAAAACCAGCAAGTTTTTATTAGTGATTTTCAAAAGGGGAGGGAGTGTACGAATAGGTGTGGGTCACAGAGATCACGTGCTTCACAAGGTAATAGAATATCACAAGGCAAATGGAGGCAGGGCGAGATCACAGGACCACAGGACCGGGGTGAAATTAAAATTGCTAATGAAGTTTTGGGCACCATTGTCATTGATAACATCTTATCAGGAGACAGGGTTTGAGAGACAACCGGTCTGACCAAAATTTATTAGGTGGGAATTTCCTCGTCCTAATAAGCCTGGGAGTGCTATGGGAGACTGGGGCTTATTTCATCCCGTAGAGGCAACCATAAAAGACAGGCCCAAGCGGCCATTTAGAGCCTCCCAGGACATTCTCTTTCTCAGGGTGTTCCTTGCTGAGAAAAAGAATTCAGATATTTCTCCATTTGCTTTTGAAAGAAGAGAAATATGGCTCTGTTCCCGGCTCACGGCGCAGATTTAAGGTTATCTCCTTTTCCCTGAACAATTGCTGTTATCCTGTTCTTTTTTCAAGGTGCCCAGATTTCATATTGTTCAAACACACATGCTTACAATTTGTGCAGTTAAGCAATATCACAGGGTCCTGAGGGACATACATCCTCCTCAGCTGACAGGATTAAGAGATTAAAGTAAAGACAGGCATAGGAAATCACAAGGGTATTGATTGGGGAAGTGATAAGTGTCCATGAAATCTTCACAATTTATGTTTAGAGATTGCAGTAAAGACAGGCATAAGAAATTATAAAAGTATTAATTTGGGGAACTAATAAATGTCCATGAAATCTTCACAATCCACGTTCTTCTGCCATGGCTTCAGCCGGTCCCTCCGTTCGGGGTCCCTGACTTCCCGCAACA
THE1A	TGATATGGCTTGGCTGTGTCCCCACCCAAATCTCATCTTGAATTGTACGTTCCATAATTCCCATGTGTTGTGGGAGGGACCCAGTGGGAGATAATTGAATCATGGGGAGCAGTTTCCCCCATACTGTTCTCATGGTAGTGAATAAGTCTCATGAGATCTGATGGTTTTATCAGGGGTTTCTACTTTTGCATCTTCCTCATTTTCCTCTTGCcACcACCATGTAAGAAGTGCCTTTCACCTCCcACCATGATTCTGAGGCCTCCCCAGCCATGTGGAACTGTAAGTCCAATTAAACTTCTTTTTCTTCCCAGTCTTGGGTATATCTTTATCAGCAGCATGAAAATGGACTAATACA
AluSx	TTTCTTTACTTCAACCTTCTTTTTTTTTTTGAGACAGAGTCTGCTCTCGTTGCCCAGGCTGGAGTGCAATGGCGCAACCTCAGCTCACTGCAACCTCTGCCTCCTGGGTTCAAGCAATTCTCCTGCCTCAGCCTCCCAAGTAGCTGGGATTACAGGCATGCGCCACCACGCCCAGCTAATTTTGTATTTTTAGTAGAGACGGGGTTTCACCATGTTGGCCAGGCTGGTCTTGAACTCCTGACCTCAGGTGATCCACCCGCCTCAGCCTCTCAAAGTGCTGGGATTACAGGCGTCAGCCACCGCACTCAGCC
AluSp mutants	

80 bp	CCAGGATGGAGTGCAAGGGATCTTGGCTCACCACAGCCTCCGCCTCTTGGGTTCAAGTGATTCTTCT GCCTCAGCCTCCC
**Pancreatic Cancer**	
AluSx1	TTATGACTATTATTTCTTTTTTTTTTTTTTGAGATGGAGTCTCGCTCTATTGCCCAGGCTGGAGTGCAGT GGCATGATCTCGGCTCACTGCAACCTCTGCCTCCCGGGTTCAAGCGATTCTCCTGCCTCAGCCTCCTG AGTAGCTGGGATTACAGGTGCCTGCCACCACACCCGGCTAATTTTTGTATTTTTAGTAGAGATGGGGT TTCACTGTGTTGGCCAGGATGGTCTTAAACTCCTGACCTCAAGTGATCCACCCACCTCGGCCTCCCAA AGTGTTGGGATTACAGGCGTGAGCCACCATGCCCGGCC
**Control**	
	GTGAACCGCATCGAGCTGAAGGGCATCGACTTCAAGGAGGACGGCAACATCCTGGGGCACAAG CTGGAGTACAACTTCAACAGCCACAACGTCTATATCATGGCCGACAAGCAGAAGAACGGCATCAAG GTGAACTTCAAGATCCGCCACAACATCGAGGACGGCAGCGTGCAGCTCGCCGACCACTACCAGCAG AACACCCCCATCGGCGACGGCCCCGTGCTGCTGCCCGACAACCACTACCTGAGCACCCAGTCCGCC CTGAGCAAAGACCCCAACGAGAAGCGCGAGAATTC
**Primers**	
Deletion mutants AluSp	
Forward Del1	GGGATTATAAGCACCCACCACCACG
Reverse Del 1	CCTGACCTCATGATCTGCCCACCG
Forward Del2 and 3	ACCCGGCCTTGGACACGCCATTTTC
Reverse Del 2	CCAGGATGGAGTGCAAGGGATCTTG
Reverse Del 3	GGGATTATAAGCACCCACCACCACG
mCherry	
Forward 1	ATGGTGAGCAAGGGCGAGGAGGATAA
Reverse 1	TCGGGGAAGGACAGCTTCAAGTAGT
Reverse 2	CTGGGTCACGGTCACCACGCCGCCGT
AluSq	
Forward	GGGATTATAAGCACCCACCACCACG
Reverse	CGTGGTGGTGGGTGCTTATAATCCC
Mutants of 80 bp from 5' end	

Position	Original sequence	Mutated sequence
AT mutant		
10-22	AGTGCAAGGGATC	AATATAAAAAAT
63-73	GATTCTTCTGCCTC	AATTTTTCTTATTT
CG mutant		
3-19	GGATGGAGTGCAAGGGATC	CCATGCACTCGAACCCATG
63-77	GATTCTTCTGCCTCAGCC	CATTGTTGTCGGTGAGGG

2. **<u>Constructs</u>**
   a. **<u>mCherry vector:</u>** Vector Builder and the cat# is VB201118-1319gwe
   b. **<u>pCMV-GFP vector:</u>** Addgene Cat#11153
