# Supplementary material for: Transposon DNA sequences facilitate the tissue-specific gene transfer of circulating tumor DNA between human cells
Source: Nucleic Acids Res. 2024 May 23;52(13):7539–55. doi: 10.1093/nar/gkae427 (PMC11260451; doi:10.1093/nar/gkae427)
Supplement: gkae427_Supplemental_Files [file gkae427_supplemental_files.zip › SUPPLEMENTAL METHODS COMBINE 4-10-24.pdf]

Supplemental Methods

Generation of ctDNA-GFP linear DNA and ctDNA-GFP experiment.

ctDNA from a newly diagnosed multiple myeloma patient was sonicated to 3000 bp using a covaries ultrasonicator instrument. After confirming correct size, ctDNA fragments were blunt end repaired using DNA repair enzyme (Quick Blunting™ Kit, New England Biolab, MA). Similarly, a pCMV-GFP vector (Cat# 11153, Addgene, MA), kindly donated by Dr. Shi-Yong Sun from Winsihp Cancer Institute of Emory University, was digested with SpeI to produce a linearized vector. After gel extraction, the vector underwent blunting using similar protocol as described above. linear CMV-GFP vector and sonicated ctDNA underwent blunt end ligation (Blunt/TA Ligase Master Mix). Subsequently, size selection and gel extraction were performed.

For the microscopy experiments, 5 $x10^5$ MM1s cells were cultured with 0.3 mg of ctDNA-CMV-GFP linear DNA. DNA was added directly to the culture media. Live images were obtained after 48 hours of culture using a Nikon Ti2-E – Crest X-Light Spinning Disc Confocal microscope.

Effect of reverse transcriptase and integrase inhibitors in ctDNA chromatid integration

Two million cells of multiple myeloma (MM1s), pancreatic cancer (ASPC-1), and colon cancer (HCT116) cell line were treated with 100nM Raltegravir 6μM Didanosine or 0.4μM Zidovudine for 48h. After 48h of drug treatment, rhodamine-labeled ctDNA was added and incubated for an additional 24h. At which point cells were arrested and metaphase chromosome spread were performed. Each experimental condition were replicated in three independent experiments. Ten metaphases per experiment were used to count the number of ctDNA integration on the chromatids.

Identification of tissue specific inserted transposons- Analysis pipeline

a. **Approach 1**

- **Sequencing raw data QC and preprocessing**

Sequencing data quality check was performed using FastQC [1] and multiQC tools [2]. Illumina adapters were trimmed using trimmomatic v0.39 [3].

- **De novo assembly of ct-DNA samples**

For de novo assembly of ct-DNA reads ABySS [4] de novo assembler was used. Before assembly, ct-DNA samples were 10x depth normalized with bbnorm [5]. The best k-mer size for the assembly was predicted with the KmerGenie tool [6]. In addition, ct-DNA samples used in cell line cultured experiments (772 and P201812-2) were assembled without any read depth normalization.

Cluster analysis of ct-DNA assembled contigs

Contig-level assembly sequences were used for cluster analysis. cd-hit-est-2d was used to select contigs specific for MM and PC [7] with 95% of identity as a threshold. cd-hit-est-2d compared to sequence datasets (db1 and db2) and reports sequences that are not similar in db2 as well as sequences that are similar between db1 and db2. Since we were interested in MM- and PC-specific contigs we performed cd-hit-est-2d twice: First, MM contig assemblies were assigned as db2 (for MM-specific contigs), and then PC contig assemblies were assigned as db2 (for PC-specific contigs).

- **Detection of de novo insertions in cell line samples**

All cell line samples were aligned to the human reference genome(hg38) with bwa-mem software (v 0.7.17) [8]. After the alignment step de novo insertions detection was performed with the Pamir tool [9]. Pamir uses “one-end anchor” reads (i.e. one-end is mapped while the other is unmapped around breakpoint location) and orphan reads (read pairs where none of the ends can be mapped to the reference) to characterize the novel sequence contents and their insertion breakpoints. Algorithm steps include de novo assembly, re-alignment, and clustering on mentioned reads to generate contigs for putative novel insertions. Aligned BAM files for cell culture sequences along with the hg38 genome reference were supplied to Pamir. The tool outputs a VCF file with the sequence, location, and length of identified novel insertions.

- **Selection of cancer-specific insertions**

To select cancer type-specific inserts, full-length contigs were converted to BLAST databases. Next insertions identified in the match-, mismatch- and no-culture samples for corresponding cell line samples (MM1S or MIA) were blasted against the corresponding sample full-length assembly

contig database (772 for MM1S cells and P201812-2 for MIA cells). An insert was considered cancer-specific if: 1) it was present in the matching co-culture sample, but not in mismatch co-culture and no-culture samples; 2) it was aligned to the corresponding ct-DNA sample database with identity at least 70% (to maximize hits for further processing).

- **Multiple sequence alignment and alignment-based contig reassembly and extension**

To define if the selected unique contigs are present in all samples, we have created a BLAST database for all samples and blasted the unique contigs against each database. The myeloma database consisted of contigs from 5 MM patients, for pancreatic cancer the database consisted of 10 PC patients. Unique contigs from each cancer type were blasted against the corresponding database. Then, we have selected the contigs, with alignment length >= 650bp and BLAST identity ≥90%. As a result, we have defined the unique contig sets, which were present in all samples belonging to one cancer type (MM1S - 14 contigs and MIA - 13 contigs). In the next step, we have aligned contigs in all samples with the corresponding insertion, using MAFFT [10] , and constructed the consensus sequences.

- **Short-read alignment-based contig re-assembly**

As the aligned contigs did not fully cover the whole full assembly contigs (772 and P201812-2), we have used consensus sequences, obtained in the previous step, as a reference to align fastq reads on it. The alignment was performed with the bwa-mem algorithm [8] and a final consensus was obtained for each unique contig. Alignment visualization and alignment consensus retrieval were performed with UGENE v41 [11].

- **Transposon identification**

We have used the final consensus obtained in the previous step, to identify repeats/transposons with RepeatMasker (RM) rmblast [12]. Then we aligned hit repeats/TE sequences, unique contigs for all samples, and insertion sequence with MAFFT. Mutations in TE sequences were identified by comparison of nucleotides at each position in the multiple sequence alignment with the "Biostrings" R package (version 4.1). We considered a position to contain mutation if the substitution was present in contigs of all samples. In the case of ambiguous nucleotides in contigs introduced by short read alignment (putative heterozygosity in the sample), non-matched

nucleotide was considered as a mutation if it was present in all samples. Finally, based on the identified mutations, we have constructed the mutated transposon sequences for each unique contigs (for MM1S and MIA datasets).

- **Transposon expression variability analysis**

Single-end RNA-seq data for MM (n=60) and PC (n=22) samples were processed with the STAR aligner and TEtranscripts pipeline [13]. hg38 and Gencode and TE-specific curated annotations were used as reference genome and gene/TE annotations, respectively. Raw counts were processed using the DESeq2 R package [14]. Low count genes and TEs (less than 1000 read in total for all samples) were removed. Counts across samples were normalized for library sizes and log-transformed using 'regularized log' transformation. Expression variability (EV) of TEs and genes (probes) was estimated using the previously described method [15,16]. EV estimation allows for estimating the expression-independent variability. We have used two parameter sets: 1) median/median absolute deviation (MAD), and 2) mean/standard deviation (SD). First, a bootstrapped estimate of the MAD/SD of each probe was calculated using 1000 bootstrap replicates. Next, the expected MAD/SD as a function of median/mean was calculated using local polynomial regression (loess R function). Expression variability (EV) was calculated as the difference between the bootstrapped MAD/SD and the expected MAD/SD for each median/mean expression level. EV, in this case, shows the level of variability for a particular probe compared to other probes with the same expression values. The empirical distribution of EV (ecdf R function) was used to estimate the significance of variability (PEV).

First, we calculated magnitude-independent expression-variability for all probes (genes and TEs). Next, we regressed expression values - variance using local polynomial regression to estimate expected variability and estimated the deviance of observed variance for each probe (expression variability, EV) (figure 4). Negative EV values indicate lower variance compared to other probes with a similar expression, and positive EV values show a higher variance. Empirical distribution of EV was also used to calculate the lower-tail p values for each probe. Following thresholds have been defined for highly expressed invariant Tes (HEI Tes): log-transformed Mean/Median expression ≥ 10 (corresponds to 1024 reads), PEV < 0.1.

b. Approach 2

- **De novo assembly and evaluation of the quality of assembly**

We used String Graph Assembler (SGA) to assemble genomes of cells, ctDNA and coculture. SGA is a de novo genome assembler which is memory efficient using a compressed representation of DNA sequences. First, we used a pre-QC step that is recommended as part of the SGA pipeline. The preQC module estimate sequence coverage, per-base error rates and genome size, heterozygosity and repeat content. The details of the preQC module are detailed here- https://arxiv.org/abs/1307.8026. The pre-processed data is then indexed, and base calling errors are corrected, duplicate and low-quality reads are removed. A string graph of the QCed reads is then constructed and contig assembly is performed. For contig assembly, a minimum overlap value of 77 was used. Aggressive variant removal parameters were chosen and small repeats at the ends of reads were resolved using the "-r 10" parameter. The minimum branch length for the trimming algorithm was 200 bp. After assembly, the contigs were then combined in a scaffold. The scaffolding stage realigns the original reads to the contigs using BWA, constructs a scaffold graph using the alignments, and outputs a final set of scaffolds in FASTA format. After scaffolding, we used contigs greater than 300bp for the identification of SVs and SNVs.

The summary statistics from the SGA alignment of the WGS samples is provided in Supplementary Methods Table 1. The WGS samples were analyzed using QUAST to generate these statistics (https://github.com/ablab/quast). Figure 1 shows the number of overall contigs generated by the assembly and the contigs greater than 300 base pairs. SGA generated a similar number of contigs for all the samples.

Supplementary Methods Table 1. Summary statistics from the SGA assembly of the WGS samples (these were generated by QUAST)

Assembly	MIA_cells	MM1S_cells	772	P201812_2	MIA_cells_with_P201812_2	MM1S_cells_with_772_2	54128_4M7	54128_6MP
# contigs (>= 0 bp)	2179175	2059217	6414730	3018986	1925809	2256253	2030030	1820622
# contigs (>= 1000 bp)	468395	271334	453358	552700	242168	277998	475701	302253
# contigs (>= 5000 bp)	172066	139528	155	169878	126418	137172	179241	150673
# contigs (>= 10000 bp)	70242	83453	0	57883	79139	81710	69405	84697
# contigs (>= 25000 bp)	7533	25392	0	4013	28649	25420	6265	21573
# contigs (>= 50000 bp)	287	4908	0	81	7220	5118	160	3292
Total length (>= 0 bp)	3117997927	3150837102	2901047692	3336143579	3119008957	3193912826	3100268940	3091833942
Total length (>= 1000 bp)	2619315387	2664732387	640989556	2596220928	2673174471	2662318825	2639219576	2663379736
Total length (>= 5000 bp)	1885483586	2339520597	874330	1675768750	2389234524	2318539334	1893021143	2283016481
Total length (>= 10000 bp)	1167263718	1936254650	0	891997498	2049656683	1920536039	1118819833	1809271173
Total length (>= 25000 bp)	244869092	1021588845	0	124792708	1247383978	1032630528	198999791	827681869
Total length (>= 50000 bp)	16776279	327091126	0	4663067	508185725	342729065	9255682	213708388
# contigs	610692	360972	1805989	783821	320451	373417	613885	391140
Largest contig	114993	197261	8016	85264	254016	259733	91133	175091
Total length	2719635652	2726528266	1571883071	2757933353	2727304755	2728205037	2736497457	2724960601
Reference length	3101804739	3101804739	3101804739	3101804739	3101804739	3101804739	3101804739	3101804739
GC (%)	40.84	40.83	39.42	41	40.83	40.8	40.93	40.86
Reference GC (%)	40.9	40.9	40.9	40.9	40.9	40.9	40.9	40.9
N50	8440	18431	888	6599	22398	18515	8148	15578
NG50	7095	15387	507	5644	18563	15379	6967	13081
N75	4121	8628	664	3167	10066	8377	4149	7480
NG75	2576	5158	607426	2073	5898	4906	2716	4556
L50	91218	41335	1764316	118201	33566	40860	97071	48899
LG50	115922	52485	1122092	146371	42736	51936	121320	62103
L75	206013	95148	3869	268158	78723	95428	214174	111761
LG75	293301	136937	3834	368195	114845	138750	295206	159754
# misassemblies	1319	1745	3692151	1389	1894	1968	1337	1541
# misassembled contigs	1181	1589	1448	1299	1718	1806	1225	1420
Misassembled contigs length	6802306	19130093	0	6009073	24137076	18856255	6327978	15690428
# local misassemblies	3179	4447	313	3147	4105	4686	3233	4283
# scaffold gap ext. mis.	1	72	404	1	118	108	0	39
# scaffold gap loc. mis.	9	6037	7544 + 902 pa	1279	13607	12607	0	3852
# unaligned mis. contigs	1028	1211	6622400	1039	1221	1359	1123	1157
Unaligned length	16226435	19089542	1.017	82179279	19972628	18194649	22581617	22193530
Genome fraction (%)	92.862	93.568	2.21	92.172	93.486	93.447	93.112	93.533
Duplication ratio	1.016	1.01	105.85	1.013	1.011	1.012	1.017	1.009
# N's per 100 kbp	1.77	130.14	19.85	13.12	225.72	206.62	0	63.87
# mismatches per 100 kbp	105.41	122.02	8016	99.36	108.78	123.02	104.25	118.3
# indels per 100 kbp	23.48	31.98	1563087880	22.24	33.17	34.87	23.39	28.32
Largest alignment	114993	197236	884	85264	254016	259606	91133	175091
Total aligned length	2701484908	2705060536	503	2672732084	2704403204	2706854378	2711934622	2700677926
NA50	8397	18287	659	6481	22203	18357	8088	15425
NGA50	7048	15249	609379	5523	18352	15229	6911	12928
NA75	4076	8501	1773934	3033	9892	8243	4087	7333
NGA75	2525	5030	1126789	1920	5713	4772	2644	4402
LA50	91597	41581		119677	33816	41120	97649	49280
LGA50	116444	52825		148418	43080	52295	122087	62625
LA75	207309	95973		274146	79558	96317	215993	113033
LGA75	295955	138595		380203	116538	140537	298697	162318

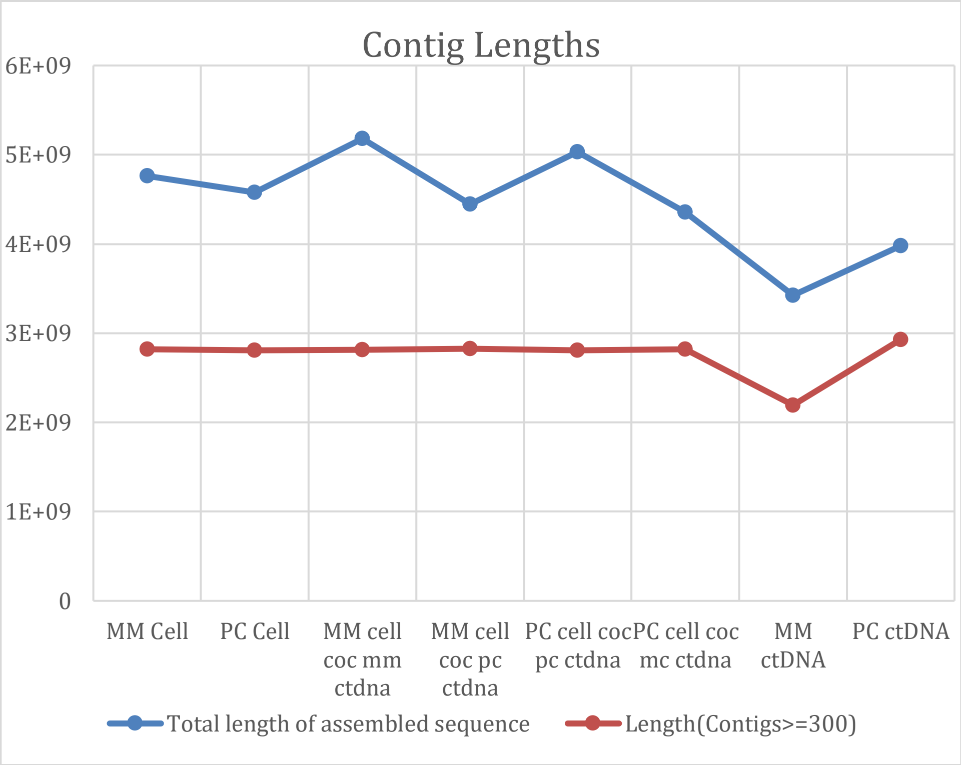

Figure 1. Contigs length between experimental samples.

- **Cluster analysis of ctDNA assembled contigs**

Contig-level assembly sequences were used for cluster analysis. cd-hit-est-2d was used to select contigs specific for MM and PC [7] with 95% of identity as a threshold. cd-hit-est-2d compared to sequence datasets (db1 and db2) and reports sequences that are not similar in db2 as well as sequences that are similar between db1 and db2. Since we were interested in MM- and PC-specific contigs we performed cd-hit-est-2d twice: first, MM contig assemblies were assigned as db2 (for MM-specific contigs), and then PC contig assemblies were assigned as db2 (for PC-specific contigs).

- **Detection of de novo insertions in cell line samples**

All cell line samples were aligned to the human reference genome(hg38) with bwa-mem software (v 0.7.17) [8]. After the alignment step *de novo* insertions detection was performed with the Pamir tool [9]. Pamir uses "one-end anchor" reads (i.e. one-end is mapped while the other is unmapped

around breakpoint location) and orphan reads (read pairs where none of the ends can be mapped to the reference) to characterize the novel sequence contents and their insertion breakpoints. Algorithm steps include de novo assembly, re-alignment, and clustering on mentioned reads to generate contigs for putative novel insertions. Aligned BAM files for cell culture sequences along with the hg38 genome reference were supplied to Pamir. The tool outputs a VCF file with the sequence, location, and length of identified novel insertions.

Selection of cancer-specific insertions

To select cancer type-specific inserts, full-length contigs were converted to BLAST databases. Next insertions identified in the match-, mismatch- and no-culture samples for corresponding cell line samples (MM1S or MIA) were blasted against the corresponding sample full-length assembly contig database (772 for MM1S cells and P201812-2 for MIA cells). An insert was considered cancer-specific if: 1) it was present in the matching co-culture sample, but not in mismatch co-culture and no-culture samples; 2) it was aligned to the corresponding ctDNA sample database with identity at least 70% (to maximize hits for further processing).

- **Multiple sequence alignment and alignment-based contig reassembly and extension**

To define if the selected unique contigs are present in all samples, we have created a BLAST database for all samples and blasted the unique contigs against each database. The myeloma database consisted of contigs from 5 MM patients, for pancreatic cancer the database consisted of 10 PC patients. Unique contigs from each cancer type were blasted against the corresponding database. Then, we have selected the contigs, with alignment length >= 650bp and BLAST identity ≥90%. As a result, we have defined the unique contig sets, which were present in all samples belonging to one cancer type (MM1S - 14 contigs and MIA - 13 contigs). In the next step, we have aligned contigs in all samples with the corresponding insertion, using MAFFT [10], and constructed the consensus sequences.

- **Short-read alignment-based contig re-assembly**

As the aligned contigs did not fully cover the whole full assembly contigs (772 and P201812-2), we have used consensus sequences, obtained in the previous step, as a reference to align fastq reads on it. The alignment was performed with the bwa-mem algorithm [8] and a final consensus was

obtained for each unique contig. Alignment visualization and alignment consensus retrieval were performed with UGENE v41 [11].

To visualize ctDNA integration to cell line sequences, we aligned the cell line and ctDNA contigs against the coculture contigs, where Nucdiff reported an insertion event, using NCBI's nucleotide blast query. Visualizing the alignment (Figure 3), we identified transition points, where the ctDNA integrated to cell line DNA. To test homology between the inserted ctDNA and the neighboring cell line DNA, we compared cell line DNA at the transition point with inserted ctDNA (as reference, Supplementary Figure 4) using BLAST.

- **<u>Pathway analysis and annotation of oncogenes</u>**

Pathway analysis: Genes that harbor inserted ctDNA sequences were annotated using genomic co-ordinates from build 37 of the human genome. Enrichment of gene-ontology pathways terms were tested in the identified genes using the R clusterProfiler (https://bioconductor.org/packages/release/bioc/html/clusterProfiler.html). Significance was established using an FDR corrected p-value ($p<0.05$). This analysis was conducted to identify putative cancer biological processes that are enriched amongst candidate genes that harbor insertions from ctDNA.

Annotating Oncogenes: We used OncoKB database [17] created by the Memorial Sloan Kettering Hospital to determine oncogenes from candidate genes that harbor inserted ctDNA sequences. We used a list of 1130 genes as candidate oncogenes listed in OncoKB as of 10/02/2023.

- **Transposon identification**

To determine the locations of transposable-like regions in the contigs, sequences were analyzed, and transposable elements (TEs) were identified and classified using RepeatMasker version 4.1.0[18] and . The Dfam database (release 3.1 [19]) of repetitive DNA families was used as a reference for identifying repeats in ctDNA contig sequences that were part of qualifying structural events as described above. For each repeat sequence identified by RepeatMasker, we computed the overall frequency of the specific repeat (e.g., for AluSp or L1) and their parent class (e.g., SINE).

Once the TE elements were identified, we aligned hit repeats/TE sequences, unique contigs for all samples, and insertion sequence with MAFFT software version 7. Mutations in TE sequences were identified by comparison of nucleotides at each position in the multiple sequence alignment with the “Biostrings” R package (version 4.1). We considered a position to contain mutation if the substitution was present in contigs of all samples. In the case of ambiguous nucleotides in contigs introduced by short read alignment (putative heterozygosity in the sample), non-matched nucleotide was considered as a mutation if it was present in all samples. Finally, based on the identified mutations, we have constructed the mutated transposon sequences for each unique contigs (for MM1S and MIA datasets).

Transposon expression variability analysis

- **Data preprocessing**

Single-end RNA-seq data for MM (n=60) and PC (n=22) samples were processed with the STAR aligner and TEtranscripts pipeline [13]. hg38 and Gencode and TE-specific curated annotations were used as reference genome and gene/TE annotations, respectively. Row counts were processed using DESeq2 R package [14]. Low count genes and TEs (less than 1000 read in total for all samples) were removed. After pre-filtering 1021 TEs and 20251 genes remained. Counts across samples were normalized for library sizes and log-transformed using 'regularized log' transformation. Since MM samples were processed in two batches, we performed batch normalization on log-transformed data with ComBat function from sva R package [20] (Supplemental Methods Figure 1-3). No batch correction was performed for PC samples as sequencing was performed in a single batch

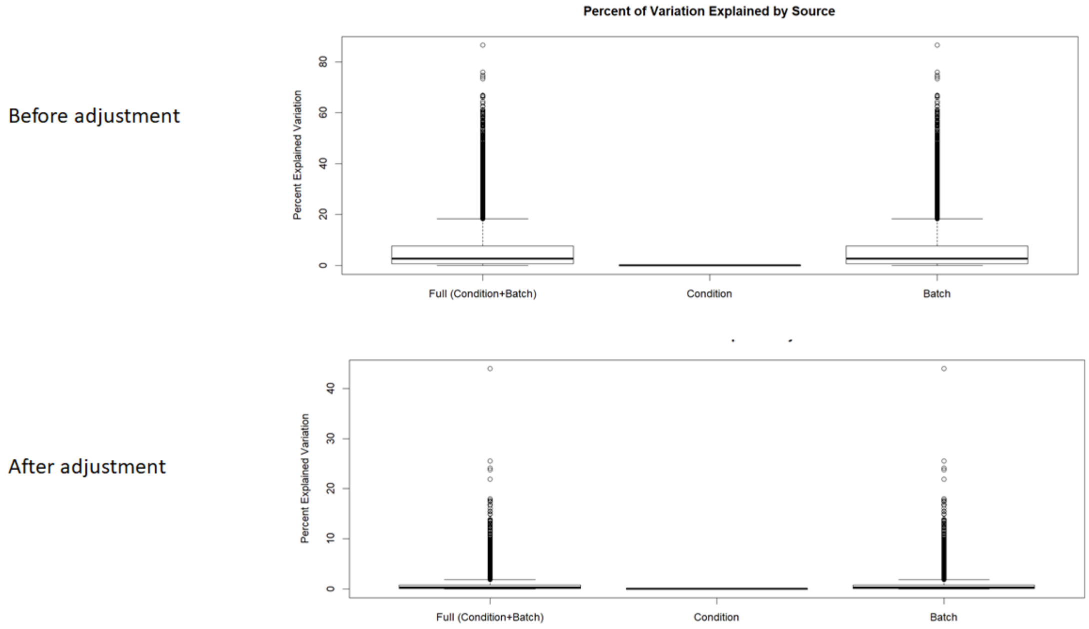

Supplemental Methods Figure 1. Batch-related variance before and after ComBat transformation (21272 genes + TEs).

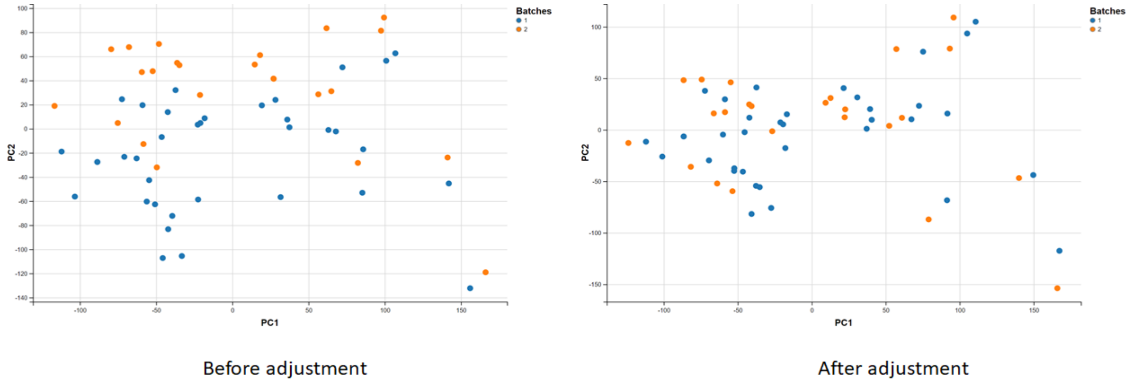

Supplemental Methods Figure 2. PCA plots before and after ComBat transformation in MM (21272 genes + TEs)

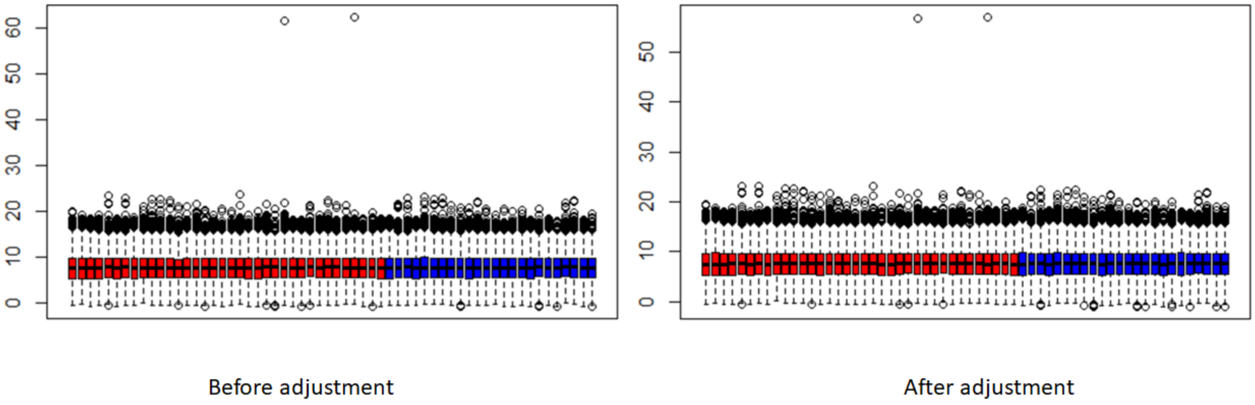

Supplemental Methods Figure 3. Expression boxplot before and after ComBat transformation in MM (21272 genes + TEs)

- **Expression variability estimation**

Expression variability (EV) of TEs and genes (probes) was estimated using the previously described method [15,16]. EV estimation allows for estimating the expression-independent variability. First, we calculated median/MAD magnitude-independent expression-variability for all probes (genes and TEs). Then, we regressed expression values - variance using local polynomial regression to estimate expected variability and estimated the deviance of observed variance for each probe (expression variability, EV) (Supplemental Methods Figure 4).

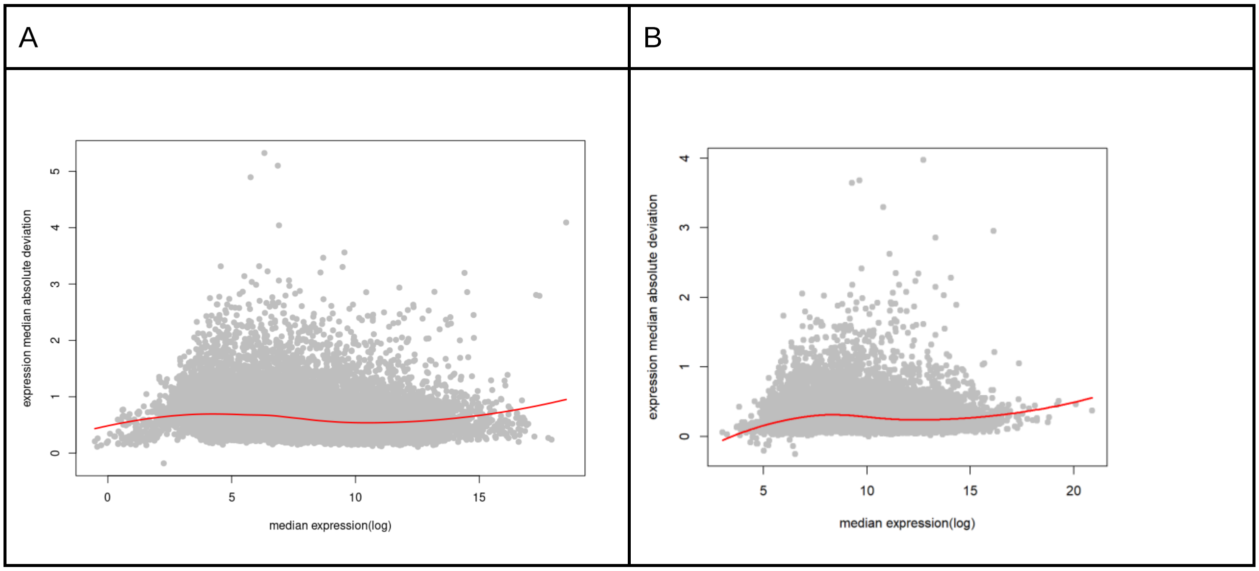

Supplemental Methods Figure 4. Bootstrapped (grey dots) and regressed variation (red line) for median/MAD estimator in MM (A) and PC (B).

Expression variability (EV) was calculated as the difference between the bootstrapped MAD and expected MAD each median expression level. EV in this case shown the level of variability for a particular probe compared to other probes with the same expression values. The empirical distribution of EV (*ecdf* R function) was used to estimate the significance of variability.

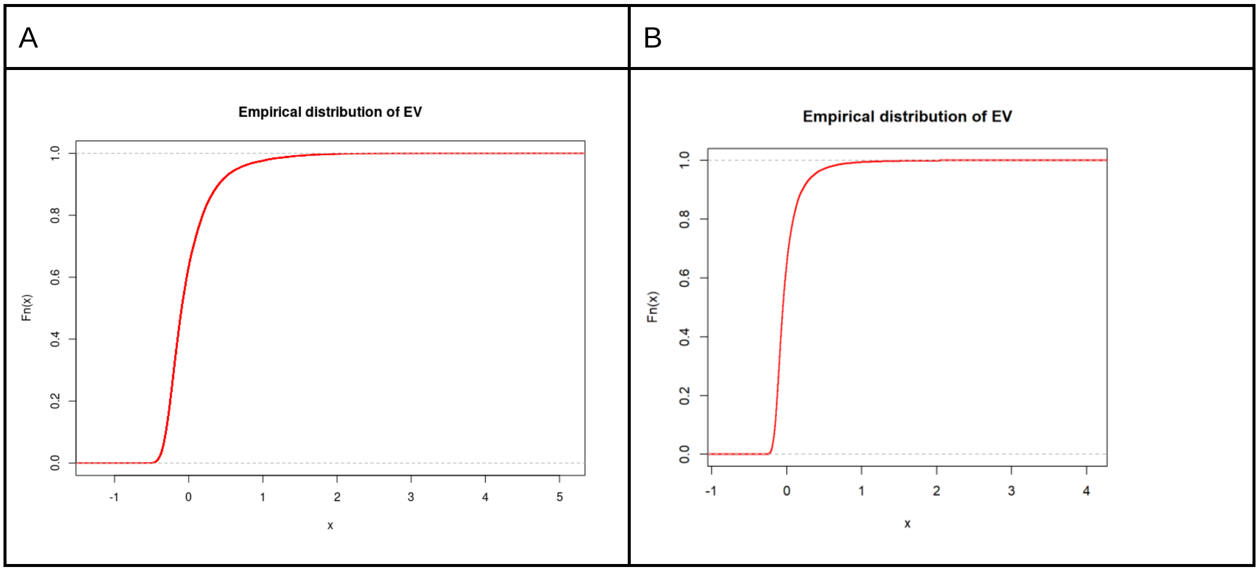

Supplemental Methods Figure 5. Empirical distribution of expression variability for median/MAD in MM (A) and PC (B).

- **Identification of highly expressed invariant (HEI) TEs (with Mean/SD)**

First, we calculated magnitude-independent expression-variability for all probes (genes and TEs). Next, we regressed expression values - variance using local polynomial regression to estimate expected variability and estimated the deviance of observed variance for each probe (expression variability, EV) (Supplemental Methods Figure 5). Negative EV values indicate lower variance compared to other probes with a similar expression, and positive EV values show the higher variance. Empirical distribution of EV was also used to calculate the lower-tail p values for each probe.

Following thresholds have been defined for HEI TEs: log-transformed Mean/Median expression ≥ 10 (corresponds to 1024 reads), $P_{EV} < 0.05$. Only one TE satisfied these criteria. With less stringent criteria $P_{EV} \leq 0.1$, 6 additional TEs were identified (Supplemental Methods Table 2 and 3).

Supplemental Methods Table 2. HEI TEs (Mean/MAD EV assessment) in MM.

Probes	gene_id	class_id	Median	Obs.MAD	Exp.MAD	EV	EV.pvalue
AluY:Alu:SINE	AluY	SINE	16.68	0.41	0.78	-0.38	0.030
MIRb:MIR:SINE	MIRb	SINE	16.42	0.29	0.76	-0.47	0.001
L2a:L2:LINE	L2a	LINE	16.26	0.41	0.75	-0.34	0.057
MIR:MIR:SINE	MIR	SINE	15.92	0.35	0.73	-0.38	0.028
L2c:L2:LINE	L2c	LINE	15.81	0.37	0.72	-0.35	0.050
L2b:L2:LINE	L2b	LINE	15.66	0.29	0.71	-0.42	0.011
MIRc:MIR:SINE	MIRc	SINE	15.25	0.28	0.68	-0.41	0.014
MIR3:MIR:SINE	MIR3	SINE	15.24	0.28	0.68	-0.41	0.015
L1M5:L1:LINE	L1M5	LINE	13.85	0.30	0.61	-0.31	0.093
L1MC3:L1:LINE	L1MC3	LINE	12.21	0.22	0.56	-0.34	0.057

* Full data is available in '0.rlogcpm.mad.xlsx' file.

Supplemental Methods Table 3. HEI TEs (Mean/MAD EV assessment) in PC.

Probes	gene_id	class_id	Median	Obs.MAD	Exp.MAD	EV	EV.pvalue
L1ME4a:L1:LINE	L1ME4a	LINE	11.58	0.068	0.242	-0.174	0.061
AluSz6:Alu:SINE	AluSz6	SINE	11.34	0.075	0.245	-0.169	0.069
AluSc:Alu:SINE	AluSc	SINE	10.59	0.065	0.262	-0.195	0.026
AluSx3:Alu:SINE	AluSx3	SINE	10.46	0.073	0.265	-0.191	0.032
OldhAT1:hAT-Ac:DNA	OldhAT1	DNA	10.16	0.116	0.274	-0.158	0.098
MamGyp-int:Gypsy:LTR	MamGyp-int	LTR	10.14	0.052	0.275	-0.223	0.007
L1MB3:L1:LINE	L1MB3	LINE	10.01	0.097	0.279	-0.182	0.046

* Full data is available in '0.PC.TE.rlogcpm.mad.xls' file.

- **Identification of highly expressed invariant (HEI) TEs (with Median/MAD)**

Heatmap clustering confirmed that selected TEs have high expression values (Figure 4b and c of the manuscript). Next, we generated random datasets of TEs by 100 bootstrap replicates for each gene and performed clustering for each dataset (file: 0.clustering.pdf). In the majority of datasets, the HEI TEs cluster together.

Synthesis of DNA Transposon Sequences and Reporter Assay Experiments

A polynucleotide comprising sequences corresponding to the transposon that contained mutations shared by all the MM samples was generated by Integrated DNA Technologies, Inc (IDT). The majority of the transposons used were selected because of their ability to be generate as gBlock except for AluSq that required to be produced in 2 blocks to overcome interference from the poly T segment. Additionally, in the AluSq an EcoRI complementary site was added at the end to facilitate ligation to Cytomegalovirus-red fluorescent protein (CMV-mCherry) or -herpes simplex virus thymidine kinase (HSV-TK) linearized vectors.

To evaluate the capacity of cell targeting, all synthetic DNA retrotransposon sequences were reconstituted in sterile water before labeling with CY5 following manufacturer protocol (Mirus Cat#). CY5-DNA retrotransposon was then added to the cell culture directly without adding any additives. Similarly, AluSp sequence containing a ECORI restriction site at the 3'end was used to ligate it in both ends of a linearized CMV-mCherry or CMV- HSV-TK vector. Agarose gel electrophoresis was used for size selection and extraction. Purified AluSp-CMV-mCherry or -HSV-TK were added to culture media directly for 24 and 72 hours correspondingly.

Generation of Transposon mutants and internalization measurement

AluSp sequence described in Supplemental Table 5 was used as template. To generate deletion mutants and point mutations described in Supplemental Table 5 we use a polymerase chain reaction method. Primers used to generate these mutations are described in Supplemental Table 5.

Deletion and point mutants were labeled with CY5 using similar methods as described above. CY5-transposon (+) cells were measured by flow cytometry methods.

References

1. FastQC: A Quality Control Tool for High Throughput Sequence Data [Online]. Available online at: http://www.bioinformatics.babraham.ac.uk/projects/fastqc/ (2015), "FastQC," https://qubeshub.org/resources/fastqc.
2. Ewels, P., Magnusson, M., Lundin, S. & Käller, M. MultiQC: summarize analysis results for multiple tools and samples in a single report. *Bioinformatics* **32**, 3047-3048 (2016).

3. Bolger, A.M., Lohse, M. & Usadel, B. Trimmomatic: a flexible trimmer for Illumina sequence data. *Bioinformatics* **30**, 2114-2120 (2014).
4. Simpson, J.T.*, et al.* ABySS: a parallel assembler for short read sequence data. *Genome Res* **19**, 1117-1123 (2009).
5. https://jgi.doe.gov/data-and-tools/software-tools/bbtools/.
6. Chikhi, R. & Medvedev, P. Informed and automated k-mer size selection for genome assembly. *Bioinformatics* **30**, 31-37 (2014).
7. Li, W. & Godzik, A. Cd-hit: a fast program for clustering and comparing large sets of protein or nucleotide sequences. *Bioinformatics* **22**, 1658-1659 (2006).
8. Li, H. & Durbin, R. Fast and accurate short read alignment with Burrows-Wheeler transform. *Bioinformatics* **25**, 1754-1760 (2009).
9. Kavak, P.*, et al.* Discovery and genotyping of novel sequence insertions in many sequenced individuals. *Bioinformatics* **33**, i161-i169 (2017).
10. Nakamura, T., Yamada, K.D., Tomii, K. & Katoh, K. Parallelization of MAFFT for large-scale multiple sequence alignments. *Bioinformatics* **34**, 2490-2492 (2018).
11. Okonechnikov, K., Golosova, O. & Fursov, M. Unipro UGENE: a unified bioinformatics toolkit. *Bioinformatics* **28**, 1166-1167 (2012).
12. Tarailo-Graovac, M. & Chen, N. Using RepeatMasker to identify repetitive elements in genomic sequences. *Curr Protoc Bioinformatics* **Chapter 4**, 4.10.11-14.10.14 (2009).
13. Jin, Y., Tam, O.H., Paniagua, E. & Hammell, M. TEtranscripts: a package for including transposable elements in differential expression analysis of RNA-seq datasets. *Bioinformatics* **31**, 3593-3599 (2015).
14. Love, M.I., Huber, W. & Anders, S. Moderated estimation of fold change and dispersion for RNA-seq data with DESeq2. *Genome Biol* **15**, 550 (2014).
15. Bashkeel, N., Perkins, T.J., Kærn, M. & Lee, J.M. Human gene expression variability and its dependence on methylation and aging. *Bmc Genomics* **20**, 941 (2019).
16. Alemu, E.Y., Carl, J.W., Jr., Corrada Bravo, H. & Hannenhalli, S. Determinants of expression variability. *Nucleic Acids Res* **42**, 3503-3514 (2014).
17. Chakravarty, D.*, et al.* OncoKB: A Precision Oncology Knowledge Base. *JCO Precis Oncol* **2017**(2017).
18. Smit, A., Hubley, R. & Green, P. RepeatMasker Open-3.0. . *(http://www.repeatmasker.org)* (1996-2004).
19. Hubley, R.*, et al.* The Dfam database of repetitive DNA families. *Nucleic Acids Res* **44**, D81-89 (2016).
20. Leek JT, J.W., Parker HS, Fertig EJ, Jaffe AE, Zhang Y, Storey JD, Torres LC sva: Surrogate Variable Analysis. *R package version 3.48.0.* (2023).
