# Supplementary material for: Transposon DNA sequences facilitate the tissue-specific gene transfer of circulating tumor DNA between human cells
Source: Nucleic Acids Res. 2024 May 23;52(13):7539–55. doi: 10.1093/nar/gkae427 (PMC11260451; doi:10.1093/nar/gkae427)
Supplement: gkae427_Supplemental_Files [file gkae427_supplemental_files.zip › Supplemental Video legends.pdf]

Supplemental Video 1A and B. Rotational images of 3D reconstruction of cellular and nuclear capture of rhodamine-labeled PC ctDNA in ASPC1 cells (B) and MM ctDNA in MM1s (A). Images show cellular localization of ctDNA. Membrane identified by bright field (gray color) and ctDNA (yellow color).

Supplemental Video 2. A. Slide image of ASPC1 demonstrating the capturing and nuclear localization of rhodamine-labeled PC ctDNA. **B.** Different slices of Z-stack images ASPC1 cells demonstrating ctDNA capturing in the cell membrane and invagination of cell membrane for internalization of ctDNA. **C.** 3D video reconstruction of B. Cell membrane was labeled using CellLight Plasma Membrane GFP kit (TermoFisher Scientific, MA)

Supplemental Video 3 A and B. 3D reconstruction of colocalization of match (MM-rhodamine – Red) and unmatched (PC-CY5 – green) ctDNA coculture with MM1s cells.
